# In Vitro Anti-Inflammatory Terpenoid Glycosides from the Seeds of *Dolichos lablab*

**DOI:** 10.3390/molecules30081779

**Published:** 2025-04-15

**Authors:** Wei Zhang, Jingya Ruan, Jiaming Cheng, Yingying Wang, Yinuo Zheng, Minghao Lin, Yi Zhang, Tao Wang

**Affiliations:** 1 Tianjin Key Laboratory of Tianjin University of Traditional Chinese Chemistry and Analysis, Tianjin University of Traditional Chinese Medicine, 10 Poyanghu Road, West Area, Tuanbo New Town, Jinghai District, Tianjin 301617, China; zhangwei940905@163.com (W.Z.); c1584172707@163.com (J.C.); zhengyinuo1002@163.com (Y.Z.); linminghao02@163.com (M.L.); 2 Institute of Tianjin University of Traditional Chinese, Tianjin University of Traditional Chinese Medicine, 10 Poyanghu Road, West Area, Tuanbo New Town, Jinghai District, Tianjin 301617, China; ruanjingya@tjutcm.edu.cn (J.R.); 15007283268@163.com (Y.W.)

**Keywords:** *Dolichos lablab* seeds, terpenoid glycosides, anti-inflammation, NO, TNF-α, IL-Iβ

## Abstract

To further explore the anti-inflammatory components of the seeds of *Dolichos lablab* L., a comprehensive phytochemical investigation was conducted using diverse chromatographic and spectrometric technologies, as well as chemical reactions. As a result, ten previously unreported terpenoid glycosides, namely dolilabterpenosides A, B, C_1_–C_3_, D, E, and F_1_–F_3_ (**1**–**10**), along with four known analogues (**11**–**14**), initially identified from *Dolichos* genus, were obtained. In addition, the lipopolysaccharide (LPS)-induced RAW264.7 cell model was employed to detect the expression levels of nitric oxide (NO), inflammatory cytokines tumour necrosis factor (TNF)-α and interleukin (IL)-1β to assess the anti-inflammatory activities of the obtained compounds. The results of bioactive assay showed that compounds **1**, **4**–**7**, and **10**–**12** showed significant inhibitory activity on NO release in RAW264.7 cells in a dose-dependent manner, and all of them were demonstrated to inhibit the increase in TNF-α and IL-Iβ levels in the supernatant of RAW264.7 cells stimulated by LPS.

## 1. Introduction

Inflammation represents the body’s immune defence response against detrimental stimuli. Controlled inflammation is advantageous for the body to restore normal physiological states. However, excessive or prolonged inflammatory reaction can result in systemic inflammatory response syndrome, autoimmune diseases and numerous chronic diseases, such as inflammatory bowel disease, rheumatoid arthritis, diabetes, Alzheimer’s disease, and atherosclerosis [1]. Currently, the principal drugs employed for treating inflammation in clinical practice are nonsteroidal anti-inflammatory drugs. Even though these drugs have certain therapeutic effects, they can easily lead to adverse reactions in gastrointestinal, cardiovascular, liver, kidney, and other organs [2]. Traditional Chinese medicine (TCM), with the property of medicine and food homology, has the advantages of high safety and few side effects, as well as holding significant medicinal value for chronic inflammation and intractable diseases. Hence, the search for anti-inflammatory components in medicinal and edible plants has been becoming a hot topic in modern Chinese medicine research.

The seed of *Dolichos lablab* L., belonging to Fabaceae family, *Dolichos* genus, is a common TCM. In TCM clinical practice, *D. lablab* is commonly used for the treatment of diseases closely related to inflammation, such as diarrhea and edema. It was reported to contain various nutritional components, such as polysaccharides, proteins, lipids, vitamins, minerals, volatile components, aromatic compounds, triterpenes, steroids, and flavonoids [3,4]. In vitro anti-inflammatory study had demonstrated that the methanol extract of *D. lablab* seeds exhibited significant anti-inflammatory activity [5]. Moreover, in vivo experiment discovered that its ethanol extract exerted a protective effect on irritable bowel syndrome in mice through reducing the expression levels of inflammatory factors such as tumour necrosis factor (TNF)-α and interleukin (IL)-6 [6]. Our previous study suggested that aromatic compounds in *D. lablab* seeds possess anti-inflammatory activity [4].

However, up to now, the scarce reports on the material basis of *D. lablab* have restricted its in-depth research and development. Therefore, the phytochemistry study of *D. lablab* was carried out in this study. Additionally, a lipopolysaccharide (LPS)-induced RAW264.7 macrophage model was established to assess the anti-inflammatory efficacy of the identified compounds through the quantification of nitric oxide (NO) and interleukin (IL)-1β levels in RAW264.7 cells.

## 2. Results and Discussion

### 2.1. Phytochemical Investigation Results and Discussion

**Figure 1:**
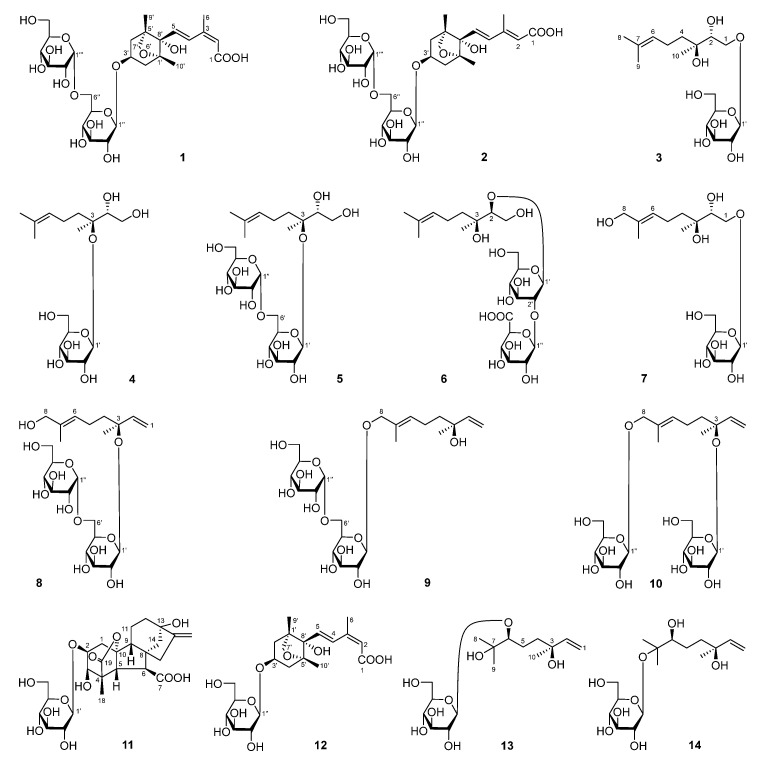
The structures of compounds **1**–**14** obtained from *D. lablab* seeds.

To further explore the anti-inflammatory components from *D. lablab* seeds, a comprehensive phytochemical investigation was conducted using diverse chromatographic and spectrometric methods. As a result, fourteen terpenoid glycosides, including ten previously unreported ones, namely dolilabterpenosides A, B, C_1_–C_3_, D, E, and F_1_–F_3_ (**1**–**10**), along with four known analogues, GA_8_-2-*O*-β-d-glucopyranoside (**11**) [7], (1′*R*,3′*S*,5′*R*,8′*S*,2*Z*,4*E*)-dihydrophaseic acid (**12**) [8], 3,7-dimethyl-oct-1-en-3,6,7-triol-6-*O*-β-d-glucopyranoside (**13**) [9], and (3*S*)-6,7-dihydroxy-dihydrolinalool-3-*O*-β-glucopyranoside (**14**) [10], were isolated and identified (Figure 1).

**Figure 2:**
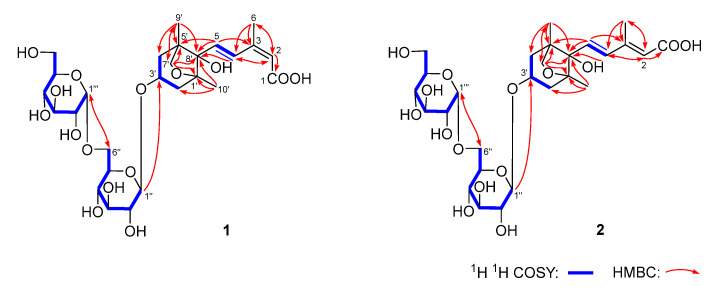
The main ^1^H ^1^H COSY and HMBC correlations between compounds **1** and **2**.

**Figure 3:**
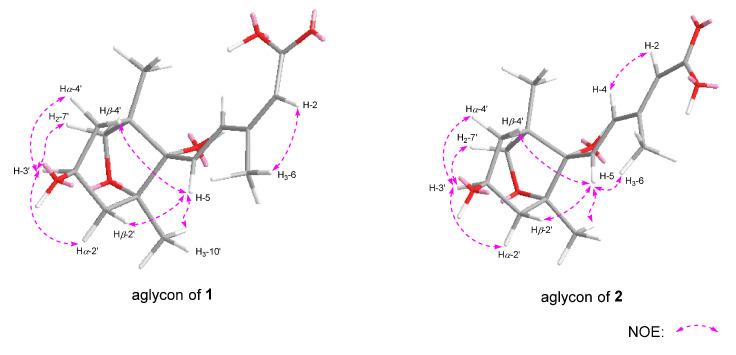
The main NOE correlations between the aglycone parts of compounds **1** and **2**.

**Figure 4:**
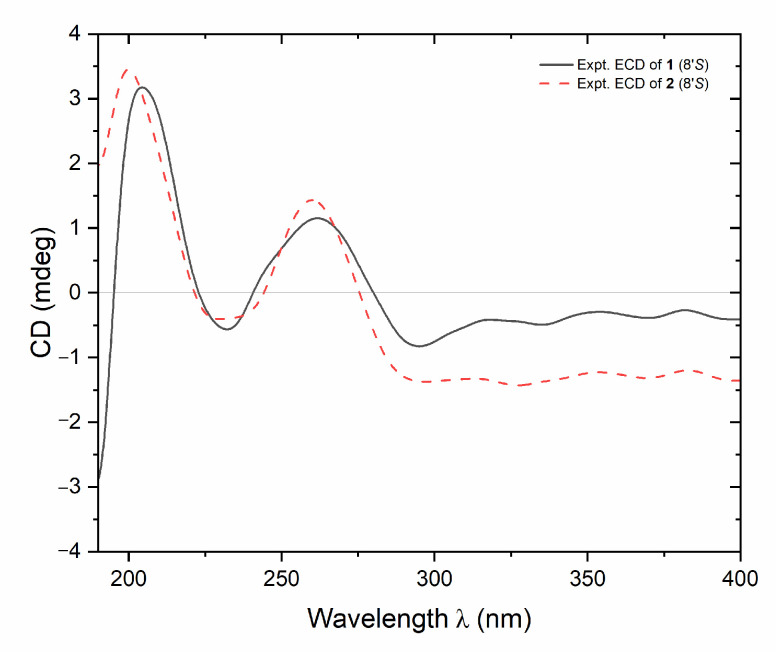
The CD spectra of compounds **1** and **2**.

Dolilabterpenoside A (**1**) presented as a white powder and exhibited positive optical rotation ([α]_D_^25^ +17.5, MeOH). Its molecular formula was determined as C_27_H_42_O_15_ (*m/z* 605.24481 [M–H]^−^; calcd for C_27_H_41_O_15_, 605.24400) by ESI-Q-Orbitrap MS analysis. The IR absorption spectrum manifested characteristic absorption of hydroxyl (3381 cm^−1^), conjugated carboxyl (1693 cm^−1^), olefinic bond (1634 cm^−1^), and oxyglycosidic bond (1075 cm^−1^). The result of HPLC analysis of its acid hydrolysis followed by the derivation of l-cysteine methyl ester hydrochloride and *O*-toluene isothiocyanate suggested the existence of d-glucose in **1** [11]. Combined with two anomeric proton signals at δ_H_ 4.39 (1H, d, *J* = 8.0 Hz, H-1″), 4.85 (1H, d, *J* = 3.5 Hz, H-1‴) shown in its ^1^H NMR spectrum (CD_3_OD, Table 1), the existence of α-d-glucopyranosyl and β-d-glucopyranosyl were verified. Twenty-seven carbon signals were presented in its ^13^C NMR spectrum (CD_3_OD, Table 1). Except the signals assignable to the above two glycosyls, the majority of the remaining fifteen carbon signals were displayed in the range of δ_C_ 16–90, suggesting that it was one of sesquiterpenoid glycosides. Moreover, the ^1^H NMR spectrum indicated the existence of three methyl [δ_H_ 0.98, 1.17, 2.08 [(3H each, all s, H_3_-9′, 10′, 6)] (the methyl with δ_H_ 2.08 is attached to a sp^2^ carbon atom), two methylene {[1.79 (1H, t like, ca. *J* = 13 Hz), 1.96 (1H, dd, *J* = 6.5, 13.5 Hz), H_2_-4′], [1.81 (1H, dd, *J* = 10.5, 13.0 Hz), 2.19 (1H, dd, *J* = 6.5, 13.5 Hz), H_2_-2′]}, one oxygenated methylene [δ_H_ 3.79 (2H, s, H_2_-7′)], one oxygenated methine [δ_H_ 4.27 (1H, tdd, *J* = 6.5, 10.5, 13.0 Hz, H-3′)], one pair of *trans* olefinic protons [δ_H_ 6.51 (1H, d, *J* = 16.0 Hz, H-5), 7.98 (1H, d, *J* = 16.0 Hz, H-4)], and one trisubstituted olefinic bond [δ_H_ 5.76 (1H, s, H-2)] in its aglycone. In the HMBC spectrum, correlations between H_2_-7′ and C-1′, C-5′, and C-8′ were observed, indicating the existence of a five-membered oxygen-containing ring. Furthermore, according to the cross-peaks between H-3′ and H_2_-2 and the finding of H_2_-4 in its ^1^H ^1^H COSY spectrum, as well as the correlations between H-2 and C-1, C-3, C-4, and C-6; H-5 and C-2–C-4, C-1′, and C-8′; H_3_-6 and C-2–C-4; H_2_-7′ and C-9′; H_3_-9′ and C-4′, C-5′, C-7′, and C-8′; H_3_-10′ and C-1′, C-2′, and C-8′; H-1″ and C-3; and H-1‴ and C-6″ observed in its HMBC spectrum (Figure 2), the planar structure of its aglycone was consolidated. Meanwhile, the NOE correlations observed between δ_H_ 2.08 (H_3_-6) and δ_H_ 5.76 (H-2) indicated that Δ2 was in *Z* orientation. Additionally, through the NOE cross-peaks shown between δ_H_ 4.27 (H-3′) and δ_H_ 1.96 (Hα-4′); 2.19 (Hα-2′) and 3.79 (H_2_-7′); δ_H_ 6.51 (H-5) and δ_H_ 1.17 (H_3_-10′), 1.79 (Hβ-4′), and 1.81 (Hβ-2′) (Figure 3), the relative configurations of C-1′, C-3′, C-5′, and C-8′ were elucidated. Compound **1** displayed a positive Cotton effect at 261 nm and a negative one at 231 nm (Figure 4), which was same as that of the reported compound (1′*R*,3′*S*,5′*R*,8′*S*,2*E*,4*E*)-dihydrophaseic acid 3′-*O*-β-d-glucopyranoside [12], suggesting that the absolute configuration of C-8′ was *S*. Thus, the structure of dolilaberpenoside A (**1**) was identified as (1′*R*,3′*S*,5′*R*,8′*S*,2*Z*,4*E*)-dihydrophaseic acid 3′-*O*-α-d-glucopyranosyl(1→6)-*O*-β-d-glucopyranoside.

Dolilabterpenoside B (**2**) presented as a white powder, exhibiting positive optical rotation ([α]_D_^25^ +15.0, MeOH). Its molecular formula, C_27_H_42_O_15_ (*m/z* 605.24512 [M–H]^−^; calcd for C_27_H_41_O_15_, 605.24400) was in accordance with that of compound **1**. The analysis of its ^1^H, ^13^C NMR (CD_3_OD, Table 1), and 2D NMR spectra demonstrated that it shared the same planar structure as **1**. However, their chemical shifts from C-1 to C-6 were significantly different, which may be attributed to the distinct configuration of the olefinic bond. This speculation was supported by the NOE correlations presented between δ_H_ 5.87 (H-2) and δ_H_ 6.64 (H-4) and δ_H_ 2.30 (H_3_-6) and δ_H_ 6.51 (H-5) (Figure 3), suggesting that Δ2 and Δ4 were both in *E* orientation for compound **2**. Finally, the structure of dolilaberpenoside B (**2**) was identified as (1′*R*,3′*S*,5′*R*,8′*S*,2*E*,4*E*)-dihydrophaseic acid 3′-*O*-α-d-glucopyranosyl(1→6)-*O*-β-d-glucopyranoside.

**Figure 5:**
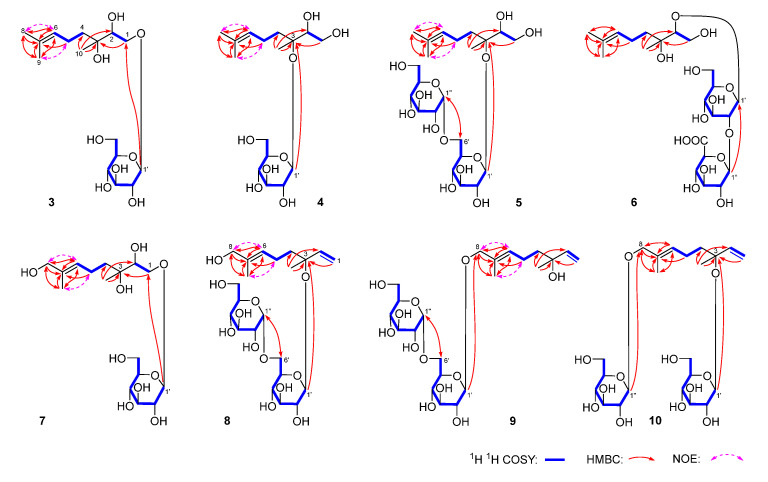
The main ^1^H ^1^H COSY and HMBC correlations between compounds **3**–**10** and NOE correlations between compounds **3**–**5** and **7**–**9**.

Dolilabterpenoside C_1_ (**3**) was obtained as a white powder and exhibited a pseudomolecular ion peak at *m/z* 373.18314 [M + Na]^+^ (calcd for C_16_H_30_O_8_Na, 373.18329) corresponding to the molecular formula, C_16_H_30_O_8_. The existence of d-glucose was affirmed by acid hydrolysis as well as l-cysteine methyl ester hydrochloride and *O*-toluene isothiocyanate derivatization [11]. The ^1^H (CD_3_OD, Table 2), ^13^C NMR (CD_3_OD, Table 3), and HSQC spectra suggested the presence of three methyl [δ_H_ 1.14, 1.62, 1.67 (3H each, all s, H_3_-10, 9, 8)], two methylene [δ_H_ 1.43, 1.59 (1H each, both ddd, *J* = 5.5, 12.5, 12.5 Hz, H_2_-4), 2.08 (2H, m, H_2_-5)], one oxymethylene [δ_H_ 3.52 (1H, dd, *J* = 9.5, 9.5 Hz), 4.19 (1H, br. d, ca. *J* = 10 Hz), H_2_-1], one oxygenated methine [δ_H_ 3.65 (1H, br. d, ca. *J* = 10 Hz, H-2)], and one olefinic proton [δ_H_5.12 (1H, m, H-6)], along with one β-d-glucopyranosyl [δ_H_ 4.33 (1H, d, *J* = 8.0 Hz, H-1′)]. The existence of “–(O)CH_2_–CH(O)–” fragment was clarified by the correlation between δ_H_ 3.52, 4.19 (H_2_-1) and δ_H_ 3.65 (H-2) observed in its ^1^H ^1^H COSY spectrum. Meanwhile, the cross-peaks found between δ_H_ 2.08 (H_2_-5) and δ_H_ 1.43, 1.59 (H_2_-4), and 5.12 (H-6) suggested the presence of “–CH_2_–CH_2_–CH–” moiety. Furthermore, the planar structure of it was elucidated to be 3,7-dimethyl-6-octene-1,2,3-triol through the HMBC correlations observed between H_3_-8 and C-6, C-7, and C-9; H_3_-9 and C-6–C-8; and H_3_-10 and C-2–C-4 (Figure 5). The signals of H_3_-8 and H_3_-9 were assigned through the NOE corrections found between δ_H_ 1.67 (H_3_-8) and δ_H_ 5.12 (H-6) and δ_H_ 1.62 (H_3_-9) and δ_H_ 2.08 (H-5). In addition, the HMBC correlation discovered between δ_H_ 4.33 (H-1′) and δ_C_ 72.4 (C-1) suggested that the substitution position of β-d-glucopyranosyl was at C-1. Thus, the planar structure of dolilaberpenoside C_1_ (**3**) was concluded. It was a monoterpenoid glycoside and consistent with that of 1,2-dihydroxylinalool-1-*O*-(1-β-d-glucopyranoside) [13]. To determine its absolute configuration, we initially summarized the optical rotation of four isomers of 3,7-dimethyl-6-octene-1,2,3-triol (2*R*,3*S*: +5.0; 2*R*,3*R*: +1.5; 2*S*,3*S*: –1.5; 2*S*,3*R*: –6.7, in CHCl_3_) [14,15]. It was discovered that the optical rotation was only related to the absolute configuration of C-2 (the 2*R* configuration was positive and the 2*S* configuration was negative). Secondly, compound **3** was hydrolyzed by β-glucosidase to obtain its aglycone **3a**, which showed positive optical rotation ([α]_D_^25^ +6.3 (*conc* 0.16, CHCl_3_), indicating 2*R* configuration. By comparing its ^1^H and ^13^C NMR data with (2*R*,3*R*)-3,7-dimethyl-6octene-1,2,3-triol and (2*R*,3*S*)-3,7-dimethyl-6octene-1,2,3-triol [8,15], it was found to be consistent with those of the latter. Consequently, the structure of dolilaberpenoside C_1_ (**3**) was identified as (2*R*,3*S*)-3,7-dimethyl-6-octene-1,2,3-triol 1-*O*-β-d-glucopyranoside.

Similarly to compound **3**, dolilabterpenoside C_2_ (**4**) exhibited negative optical rotation ([α]_D_^25^ –6.0, MeOH). ESI-Q-Orbitrap MS analysis demonstrated that its molecular formula, C_16_H_30_O_8_ (*m/z* 349.18643 [M–H]^−^; calcd for C_16_H_29_O_8_, 349.18569) was the same as that of **3**. The presence of β-d-glucopyranosyl was confirmed by employing the same method as **3**. The ^1^H (CD_3_OD, Table 2) and ^13^C NMR (CD_3_OD, Table 3), as well as the ^1^H ^1^H COSY, HSQC, and HMBC spectra suggested that the planar structure of its aglycone was also identical to that of **3**. Compound **4** was hydrolyzed by β-glucosidase to yield **3a** as well. Eventually, the linkage position of β-d-glucopyranosyl was determined to be C-3 position based on the HMBC correlation observed between δ_H_ 4.51 (H-1′) and δ_C_ 81.9 (C-3) (Figure 5). Thus, the structure of dolilabterpenoside C_2_ (**4**) was elucidated as (2*R*,3*S*)-3,7-dimethyl-6-octene-1,2,3-triol 3-*O*-β-d-glucopyranoside.

The molecular formula of dolilabterpenoside C_3_ (**5**) was disclosed to be C_22_H_40_O_13_ (*m/z* 557.24426 [M + COOH]^−^; calcd for C_23_H_41_O_15_, 557.24400) by ESI-Q-Orbitrap MS analysis. Comparison of the ^1^H (CD_3_OD, Table 2) and ^13^C NMR (CD_3_OD, Table 3) spectra with those of compound **4**, suggested that its aglycone was also (2*R*,3*S*)-3,7-dimethyl-6-octene-1,2,3-triol. Twenty-two carbon signals were presented in the ^13^C NMR spectrum of compound **5**, inferring the containment of two hexoses. However, only d-glucose was detected in the derivatives after acid hydrolysis and derivatization [11]. Combined with the coupling constants of the two anomeric proton (*J* = 3.6 Hz and *J* = 7.8 Hz), the existence of α-d-glucopyranosyl and β-d-glucopyranosyl was consolidated. The NMR data of the two glycosyls were assigned by integrating the proton–proton correlations provided in the ^1^H ^1^H COSY spectrum (Figure 5) and the HSQC correlation observed between proton and carbon. Finally, the structure of **5** was determined as (2*R*,3*S*)-3,7-dimethyl-6-octene-1,2,3-triol 3-*O*-α-d-glucopyranosyl(1→6)-*O*-β-d-glucopyranoside through the HMBC correlations found between δ_H_ 4.56 (H-1′) and δ_C_ 82.2 (C-3) and δ_H_ 4.83 (H-1″) and δ_C_ 67.7 (C-6′).

Dolilabterpenoside D (**6**) was a white powder with negative optical rotation ([α]_D_^25^ –22.0, MeOH). Its molecular formula was determined to be C_22_H_38_O_14_ (*m/z* 525.21832 [M–H]^−^; calcd for C_22_H_37_O_14_, 525.21778) by ESI-Q-Orbitrap MS analysis. Its acid hydrolysis and derivatization results indicated the presence of d-glucose and d-glucuronic acid [11]. Its ^1^H NMR spectrum (CD_3_OD, Table 4) displayed two anomeric proton signals at δ_H_ 4.48 (1H, d, *J* = 7.5 Hz, H-1′) and 4.70 (1H, d, *J* = 8.0 Hz, H-1″), suggesting that they were, respectively, β-d-glucopyranose and β-d-glucuronic acid. Twenty-two signals were presented in its ^13^C NMR spectrum (CD_3_OD, Table 3). Excluding the signals belong to two glycosyls, the remaining ten indicated that **6** was also one monoterpenoid glycoside. Its ^1^H NMR spectrum manifested signals attributable to three methyl at δ_H_ 1.10, 1.62, and 1.67 (3H each, all s, H_3_-10, 9, 8); two methylene at δ_H_ 1.48 (2H, m, H_2_-4) and 2.10 (2H, m, H_2_-5); two oxymethylene at δ_H_ 3.55 and 3.63 (1H each, both m, overlapped, H_2_-1); one oxygenated methine at δ_H_ 3.51 (1H, t like, ca. *J* = 8 Hz, H-2); and one olefinic proton at δ_H_ 5.10 (1H, m, H-6). Additionally, the existence of fragments ‘‘–(O)CH_2_–CH(O)–’’ and ‘‘–CH_2_–CH_2_–CH–’’ was clarified through the cross-peaks displayed in its ^1^H ^1^H COSY spectrum (Figure 5). Moreover, the planar structure of the aglycone was identified as 3,7-dimethyl-6-octene-1,2,3-triol according to the HMBC correlations between H_3_-8 and C-6, C-7, and C-9; H_3_-9 and C-6–C-8; and H_3_-10 and C-2–C-4 (Figure 5). Furthermore, the connection position of glycosyl with glycosyl and glycosyl with aglycone were confirmed by the long-range correlations between δ_H_ 4.48 (H-1′) and δ_C_ 91.8 (C-2) and δ_H_ 4.70 (H-1″) and δ_C_ 104.5 (C-1′) that appeared in its HMBC spectrum. Subsequently, compound **6** was hydrolyzed by β-glucuronidase to generate its aglycone, **6a**. **6a** exhibited negative optical rotation ([α]_D_^25^ –6.6, in CHCl_3_), suggesting that the absolute configuration of C-2 was *S* [8,15]. Eventually, by comparing the ^1^H and ^13^C NMR data (Table 3) of **6a** with those of (2*S*,3*S*)-3,7-dimethyl-6-octene-1,2,3-triol and (2*S*,3*R*)-3,7-dimethyl-6-octene-1,2,3-triol [8,15], it was discovered to be consistent with the former. Therefore, the structure of dolilaberpenoside D (**6**) was identified as (2*S*,3*S*)-3,7-dimethyl-6-octene-1,2,3-triol 2-*O*-β-d-glucopyranuronosyl(1→2)-*O*-β-d-glucopyranoside.

Dolilabterpenoside E (**7**) showed a pseudomolecular ion peak at *m/z* 389.17807 [M + Na]^+^ (calcd for C_16_H_30_O_9_Na, 389.17820), corresponding to the molecular formula, C_16_H_30_O_9_. The ^1^H (Table 4) and ^13^C NMR data (Table 3) were highly similar to those of compound **3**, except for the disappearance of one methyl and the appearance of one hydroxymethyl [δ_H_ 3.91 (2H, s, H_2_-8); δ_C_ 69.0 (C-8)]. According to the correlations shown in its ^1^H ^1^H COSY (Figure 5) spectrum, the three moieties, indicated by bold lines, were established. Moreover, the planar structure of dolilabterpenoside E (**7**) was ascertained by the long-range correlations observed between H_2_-8 and C-6, C-7, C-9; H_3_-9 and C-6–C-8; H_3_-10 and C-2–C-4; and H-1′ and C-1 (Figure 5) in its HMBC spectrum. Its optical rotation ([α]_D_^25^ –29.6, MeOH) was similar to that of compound **3**. Additionally, the chemical shift of C-1–C-5 that appeared was also nearly the same as that of **3**, suggesting that the absolute configuration of **7** was also 2*R*,3*S*. The NOE cross-peaks between δ_H_ 3.91 (H_2_-8) and δ_H_ 5.41 (H-6) and δ_H_ 1.67 (H_3_-9) and δ_H_ 2.15 (H_2_-5) (Figure 5) observed in its NOESY spectrum, prompted the conclusion that Δ6 had an *E* configuration. Consequently, the structure of dolilabterpenoside E (**7**) was identified as (2*R*,3*S*,6*E*)-3,7-dimethyl-6-octene-1,2,3,8-tetraol 1-*O*-β-d-glucopyranoside.

Dolilabterpenoside F_1_ (**8**) was obtained as a white powder with positive optical rotation ([α]_D_^25^ +30.0, MeOH). The molecular formula of it was determined as C_22_H_38_O_12_ (*m/z* 539.23444 [M + COOH]^−^; calcd for C_23_H_39_O_14_, 539.23343) by ESI-Q-Orbitrap MS analysis. Compound **8** was initially hydrolyzed with HCl and subsequently derivatized with l-cysteine methyl ester hydrochloride and *O*-toluene isothiocyanate in sequence to obtain derivative. Comparison *t*_R_ (19.0 min) of the obtain derivative with those of sugar standard samples’ derivatives indicated the presence of d-glucose in **8** [11]. Its ^1^H (CD_3_OD, Table 5) spectrum displayed the anomeric proton signals at δ_H_ 4.39 (1H, d, *J* = 8.4 Hz, H-1′) and 4.81 (1H, d, *J* = 3.6 Hz, H-1″), signifying the presence of β-d-glucopyranosyl and α-d-glucopyranosyl, respectively. Meanwhile, the ^1^H NMR spectrum of it showed the signals assignable to two methyl at δ_H_ 1.40 and 1.63 (3H each, both s, H_3_-10, 9); two methylene at δ_H_ 1.64 (2H, m, H_2_-4) and 2.10 (2H, m, H_2_-5); one oxymethylene at δ_H_ 3.90 (2H, s, H_2_-8); one trisubstituted olefinic bond at δ_H_ 5.38 (1H, m, H-6); and one monosubstituted olefinic bond at [5.23 (1H, dd, *J* = 1.2, 10.8 Hz), 5.26 (1H, dd, *J* = 1.2, 18.0 Hz), H_2_-1] 5.94 (1H, dd, *J* = 10.8, 18.0 Hz, H-2). Furthermore, the above-mentioned moieties were concatenated together through the HMBC correlations observed between H_2_-8 and C-6, C-7, and C-9; H_3_-9 and C-6–C-8; H_3_-10 and C-2–C-4; H-1′ and C-3; and H-1″ and C-6′ (Figure 5). NOE cross-peaks between δ_H_ 3.90 (H_2_-8) and δ_H_ 5.38 (H-6) and δ_H_ 1.63 (H_3_-9) and δ_H_ 2.10 (H_2_-5) were perceived in its NOESY spectrum (Figure 5), indicating that Δ6 was *E* configuration. Dolilabterpenoside F_1_ (**8**) was hydrolyzed by β-glucosidase to obtain **8a**. Both the optical rotation ([α]_D_^25^ +8.1 (in CHCl_3_) and ^1^H NMR data of **8a** were in accordance with those of (*S*)-(+)-3,7-dimethylocta-1,6-diene-3,8-diol [[α]_D_^25^ +17.0 (in CHCl_3_)] [16]. Thus, the structure of dolilabterpenoside F_1_ (**8**) was elucidated to be (3*S*,6*E*)-3,7-dimethylocta-1,6-diene-3,8-diol 3-*O*-α-d-glucopyranosyl(1→6)-*O*-β-d-glucopyranoside.

The ESI-Q-Orbitrap MS analysis suggested the molecular formula C_22_H_38_O_12_ (*m/z* 517.22522 [M + Na]^+^; calcd for C_22_H_38_O_12_Na, 517.22555) of dolilabterpenoside F_2_ (**9**) was identical to that of compound **8**. Comparison of its ^1^H (CD_3_OD, Table 5) and ^13^C NMR (CD_3_OD, Table 3) spectra with those of **8** confirmed the presence of one α-d-glucopyranosyl(1→6)-*O*-β-d-glucopyranosyl [δ_H_ 4.27 (1H, d *J* = 7.8 Hz, H-1′), 4.83 (1H, d, *J* = 3.6 Hz, H-1″) moiety. After hydrolyzing with β-glucosidase, its aglycone, (3*S*,6*E*)-3,7-dimethylocta-1,6-diene-3,8-diol (**8a**) was produced. Moreover, the position of glycosylation was determined by the HMBC correlations observed between H-1′ and C-8 (Figure 5). Eventually, dolilabterpenoside F_2_ (**9**) was identified as (3*S*,6*E*)-3,7-dimethylocta-1,6-diene-3,8-diol 8-*O*-α-d-glucopyranosyl(1→6)-*O*-β-d-glucopyranoside through the HMBC correlation between δ_H_ 4.27 (H-1′) and δ_C_ 76.2 (C-8) (Figure 5).

Dolilabterpenoside F_3_ (**10**) was obtained as a white powder with negative optical rotation ([α]_D_^25^ –52.0, MeOH). Its molecular formula was revealed to be C_22_H_38_O_12_ (*m/z* 517.22406 [M + Na]^+^; calcd for C_22_H_38_O_12_Na, 517.22555) by ESI-Q-Orbitrap MS analysis, which was consistent with that of compounds **8** and **9**. By using similar reaction and detection methods as used for compound **8**, the presence of d-glucose was verified [11]. According to the coupling constant of anomeric protons at δ_H_ 4.26 (1H, d, *J* = 7.5 Hz, H-1″) and 4.37 (1H, d, *J* = 7.5 Hz, H-1′) (CD_3_OD, Table 5), the existence of two β-d-glucopyranoyl groups were determined. Furthermore, its aglycone, (3*S*,6*E*)-3,7-dimethylocta-1,6-diene-3,8-diol (**8a**), was obtained by hydrolyzation with β-glucosidase. Finally, the substitution position of the two β-d-glucopyranoyl groups was clarified by the HMBC correlations observed between H-1′ and C-3 and H-1″ and C-8. Consequently, its structure was clarified as (3*S*,6*E*)-3,7-dimethylocta-1,6-diene-3,8-diol 8-*O*-β-d-glucopyranosyl-3-*O*-β-d-glucopyranoside.

In addition, the structures of known compounds **11**–**14** were identified by comparing their ^1^H and ^13^C NMR data with those reported in references.

The results of the research literature indicate that from ancient medical records to modern clinical and pharmacological studies, as well as daily applications, *D. lablab* seeds have been extensively utilized. Their efficacy is primarily associated with regulating spleen–stomach disharmony and alleviating chronic colitis and diarrhea [17,18]. In vitro anti-inflammatory study suggested that its methanol extract had significant anti-inflammatory activity [5]. However, despite our previously finding suggested that the aromatic compounds in *D. lablab* seeds had anti-inflammatory activity [4], the active components for anti-inflammation are not very clear. To further explore its anti-inflammatory components, a comprehensive phytochemical investigation was initially conducted by using various chromatographic and spectrometric technologies, as well as chemical reactions. As a result, ten previously unreported terpenoid glycosides, namely dolilabterpenosides A, B, C_1_–C_3_, D, E, and F_1_–F_3_ (**1**–**10**), along with four known analogues (**11**–**14**) were isolated and identified. All of the known compounds were firstly identified from *Dolichos* genus, and **13** as well as **14** were gained from the Leguminosae family for the first time. Moreover, the ^13^C NMR data of compound **11** was reported for the first time. The study partially clarified the material basis of *D. lablab* seeds and complemented previous work.

### 2.2. Biological Research Results and Discussion

**Figure 6:**
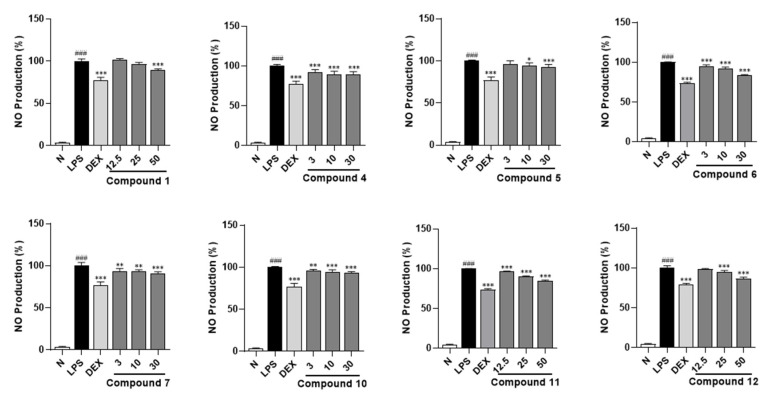
A dose-dependent inhibitory effects of compounds **1**, **4**–**7**, and **10**–**12** on NO production in RAW264.7 cells. N—normal group; LPS—LPS-stimulated group; DEX—dexamethasone, a positive group. Nitrite relative concentration (NRC): percentage of control group (set as 100%). Values represent the mean ± SD of six determinations. * *p* < 0.05; ** *p* < 0.01; *** *p* < 0.001 (differences between compound-treated group and LPS-stimulated group). ^###^ *p* < 0.001 (differences between LPS-stimulated group and normal group). Final concentration was 50 μM for compounds **1**, **11**, and **12**; 30 μM for compounds **4**–**7** and **10**; 1.5 μg/mL for DEX; and 0.5 μg/mL for LPS.

Moreover, an NO production inhibitory effect experiment was conducted for all the obtained terpenoid glycosides **11**–**14** using an LPS-stimulated RAW264.7 cell model at safe treatment concentrations (50 μm for compounds **1**, **3**, **8**, **9**, and **11**–**14**; 30 μm for compounds **2**, **4**, **5**–**7** and **10**), which were determined by the MTT assay (Figure S89). The results demonstrated that compounds **1**, **11**, and **12** at 50 μm and **4**–**7** and **10** at 30 μm could significantly inhibit the increase in NO level stimulated by LPS in RAW264.7 cells (Table 6). This suggested that compounds **1**, **4**–**7**, and **10**–**12** possessed potential anti-inflammatory activity. Meanwhile, all of them exhibited dose-dependent activity (Figure 6).

**Figure 7:**
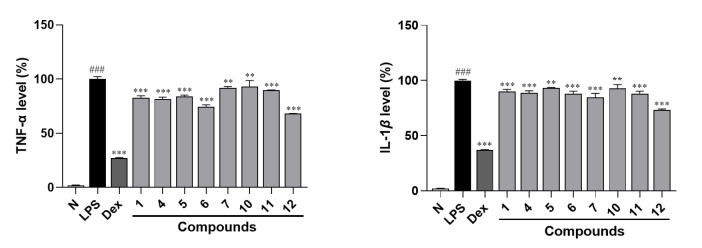
ELISA assay of TNF-α and IL-Iβ in the supernatant of RAW264.7 cells. N—normal group; LPS—LPS-stimulated group; DEX—dexamethasone, a positive group. Nitrite relative concentration (NRC): percentage of control group (set as 100%). Values represent the mean ± SD of three determinations. ** *p* < 0.01; *** *p* < 0.001 (differences between compound-treated group and LPS-stimulated group). ^###^ *p* < 0.001 (differences between LPS-stimulated group and normal group). Final concentration was 50 μM for compounds **1**, **11**, and **12**; 30 μM for compounds **4**–**7** and **10**; 1.5 μg/mL for DEX; and 0.5 μg/mL for LPS.

Furthermore, the ELISA experimental results indicated that the levels of TNF-α and IL-Iβ in RAW264.7 cells’ supernatant were significantly elevated after stimulation by LPS. However, each administration group (compounds **1**, **4**–**7**, and **10**–**12**) could suppress the increase in TNF-α and IL-Iβ compared with LPS-stimulated group (Figure 7), verifying that compounds **1**, **4**–**7**, and **10**–**12** exhibit anti-inflammatory activity.

As is well known, sustained inflammatory response will promote the release of NO and even inflammation-related diseases [19]. Therefore, the detection of anti-inflammatory components from natural products is important for treating inflammatory diseases. In the present study, the LPS-stimulated RAW264.7 cells were used as the activity screening model, and the inhibitory effect on NO release was used as the evaluation index to investigate the potential anti-inflammatory effect of compounds **1**–**14**. The results of bioassay showed that compounds **1**, **4**–**7**, and **10**–**12** exerted significant inhibitory activity on NO release in RAW264.7 cells in a dose-dependent manner. Summarization of the structure-activity relationships (SARs) suggested that the substitution position of glycosyl could influence their activity, with 3-glycosyl substitution being more effective than 1-glycosyl substitution (**4** vs. **3**).

Meanwhile, it was found that the inhibitory effect decreased with the increase in substituted glycosyl (**4** vs. **5**; **12** vs. **1**). Meanwhile, the expression of cytokines TNF-α and IL-1β can serve as clinical indexes to judge the degree of inflammation and the therapeutic effect of drugs. In this study, compounds **1**, **4**–**7**, and **10**–**12** were demonstrated to inhibit the increase in TNF-α and IL-Iβ levels in the supernatant of RAW264.7 cells stimulated by LPS. All the above results indicated that terpenoid glycosides might be one of main material basis of *D. lablab* seeds for improving inflammation, which will provide scientific evidence for the use of *D. lablab* seeds in daily life.

## 3. Experimental

### 3.1. Materials and Methods for Phytochemistry Research

#### 3.1.1. General Experimental Procedures

Column chromatography (CC) isolation was conducted by using Macroporous resin D101 (Haiguang Chemical, Tianjin, China), silica gel (48–75 μm, Qingdao Haiyang Chemical, Qingdao, China), and YMC*Gel ODS-A-HG (S-50 μm, AAG12S50, YMC, Kyoto, Japan). For analysis, HPLC was carried out using Cosmosil 5C18-MS-II and PBr columns (4.6 mm i.d. × 250 mm, 5 µm, Nakalai Tesque, Kyoto, Japan); for purification, the same columns (20 mm i.d. × 250 mm, 5 µm, Nakalai Tesque) were applied.

A Waters e2695 equipped with a 2998 PDA detector (Waters Corporation, MA, USA) was used for analytical HPLC, while a Shimadzu LC-8A with an SPD-20A detector (Shimadzu Corporation, Kyoto, Japan) was employed for preparative HPLC. Bruker Ascend 600/500 MHz spectrometers (Bruker, MA, USA) were used to measure NMR spectra. A Thermo ESI-Q-Orbitrap MS (Thermo Fisher Scientific, MA, USA) connected to an UltiMate 3000 UHPLC (Thermo Fisher Scientific, MA, USA) was applied to obtain mass spectra. A Rudolph Autopol V (Rudolph Technologies, Geretsried, Germany), a Varian Cary 50 (Agilent Technologies, Inc., Santa Clara, CA, USA), and a Varian 640-IR FT-IR (Agilent Technologies, Inc., CA, USA) were used to acquire optical rotations, UV spectra, and IR spectra, respectively.

#### 3.1.2. Plant Material

On 27 September 2021, *D. lablab* seeds from Dabie Mountain, Anhui, were purchased from Tongrentang (Beijing, China) and identified by Prof. Lin Ma (Tianjin Univ. of TCM, No. 2021092702) [4].

#### 3.1.3. Extraction and Isolation

As we previously reported [4], *D. lablab* seeds (25.0 kg) were extracted under reflux with 70% EtOH three times (3 h, 3 h, and 2 h, respectively). The extract was concentrated and partitioned with EtOAc-H_2_O (1:1, *v*/*v*) to gain H_2_O extract (DLSS, 1.7 kg). DLSS was subjected to a D101 resin CC eluted with H_2_O and 95% EtOH sequentially to give 95% EtOH eluate (DLSH, 96.2 g).

DLSH (90.0 g) was fractionated by silica gel CC [CH_2_Cl_2_-MeOH (100:1 → 100:3 → 100:7 → 8:1 → 7:1 → 5:1 → 4:1 → 3:1 → 2:1 → 0:1, *v*/*v*) to yield DLSH 1–DLSH 16. DLSH 8 (6.3 g) was loaded onto ODS CC [MeOH-H_2_O (20:80 → 30:70 → 40:50 → 50:50 → 60:40 → 70:30 → 100:0, *v*/*v*)] to gain DLSH 8-1–DLSH 8-12. DLSH 8-2 (1288.5 mg) was subjected to pHPLC [MeOH-1% HAc (15:85, *v*/*v*), Cosmosil 5C_18_-MS-II column] to produce DLSH 8-2-1–DLSH 8-2-7. DLSH 8-2-5 (19.5 mg) and DLSH 8-2-7 (106.7 mg) were purified by pHPLC [CH_3_CN-1% HAc (8:92, *v*/*v*), Cosmosil 5C_18_-MS-II column] to yield gA_8_-2-*O*-β-d-glucopyranoside (**11**, 16.6 mg, *t*_R_ 24.0 min) and (1′*R*,3′*S*,5′*R*,8′*S*,2*Z*,4*E*)-dihydrophaseic acid (**12**, 42.2 mg, *t*_R_ 27.4 min), respectively. DLSH 8-4 (771.8 mg) was prepared by pHPLC [CH_3_CN-1% HAc (9:91, *v*/*v*), Cosmosil 5C_18_-MS-II column] to gain DLSH 8-4-1–DLSH 8-4-7. DLSH 8-4-4 (32.1 mg) was purified by pHPLC [MeOH-1% HAc (20:80, *v*/*v*), Cosmosil 5C_18_-MS-II column] to give (3*S*)-6,7-dihydroxy-dihydrolinalool-3-*O*-β-glucopyranoside (**14**, 2.9 mg, *t*_R_ 43.5 min). DLSH 8-4-7 (69.2 mg) was subjected to pHPLC [MeOH-1% HAc (23:77, *v*/*v*), Cosmosil 5C_18_-MS-II column] to obtain 3,7-dimethyl-oct-1-en-3,6,7-triol-6-*O*-β-d-glucopyranoside (**13**, 18.2 mg, *t*_R_ 42.1 min). DLSH 8-7 (758.7 mg) was fractionated by [CH_3_CN-1% HAc (17:83, *v*/*v*), Cosmosil 5C_18_-MS-II column] to gain DLSH 8-7-1–DLSH 8-7-5. Among them, DLSH 8-7-5 (138.9 mg) was identified as dolilabterpenoside C_1_ (**3**, 138.9 mg, *t*_R_ 49.0 min). DLSH 8-7-3 (26.0 mg) was separated by pHPLC [MeOH-1% HAc (34:66, *v*/*v*), Cosmosil 5C_18_-MS-II column] to yield dolilabterpenoside C_2_ (**4**, 7.0 mg, *t*_R_ 38.9 min). DLSH10 (4.9 g) was loaded onto ODS CC [MeOH-H_2_O (20:80 → 30:70 → 40:50 → 50:50 → 60:40 → 100:0, *v*/*v*)] to obtain DLSH 10-1–DLSH 10-10. DLSH 10-2 (334.2 mg) was subjected to pHPLC [MeOH-1% HAc (10:90, *v*/*v*), Cosmosil 5C_18_-MS-II column] to yield dolilabterpenoside E (**7**, 9.8 mg, *t*_R_ 51.1 min). DLSH11 (6.7 g) was loaded onto ODS CC [MeOH-H_2_O (20:80 → 30:70 → 40:50 → 50:50 → 60:40 → 100:0, *v*/*v*)] to gain DLSH 11-1–DLSH 11-11. DLSH 11-4 (1047.8 mg) was separated by pHPLC [CH_3_CN-1% HAc (12:88, *v*/*v*), Cosmosil 5C_18_-MS-II column] to produce DLSH 11-4-1–DLSH 11-4-7. DLSH 11-4-4 (64.8 mg) was identified as dolilabterpenoside F_3_ (**10**, 64.8 mg, *t*_R_ 38.4 min). DLSH 11-4-5 (20.0 mg) was prepared with pHPLC [MeOH-1% HAc (30:70, *v*/*v*), Cosmosil 5C_18_-MS-II column] to yield dolilabterpenoside F_2_ (**9**, 9.0 mg, *t*_R_ 57.0 min). DLSH 11-4-6 (17.5 mg) was purified by pHPLC [MeOH-1% HAc (35:65, *v*/*v*), Cosmosil 5C_18_-MS-II column] to obtain dolilabterpenoside F_1_ (**8**, 10.7 mg, *t*_R_ 45.0 min). DLSH 12 (9.2 g) was fractionated by ODS CC [MeOH-H_2_O (20:80 → 30:70 → 40:50 → 50:50 → 60:40 → 100:0, *v*/*v*)] to gain DLSH 12-1–DLSH 12-15. DLSH 12-3 (641.2 mg) was prepared with pHPLC [CH_3_CN-1% HAc (13:87, *v*/*v*), Cosmosil 5C_18_-MS-II column] to produce DLSH 12-3-1–DLSH 12-3-4. DLSH 12-3-3 (66.1 mg) was further purified by pHPLC [CH_3_CN-1% HAc (13:87, *v*/*v*), Cosmosil 5C_18_-MS-II column] to yield dolilabterpenoside A (**1**, 30.1 mg, *t*_R_ 35.0 min). DLSH 12-3-4 (24.8 mg) was subjected to pHPLC [CH_3_CN-1% HAc (12:88, *v*/*v*), Cosmosil PBr column], and dolilabterpenoside B (**2**, 4.4 mg, *t*_R_ 29.3 min) was obtained. DLSH 12-5 (252.1 mg) was separated by pHPLC [CH_3_CN-1% HAc (13:87, *v*/*v*), Cosmosil 5C_18_-MS-II column] to gain DLSH 12-5-1–DLSH 12-5-6. DLSH 12-5-5 (15.6 mg) was purified by pHPLC [MeOH-1% HAc (25:75, *v*/*v*), Cosmosil 5C_18_-MS-II column] to yield dolilabterpenoside C_3_ (**5**, 4.7 mg, *t*_R_ 67.5 min). DLSH 12-6 (357.1 mg) was separated by pHPLC [CH_3_CN-1% HAc (18:82, *v*/*v*), Cosmosil 5C_18_-MS-II column] to obtain DLSH 12-6-1–DLSH 12-6-6. DLSH 12-6-4 (19.8 mg) was further prepared by pHPLC [MeOH-1% HAc (35:65, *v*/*v*), Cosmosil 5C_18_-MS-II column] to produce dolilabterpenoside D (**6**, 12.7 mg, *t*_R_ 40.6 min).

#### 3.1.4. Spectral Data of **1**–**14**

The detail spectral data of **1**–**14** are provided in the Supporting Information.

#### 3.1.5. Acid Hydrolysis of Compounds **1**, **3**–**6**, **8** and **10**

By referring to the literature [11], compounds **1**, **3**–**6**, **8**, and **10** (each 2.5 mg) were hydrolyzed with 2 M HCl and derivatized by l-cysteine methyl ester hydrochloride and *O*-toluene isothiocyanate sequentially. The obtained derivatives were analyzed by employing the same HPLC analysis condition as reported in the literature [11]. Subsequently, through comparison of their retention times with that of the authentic sample, d-glucuronic (*t*_R_: 20.0 min) was determined in compound **6** and d-glucose (*t*_R_: 19.0 min) was clarified in compounds **1**, **3**–**6**, **8**, and **10**.

#### 3.1.6. Enzymatic Hydrolysis Reactions of Compounds **3**, **4**, **6**, and **8**–**10**

Compounds **3**, **4**, and **8**–**10** (each 5.0 mg), along with of β-glucosidase (10.0 mg) (Source Leaf Company, Lot F091S205976, Tokyo, Japan), were dissolved in 1.0 mL of H_2_O, and reacted at 37 °C for 12 h, respectively. The reaction solution was extracted with EtOAc to obtain aglycone. Then, the aglycones **3a** of compounds **3** and **4** (2.8 mg from **3**; 2.5 mg from **4**, respectively), as well as **8a** of compounds **8**–**10** (1.8 mg from **8**; 1.5 mg from **9**; 1.6 mg from **10**, respectively), were produced. Compound **6** (3.4 mg) was dissolved in 200 μL KH_2_PO_4_/NaOH (pH = 4.99) buffer, then 100 μL β-glucuronidase (Sigma company, Lot SLCH4963) was added and reacted for 5 h at 37 °C. The reaction solution was extracted with ethyl acetate to obtain aglycone **6a** (1.5 mg).

The detail spectral data of **3a**, **6a**, and **8a** are provided in the Supporting Information.

### 3.2. Experimental Procedures for Bioassay

#### 3.2.1. Reagents

3-(4,5-Dimethyl-2-thiazolyl)-2,5-diphenyl-2-*H*-tetrazolium bromide (MTT), LPS, and dexamethasone (DEX) were sourced from Sigma-Aldrich (St. Louis, MO, USA). The NO kit was purchased from Shanghai Biyuntian Biotechnology Co., Ltd. (Shanghai, China). The ELISA test kit was obtained from Shanghai Jianglai Biotechnology (Shanghai, China). Dulbecco’s modified Eagle’s medium (DMEM), and fetal bovine serum (FBS) were acquired from Biological Industries (Beit HaEmek, Israel).

#### 3.2.2. Cell Culture

RAW264.7 cells were grown in DMEM from Biological Industries (Israel) and were supplemented with 10% (*v*/*v*) FBS from the same company and 100 U/mL of penicillin and 100 μg/mL of streptomycin from Sigma-Aldrich (USA). The cells were kept in a chamber with 5% CO_2_ at 37 °C with maintained humidity. Once the cell confluency reached 80–90%, the cells were passaged.

#### 3.2.3. MTT Assay

In order to determine the safe concentrations for cell experiments, the MTT analyses of compounds **1**–**14** on RAW264.7 cells were carried out using the method that has been reported by us previously [4]. In brief, cells were plated into 96-well plates at a density of 1 × 10^5^ cells/mL and incubated until they achieved 90% confluence. Compounds were prepared as 100 mM stock solutions in DMSO and then diluted with serum-free medium. Cells were exposed to different concentrations of compounds **1**–**14**. Six replicates of 100 μL of each compound solution were added to the respective wells. The non-treated cells (normal group) were maintained in serum-free medium to compare growth inhibition. Afterward, the cells were further incubated for 18 h at 37 °C in an environment with 5% CO_2_. Subsequently, 100 μL of a 500 μg/mL MTT solution was added to all wells and incubated for 4 h. Then, 100 μL of DMSO was added, and the plates were shaken for 2 min. The absorbance was measured at 490 nm using a BioTek Cytation five-cell imaging multi-mode reader from Bio Tek Instruments, Inc., Winooski, VT, USA. The viability of cells in each group was presented as a percentage compared to the normal group.

#### 3.2.4. Analysis of NO Levels in LPS-Induced RAW264.7 Cells

According to what we have reported, the NO production inhibitory assay was conducted [4]. Cells were plated onto 96-well plates at a density of 1 × 10^6^ cells/mL and incubated until reaching 90% confluence. Four groups, namely the normal, control, positive control, and administration groups, were, respectively, treated with serum-free medium, 0.5 μg/mL lipopolysaccharide (LPS), a combination of 0.5 μg/mL LPS and 1.5 μg/mL dexamethasone (DEX), and 0.5 μg/mL LPS along with non-cytotoxic compounds. After an 18 h incubation period, the nitric oxide (NO) content in the cells was determined to be at 540 nm. This measurement was carried out using the Griess assay provided by Beyotime Biotechnology (Shanghai, China), following the detailed instructions from the manufacturer.

#### 3.2.5. ELISA Analysis

An ELISA assay was carried out on the active compounds discovered in Section 3.2.2. The levels of TNF-α and IL-1β in the culture supernatants of RAW264.7 cells were measured using ELISA kits from Jianglai Bio (Shanghai, China), following the manufacturer’s guidelines. Specifically, cells were seeded in 96-well plates at a density of 1 × 10^6^ cells/mL and incubated until they reached 90% confluence. Subsequently, they were treated as previously described [4]. After 18 h, the plates were centrifuged at 4 °C and 3000 rpm for 20 min to obtain the supernatants. With the ELISA kit from Jianglai Bio (Shanghai, China), standards and samples were added to the wells, and then 100 μL of horseradish peroxidase (HRP) was immediately added. After incubation at 37 °C for 1 h, the liquid in the wells was discarded. The plates were then patted dry and washed five times with a washing solution, with each wash lasting 1 min. Next, the substrate solution was added, and the plates were incubated in the dark at 37 °C for 15 min. After that, 50 μL of stop solution was added. The optical density (OD) was measured at 450 nm within 15 min.

#### 3.2.6. Statistical Analysis

Data were presented as the mean ± SD. Significant differences among groups were identified using one-way ANOVA with Dunnett’s multiple comparisons test. Data were regarded as significant when * *p* < 0.05, ** *p* < 0.01, and *** *p* < 0.001. Data analyses were performed using GraphPad Prism 8.0 (GraphPad Software, Inc., La Jolla, CA, USA).

## 4. Conclusions

In the course of investigating anti-inflammatory constituents from *D. lablab* seeds, we successfully isolated and characterized ten novel terpenoid glycosides (**1**–**10**) along with four known analogues (**11**–**14**), the latter being previously unreported within the *Dolichos* genus. Significantly, compounds **1**, **4**–**7**, and **10**–**12** exhibited marked anti-inflammatory effects through dual mechanisms: (1) potent suppression of NO production and (2) effective downregulation of pro-inflammatory cytokines TNF-α and IL-1β. This investigation not only significantly expands the documented phytochemical diversity of *D. lablab* seeds but also provides crucial molecular-level insights into the mechanistic basis of their traditional anti-inflammatory applications.

## Figures and Tables

**Table 1:** ^1^H and ^13^C NMR data for compound **1** and **2** in CD_3_OD.

No.	1		2	
	δ_C_	δ_H_ (*J* in Hz)	δ_C_	δ_H_ (*J* in Hz)
1	169.7	―	171.4	―
2	119.4	5.76 (s)	122.0	5.87 (s)
3	151.4	―	150.8	―
4	131.9	7.98 (d, 16.0)	137.9	6.64 (d, 15.6)
5	135.0	6.51 (d, 16.0)	133.5	6.51 (d, 15.6)
6	21.3	2.08 (s)	14.2	2.30 (s)
1′	87.6	―	87.6	―
2′	42.8	1.81 (dd, 10.5, 13.0)	42.7	1.82 (dd, 10.2, 13.8)
		2.19 (dd, 6.5, 13.5)		2.20 (dd, 6.6, 12.6)
3′	74.0	4.27 (tdd, 6.5, 10.5, 13.0)	73.9	4.27 (tdd, 6.6, 10.2, 13.8)
4′	42.8	1.79 (t like, ca. 13)	42.8	1.79 (t like, ca. 14)
		1.96 (dd, 6.5, 13.5)		1.96 (dd, 6.6, 12.6)
5′	49.9	―	49.9	―
7′	77.2	3.79 (s)	77.3	3.80 (s)
8′	83.2	―	83.3	―
9′	16.4	0.98 (s)	16.4	0.92 (s)
10′	19.7	1.17 (s)	19.7	1.14 (s)
1″	103.0	4.39 (d, 8.0)	103.1	4.39 (d, 7.8)
2″	75.1	3.16 (dd, 8.0, 8.5)	75.1	3.16 (dd, 7.8, 9.6)
3″	78.1	3.37 (dd, 8.5, 9.5)	78.2	3.36 (dd, 9.6, 9.6)
4″	71.6	3.36 (dd, 9.0, 9.5)	71.6	3.35 (m, overlapped)
5″	76.4	3.51 (m)	76.4	3.51 (m)
6″	67.8	3.74 (br. d, ca. 13)	67.8	3.74 (dd, 1.8, 10.8)
		3.93 (dd, 5.0, 12.5)		3.93 (dd, 5.4, 10.8)
1‴	100.0	4.85 (d, 3.5)	100.1	4.85 (d, 3.6)
2‴	73.8	3.38 (dd, 3.5, 9.5)	73.9	3.38 (dd, 3.6, 9.6)
3‴	75.4	3.65 (dd, 9.5, 9.5)	75.4	3.65 (dd, 9.6, 9.6)
4‴	71.6	3.35 (dd, 9.5, 9.5)	71.6	3.34 (m, overlapped)
5‴	73.6	3.67 (m)	73.7	3.67 (m)
6‴	62.3	3.70 (dd, 5.0, 12.0)	62.6	3.69 (dd, 4.8, 12.2)
		3.80 (br. d, ca. 12)		3.80 (br. d, ca. 12)

**Table 2:** ^1^H NMR data of compounds **3**–**5** in CD_3_OD.

No.	3	4	5
1	3.52 (dd, 9.5, 9.5)	3.48 (dd, 7.8, 11.4)	3.51 (dd, 7.8, 10.8)
	4.19 (br. d, ca. 10)	3.81 (dd, 3.0, 11.4)	3.73 (dd, 3.0, 10.8)
2	3.65 (br. d, ca. 10)	3.68 (dd, 3.0, 7.8)	3.69 (dd, 3.0, 7.8)
4	1.43 (ddd, 5.5, 12.5, 12.5)	1.48 (ddd, 4.8, 12.6, 16.8)	1.38 (ddd, 4.8, 12.0, 16.8)
	1.59 (ddd, 5.5, 12.5, 12.5)	1.70 (ddd, 4.8, 12.6, 16.8)	1.73 (ddd, 4.8, 12.0, 16.8)
5	2.08 (m)	2.08 (m)	2.06 (m)
		2.18 (m)	2.15 (m)
6	5.12 (m)	5.11 (m)	5.01 (m)
8	1.67 (s)	1.67 (s)	1.67 (s)
9	1.62 (s)	1.62 (s)	1.62 (s)
10	1.14 (s)	1.24 (s)	1.27 (s)
1′	4.33 (d, 8.0)	4.51 (d, 7.8)	4.56 (d, 7.8)
2′	3.25 (dd, 8.0, 8.5)	3.18 (dd, 7.8, 9.6)	3.20 (dd, 7.8, 8.4)
3′	3.40 (dd, 8.5, 8.5)	3.37 (dd, 9.0, 9.6)	3.38 (dd,8.4, 9.6)
4′	3.31 (m, overlapped)	3.25 (m, overlapped)	3.35 (dd, 9.0, 9.6)
5′	3.31 (m, overlapped)	3.25 (m, overlapped)	3.49 (m)
6′	3.69 (dd, 4.5, 11.5)	3.62 (dd, 5.4, 12.0)	3.65 (br. d, ca. 10)
	3.87 (br. d, ca. 12)	3.83 (dd, 1.8, 12.0)	3.92 (dd, 4.8, 10.2)
1″			4.83 (d, 3.6)
2″			3.35 (dd, 3.6, 9.6)
3″			3.67 (m, overlapped)
4″			3.32 (dd, 9.6, 9.6)
5″			3.66 (m)
6″			3.68 (m, overlapped)
			3.78 (dd, 3.6, 12.0)

**Table 3:** ^13^C NMR data of compounds **3**–**10** and aglycones **3a** and **6a**.

No.	3 ^a^	3a ^b^	4 ^a^	5 ^a^	6 ^a^	6a ^b^	7 ^a^	8 ^a^	9 ^a^	10 ^a^
1	72.4	63.2	64.2	63.9	63.6	63.4	72.6	116.1	112.1	115.8
2	77.0	76.3	78.1	77.9	91.8	75.5	77.1	144.3	146.2	144.4
3	74.2	74.7	81.9	82.2	74.8	74.6	74.3	81.7	73.8	81.3
4	39.9	37.8	36.9	36.6	39.6	39.1	39.8	42.4	42.9	42.2
5	22.7	22.2	22.7	22.8	22.8	22.3	22.4	23.5	23.5	23.4
6	125.7	124.0	126.0	126.0	125.7	124.1	127.1	126.9	130.4	130.2
7	132.0	132.4	132.1	132.2	132.3	132.2	135.9	135.9	132.7	132.8
8	25.9	25.7	25.9	25.9	25.9	25.7	69.0	69.0	76.2	75.9
9	17.7	17.7	17.8	17.9	17.8	17.7	13.7	13.8	14.1	14.2
10	22.3	23.5	19.8	20.5	22.6	22.2	22.3	23.3	27.6	23.4
1′	104.8		98.1	98.2	104.5		105.0	99.8	102.9	99.5
2′	75.1		75.4	75.5	84.6		75.3	75.3	75.1	75.2
3′	77.7		78.4	78.4	78.0		78.0	78.4	78.2	78.3
4′	71.4		71.9	71.6	71.2		71.6	71.7	71.6	71.7
5′	77.7		77.9	76.2	77.9		78.0	76.0	76.2	77.6
6′	62.6		63.0	67.7	62.5		62.7	67.4	67.1	62.9
1″				100.0	106.4			100.0	99.8	102.6
2″				73.8	75.8			73.9	73.8	75.0
3″				75.2	77.3			75.4	75.3	78.2
4″				71.7	72.9			71.5	71.4	71.7
5″				73.6	77.3			73.5	73.5	77.9
6″				62.6	nd			62.6	62.5	62.8

Determined in ^a^ CD_3_OD and ^b^ CDCl_3_. nd: The signal was not detected.

**Table 4:** ^1^H NMR data for compounds **6** and **7** in CD_3_OD.

No.	6	7	No.	6
1	3.55 (m, overlapped)	3.50 (dd, 9.6, 9.6)	1″	4.70 (d, 8.0)
	3.63 (m, overlapped)	4.20 (dd, 1.8, 9.6)	2″	3.31 (dd, 8.0, 9.0)
2	3.51 (t like, ca. 8)	3.66 (dd, 1.8, 9.6)	3″	3.41 (dd, 9.0, 9.0)
4	1.48 (m)	1.48 (ddd, 5.4, 12.0, 17.4)	4″	3.56 (dd, 8.5, 9.5)
		1.63 (ddd, 5.4, 12.0, 17.4)	5″	3.81 (d, 8.5)
5	2.10 (m)	2.15 (m)		
6	5.10 (m)	5.41 (m)		
8	1.67 (s)	3.91 (s)		
9	1.62 (s)	1.67 (s)		
10	1.10 (s)	1.14 (s)		
1′	4.48 (d, 7.5)	4.30 (d, 7.8)		
2′	3.53 (dd, 7.5, 9.0)	3.22 (dd, 7.8, 9.0)		
3′	3.59 (dd, 8.5, 9.0)	3.37 (dd, 8.4, 9.0)		
4′	3.35 (m, overlapped)	3.28 (dd, 8.4, 8.4)		
5′	3.35 (m, overlapped)	3.27 (m)		
6′	3.64 (dd, 5.0, 12.0)	3.67 (dd, 4.8, 11.4)		
	3.87 (br. d, ca. 12)	3.86 (br.d, ca. 11)		

**Table 5:** ^1^H NMR data for compounds **8**–**10** in CD_3_OD.

No.	8	9	10
1	5.23 (dd, 1.2, 10.8)	5.03 (dd, 1.2, 10.8)	5.21 (br. d, ca. 12)
	5.26 (dd, 1.2, 18.0)	5.20 (dd, 1.2, 17.4)	5.25 (br. d, ca. 18)
2	5.94 (dd, 10.8, 18.0)	5.90 (dd, 10.8, 17.4)	5.94 (dd, 11.5, 17.5)
4	1.64 (m)	1.54 (m)	1.64 (m)
5	2.10 (m)	2.10 (m)	2.12 (m)
6	5.38 (m)	5.49 (m)	5.48 (m)
8	3.90 (s)	4.02 (d, 11.4)	4.04 (d, 11.5)
		4.19 (d, 11.4)	4.20 (d, 11.5)
9	1.63 (s)	1.68 (s)	1.68 (s)
10	1.40 (s)	1.25 (s)	1.39 (s)
1′	4.39 (d, 8.4)	4.27 (d, 7.8)	4.37 (d, 7.5)
2′	3.18 (dd, 8.4, 9.0)	3.21 (d, 7.8, 9.0)	3.20 (m, o)
3′	3.34 (dd, 9.0, 9.0)	3.35 (dd, 9.0, 9.0)	3.37 (dd, 9.0, 9.0)
4′	3.32 (dd, 9.0, 9.0)	3.33 (dd, 9.0, 9.0)	3.31 (m, overlapped)
5′	3.40 (m)	3.42 (m)	3.21 (m, overlapped)
6′	3.60 (dd, 1.8, 10.8)	3.69 (br. d, ca. 11)	3.66 (m, overlapped)
	3.95 (dd, 4.2, 10.8)	3.97 (dd, 3.6, 10.8)	3.82 (br. d, ca. 12)
1″	4.81 (d, 3.6)	4.83 (d, 3.6)	4.26 (d, 7.5)
2″	3.36 (dd, 3.6, 9.6)	3.37 (dd, 3.6, 9.6)	3.21 (m, overlapped)
3″	3.65 (dd, 9.0, 9.6)	3.66 (dd, 9.0, 9.6)	3.31 (m, overlapped)
4″	3.41 (dd, 9.0, 9.0)	3.41 (dd, 9.0, 9.0)	3.31 (m, overlapped)
5″	3.65 (m, overlapped)	3.67 (m, o)	3.24 (m, overlapped)
6″	3.68 (dd, 5.4, 13.8)	3.68 (m, o)	3.66 (m, overlapped)
	3.79 (dd, 4.8, 13.8)	3.79 (dd, 4.8, 10.8)	3.88 (br. d, ca. 12)

**Table 6:** Inhibitory effects of compounds **1**–**14** on NO production in RAW264.7 cells.

No.	NRC (%)	No.	NRC (%)	No.	NRC (%)
N	3.6 ± 0.6	**4**	87.5 ± 5.2 ***	**10**	89.3 ± 3.6 ***
LPS	100 ± 1.7 ^###^	**5**	88.3 ± 3.4 ***	**11**	84.8 ± 2.4 ***
DEX	76.9 ± 3.8 ***	**6**	88.8 ± 0.3 ***	**12**	84.8 ± 2.32 ***
**1**	88.1 ± 4.0 ***	**7**	90.8 ± 0.9 ***	**13**	103.6 ± 7.2
**2**	93.7 ± 2.6	**8**	95.5 ± 3.9	**14**	94.5 ± 1.7
**3**	104.7 ± 6.1	**9**	94.6 ± 3.4		

N—normal group; LPS—LPS-stimulated group; DEX—dexamethasone, a positive group. Nitrite relative concentration (NRC): percentage of control group (set as 100%). Values represent the mean ± SD of six determinations. *** *p* < 0.001 (differences between compound-treated group and LPS-stimulated group). ^###^ *p* < 0.001 (differences between LPS-stimulated group and normal group). Final concentration was 50 μM for compounds **1**, **3**, **8**, **9**, and **11**–**14**; 30 μM for compounds **2**, **4**–**7**, and **10**; 1.5 μg/mL for DEX; and 0.5 μg/mL for LPS.

## Data Availability

The original contributions presented in this study are included in the article/Supplementary Materials. Further inquiries can be directed to the corresponding author(s).

## Supplementary Materials

The following supporting information can be downloaded at: https://www.mdpi.com/article/10.3390/molecules30081779/s1, Figures S1–S88: the NMR, HRESIMS and FT-IR spectra of compounds **1**–**10**; Figure S89: cell viability assay; NMR data of known compounds **11**–**14**, **3a**, **6a** and **8a**.
