# Supplementary material for: In Vitro Anti-Inflammatory Terpenoid Glycosides from the Seeds of Dolichos lablab
Source: Molecules. 2025 Apr 15;30(8):1779. doi: 10.3390/molecules30081779 (PMC12029661; doi:10.3390/molecules30081779)
Supplement: Supplementary file 1 [file molecules-30-01779-s001.zip › molecules-3575219-supplementary.pdf]

Supporting Information

Article

In Vitro Anti-Inflammatory Terpenoid Glycosides from the Seeds of *Dolichos lablab*

Wei Zhang[1,#], Jingya Ruan[2,#], Jiaming Cheng[1], Yingying Wang[2], Yinuo Zheng[1], Minghao Lin [1], Yi Zhang[1,2*] and Tao Wang[1,2*]

[1] Tianjin Key Laboratory of TCM Chemistry and Analysis, Tianjin University of Traditional Chinese Medicine, 10 Poyanghu Road, West Area, Tuanbo New Town, Jinghai District, 301617, Tianjin, China; zhangwei940905@163.com (W.Z.); c1584172707@163.com (J.-M.C.); zhengyinuo1002@163.com (Y.-N.Z.); Linminghao02@163.com (M.-H.L.);

[2] Institute of TCM, Tianjin University of Traditional Chinese Medicine, 10 Poyanghu Road, West Area, Tuanbo New Town, Jinghai District, 301617, Tianjin, China; ruanjingya@ tjutcm.edu.cn (J.-Y.R.); 15007283268@163.com (Y.-Y.W.)

[#] These authors contributed equally;

* Correspondence: zhwwxzh@tjutcm.edu.cn (Y.Z.); wangtao@tjutcm.edu.cn (T.W.); Tel./Fax: +86-22-5959-6168 (T.W.);

Figure S1 ^{1}H NMR (500 MHz, CD_3OD) spectrum of **1** 5
Figure S2 ^{13}C NMR (125 MHz, CD_3OD) spectrum of **1** 5
Figure S3 ^{1}H ^{1}H COSY (CD_3OD) spectrum of **1** 6
Figure S4 HSQC (CD_3OD) spectrum of **1** 6
Figure S5 HMBC (CD_3OD) spectrum of **1** 7
Figure S6 NOESY (CD_3OD) spectrum of **1** 7
Figure S7 ESI-Q-Orbitrap-MS spectrum of **1** 8
Figure S8 IR spectrum of **1** 8
Figure S9 ^{1}H NMR (600 MHz, CD_3OD) spectrum of **2** 9
Figure S10 ^{13}C NMR (150 MHz, CD_3OD) spectrum of **2** 9
Figure S11 ^{1}H ^{1}H COSY (CD_3OD) spectrum of **2** 10
Figure S12 HSQC (CD_3OD) spectrum of **2** 10
Figure S13 HMBC (CD_3OD) spectrum of **2** 11
Figure S14 NOESY (CD_3OD) spectrum of **2** 11
Figure S15 ESI-Q-Orbitrap-MS spectrum of **2** 12
Figure S16 IR spectrum of **2** 12
Figure S17 ^{1}H NMR (500 MHz, CD_3OD) spectrum of **3** 13
Figure S18 ^{13}C NMR (125 MHz, CD_3OD) spectrum of **3** 13
Figure S19 ^{1}H ^{1}H COSY (CD_3OD) spectrum of **3** 14
Figure S20 HSQC (CD_3OD) spectrum of **3** 14
Figure S21 HMBC (CD_3OD) spectrum of **3** 15
Figure S22 NOESY (CD_3OD) spectrum of **3** 15
Figure S23 ESI-Q-Orbitrap-MS spectrum of **3** 16
Figure S24 IR spectrum of **3** 16
Figure S25 ^{1}H NMR (500 MHz, $CDCl_3$) spectrum of **3a** 17
Figure S26 ^{13}C NMR (125 MHz, $CDCl_3$) spectrum of **3a** 17
Figure S27 HSQC ($CDCl_3$) spectrum of **3a** 18
Figure S28 ESI-Q-Orbitrap-MS spectrum of **3a** 18
Figure S29 ^{1}H NMR (600 MHz, CD_3OD) spectrum of **4** 19
Figure S30 ^{13}C NMR (150 MHz, CD_3OD) spectrum of **4** 19
Figure S31 ^{1}H ^{1}H COSY (CD_3OD) spectrum of **4**....20
Figure S32 HSQC (CD_3OD) spectrum of **4** 20
Figure S33 HMBC (CD_3OD) spectrum of **4** 21
Figure S34 NOESY (CD_3OD) spectrum of **4** 21

Figure S35 ESI-Q-Orbitrap-MS spectrum of **4** 22
Figure S36 IR spectrum of **4** 22
Figure S37 ^{1}H NMR (600 MHz, CD_3OD) spectrum of **5** 23
Figure S38 ^{13}C NMR (150 MHz, CD_3OD) spectrum of **5** 23
Figure S39 ^{1}H ^{1}H COSY (CD_3OD) spectrum of **5** 24
Figure S40 HSQC (CD_3OD) spectrum of **5** 24
Figure S41 HMBC (CD_3OD) spectrum of **5** 25
Figure S42 NOESY (CD_3OD) spectrum of **5** 25
Figure S43 ESI-Q-Orbitrap-MS spectrum of **5** 26
Figure S44 IR spectrum of **5** 26
Figure S45 ^{1}H NMR (500 MHz, CD_3OD) spectrum of **6** 27
Figure S46 ^{13}C NMR (125 MHz, CD_3OD) spectrum of **6** 27
Figure S47 ^{1}H ^{1}H COSY (CD_3OD) spectrum of **6** 28
Figure S48 HSQC (CD_3OD) spectrum of **6** 28
Figure S49 HMBC (CD_3OD) spectrum of **6** 29
Figure S50 ESI-Q-Orbitrap-MS spectrum of **6** 29
Figure S51 IR spectrum of **6** 30
Figure S52 ^{1}H NMR (500 MHz, $CDCl_3$) spectrum of **6a** 31
Figure S53 ^{13}C NMR (125 MHz, $CDCl_3$) spectrum of **6a** 31
Figure S54 HSQC ($CDCl_3$) spectrum of **6a** 32
Figure S55 ESI-Q-Orbitrap-MS spectrum of **6a** 32
Figure S56 ^{1}H NMR (600 MHz, CD_3OD) spectrum of **7** 33
Figure S57 ^{13}C NMR (150 MHz, CD_3OD) spectrum of **7** 33
Figure S58 ^{1}H ^{1}H COSY (CD_3OD) spectrum of **7** 34
Figure S59 HSQC (CD_3OD) spectrum of **7** 34
Figure S60 HMBC (CD_3OD) spectrum of **7** 35
Figure S61 NOESY (CD_3OD) spectrum of **7** 35
Figure S62 ESI-Q-Orbitrap-MS spectrum of **7** 36
Figure S63 IR spectrum of **7** 36
Figure S64 ^{1}H NMR (600 MHz, CD_3OD) spectrum of **8** 37
Figure S65 ^{13}C NMR (150 MHz, CD_3OD) spectrum of **8** 37
Figure S66 ^{1}H ^{1}H COSY (CD_3OD) spectrum of **8** 38
Figure S67 HSQC (CD_3OD) spectrum of **8** 38
Figure S68 HMBC (CD_3OD) spectrum of **8** 39

Figure S69 NOESY (CD_3OD) spectrum of **8** 39
Figure S70 ESI-Q-Orbitrap-MS spectrum of **8** 40
Figure S71 IR spectrum of **8** 40
Figure S72 ^1H NMR (600 MHz, C_5D_5N) spectrum of **8a** 41
Figure S73 ESI-Q-Orbitrap-MS spectrum of **8a** 41
Figure S74 ^1H NMR (600 MHz, CD_3OD) spectrum of **9** 42
Figure S75 ^{13}C NMR (150 MHz, CD_3OD) spectrum of **9** 42
Figure S76 ^1H ^1H COSY (CD_3OD) spectrum of **9** 43
Figure S77 HSQC (CD_3OD) spectrum of **9** 43
Figure S78 HMBC (CD_3OD) spectrum of **9** 44
Figure S79 NOESY (CD_3OD) spectrum of **9** 44
Figure S80 ESI-Q-Orbitrap-MS spectrum of **9** 45
Figure S81 IR spectrum of **9** 45
Figure S82 ^1H NMR (500 MHz, CD_3OD) spectrum of **10** 46
Figure S83 ^{13}C NMR (125 MHz, CD_3OD) spectrum of **10** 46
Figure S84 ^1H ^1H COSY (CD_3OD) spectrum of **10** 47
Figure S85 HSQC (CD_3OD) spectrum of **10** 47
Figure S86 HMBC (CD_3OD) spectrum of **10** 48
Figure S87 ESI-Q-Orbitrap-MS spectrum of **10** 48
Figure S88 IR spectrum of **10** 49
Figure S89 MTT assay of compounds **1–14** 50
The physical data of compounds **1–14**, **3a**, **6a** and **8a** 51

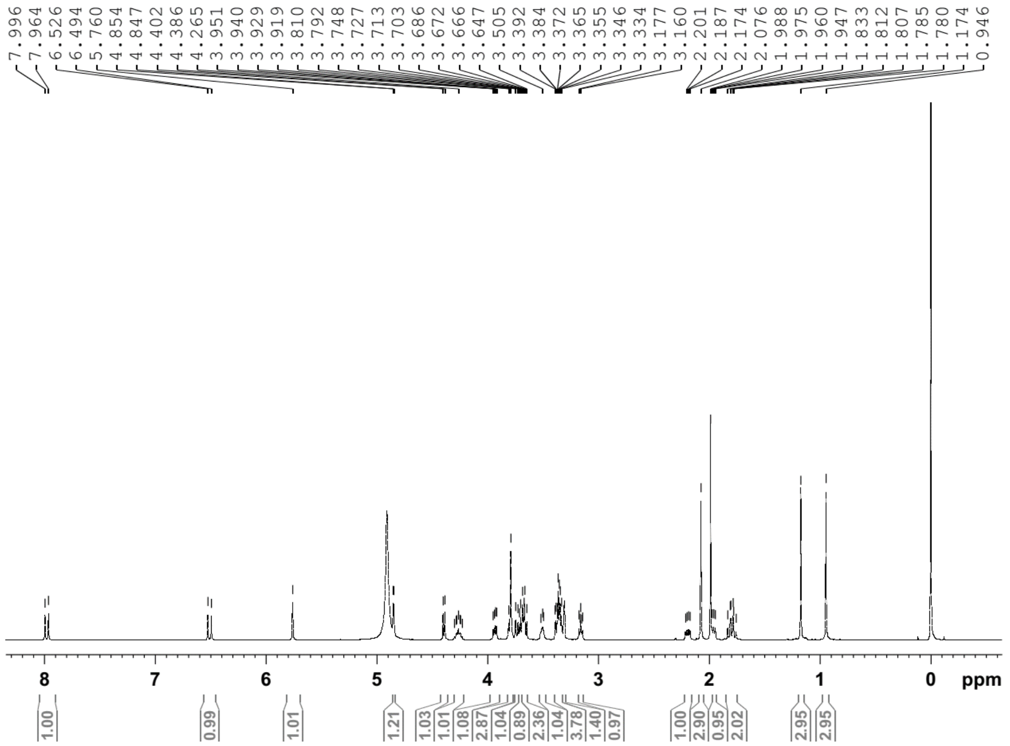

Figure S1 ^{1}H NMR (500 MHz, CD_3OD) spectrum of **1**

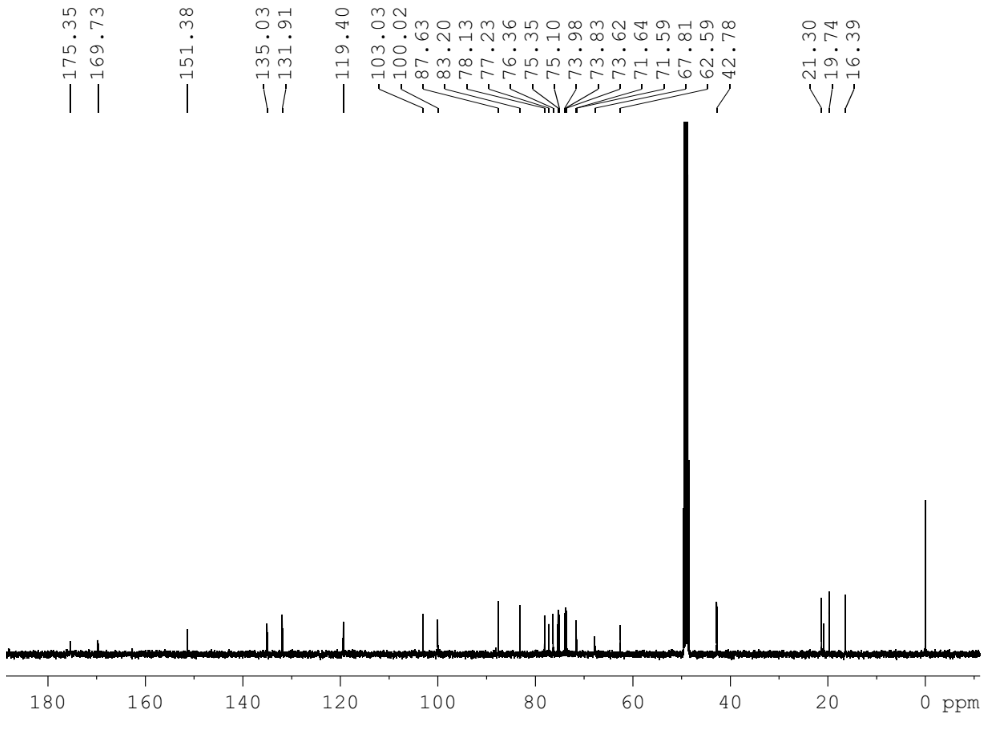

Figure S2 ^{13}C NMR (125 MHz, CD_3OD) spectrum of **1**

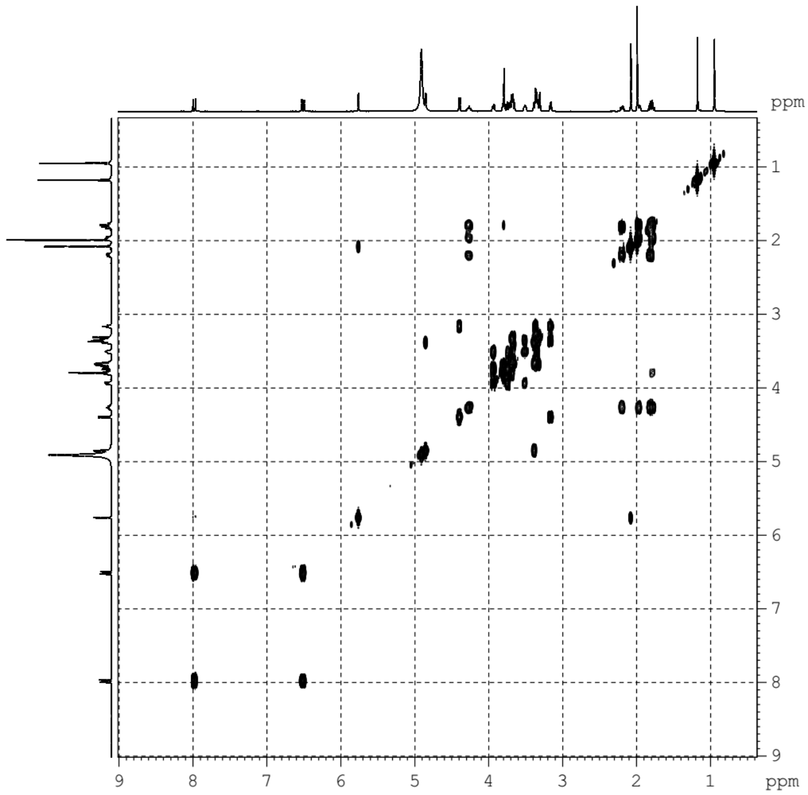

Figure S3 ^{1}H ^{1}H COSY (CD_3OD) spectrum of **1**

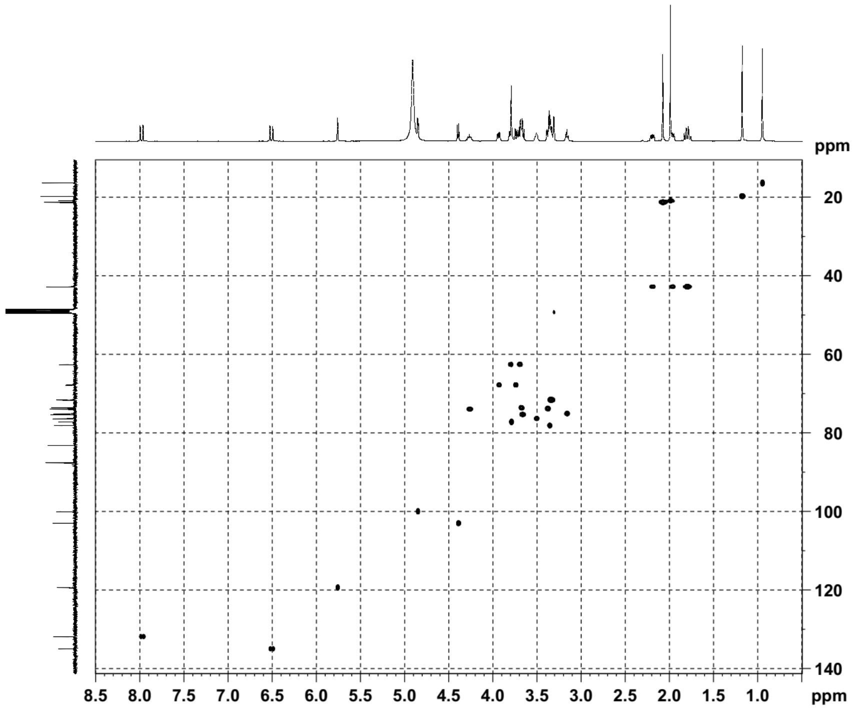

Figure S4 HSQC (CD_3OD) spectrum of **1**

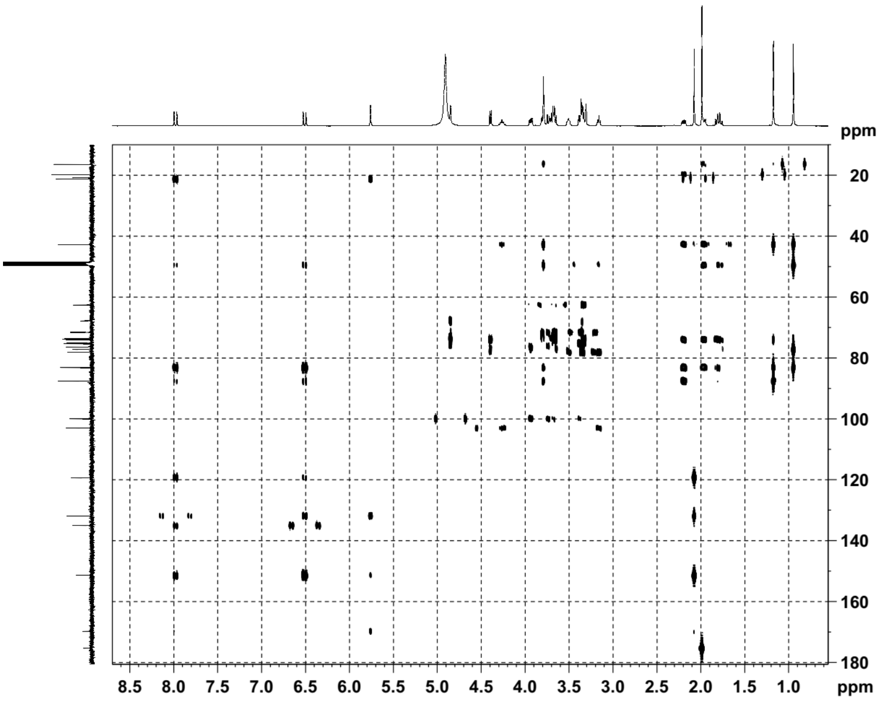

Figure S5 HMBC (CD_3OD) spectrum of **1**

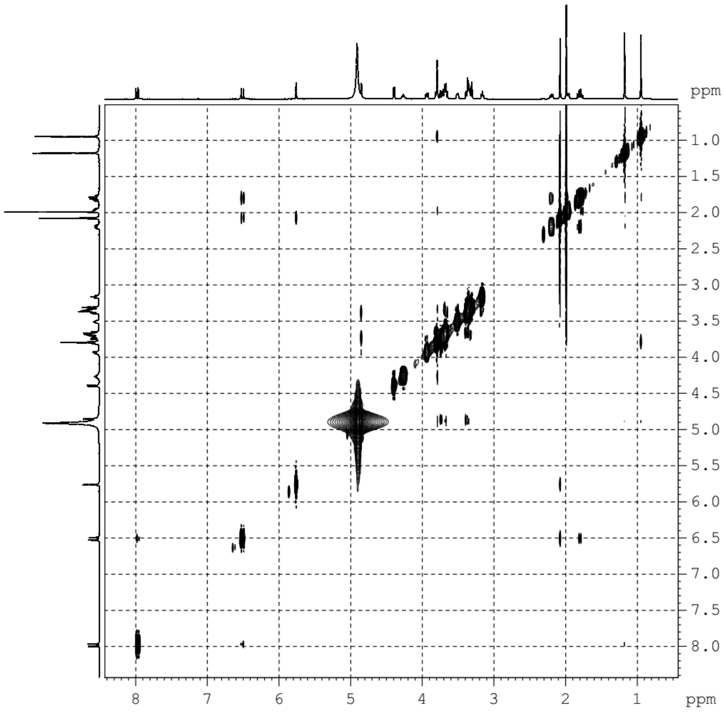

Figure S6 NOESY (CD_3OD) spectrum of **1**

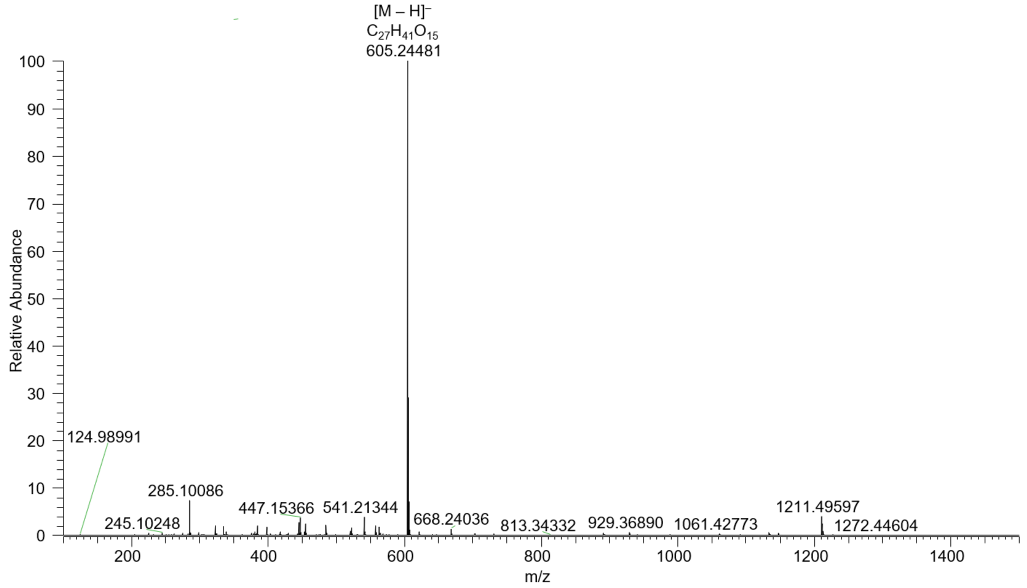

Figure S7 ESI-Q-Orbitrap-MS spectrum of **1**

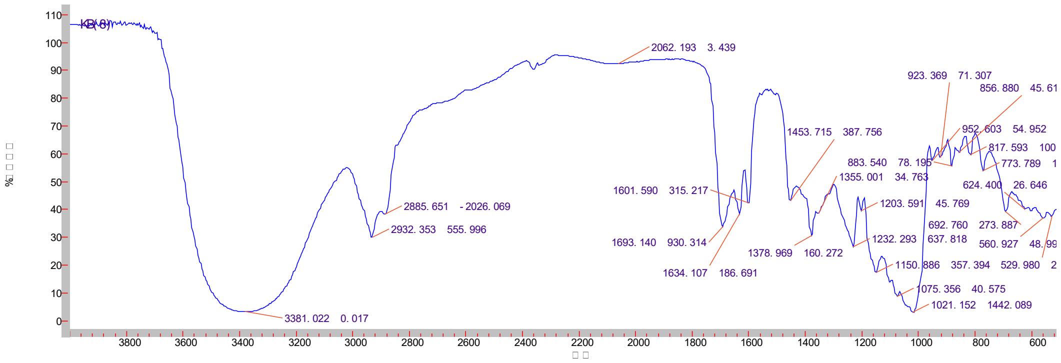

Figure S8 IR spectrum of **1**

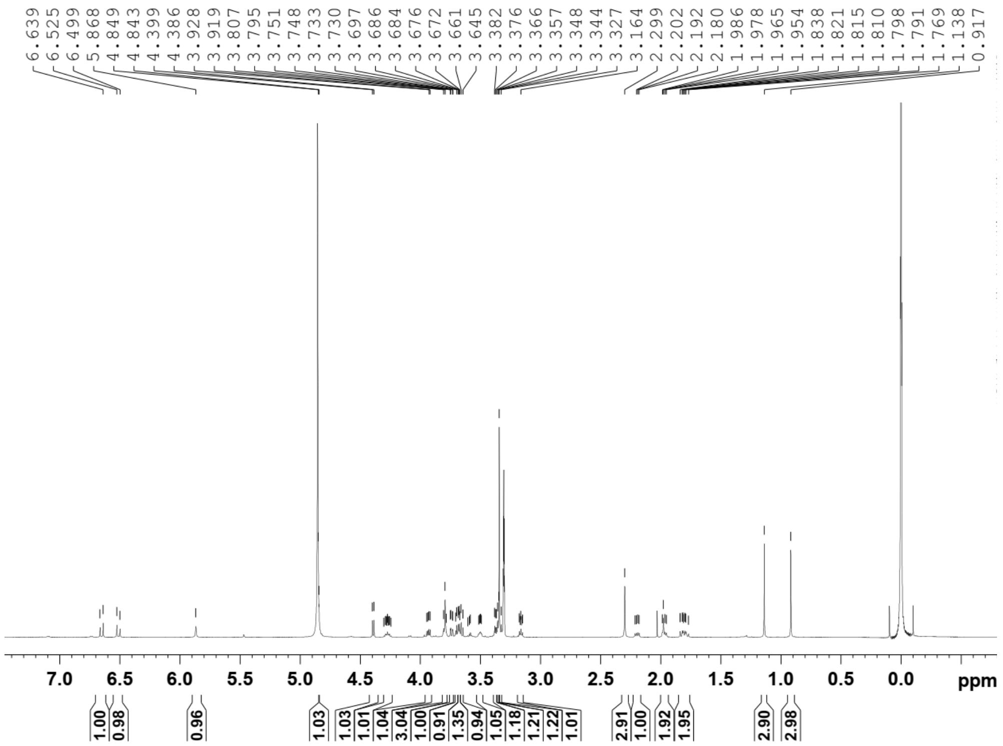

Figure S9 ^{1}H NMR (600 MHz, CD_3OD) spectrum of **2**

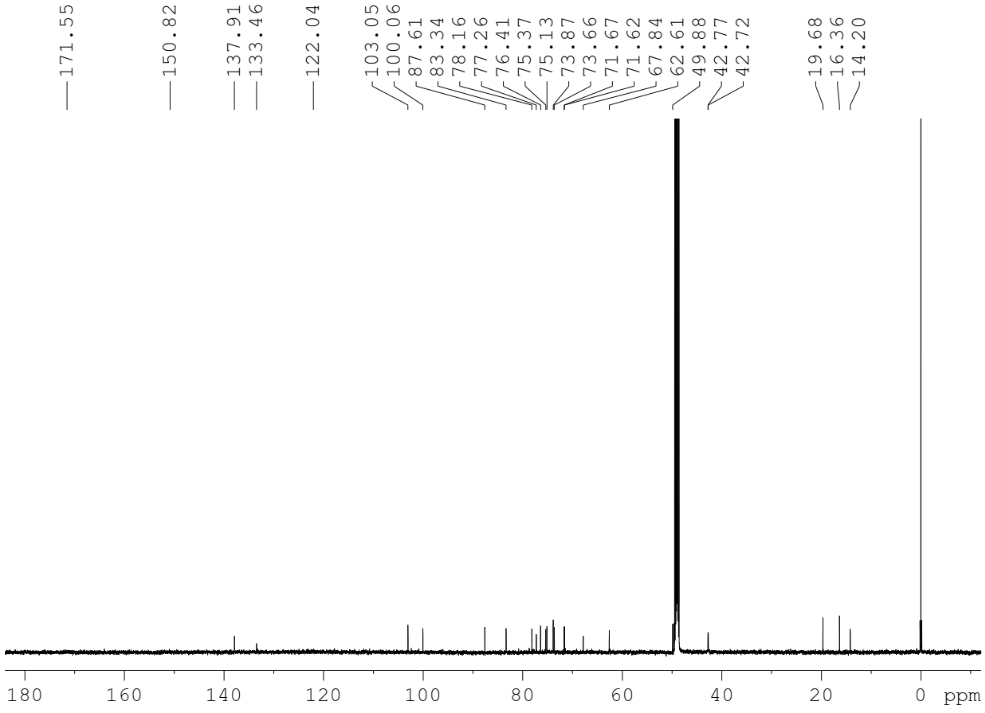

Figure S10 ^{13}C NMR (150 MHz, CD_3OD) spectrum of **2**

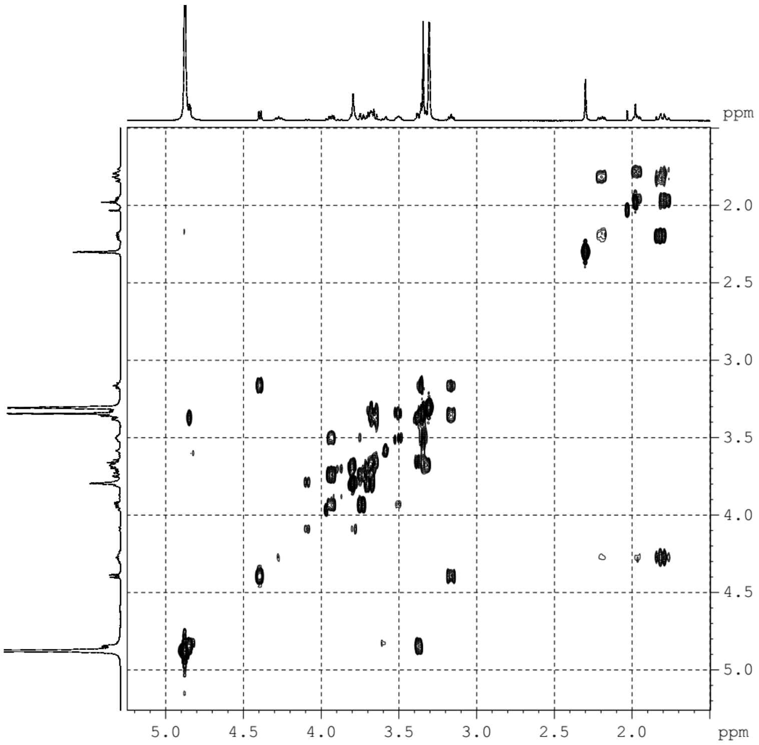

Figure S11 ^{1}H ^{1}H COSY (CD_3OD) spectrum of **2**

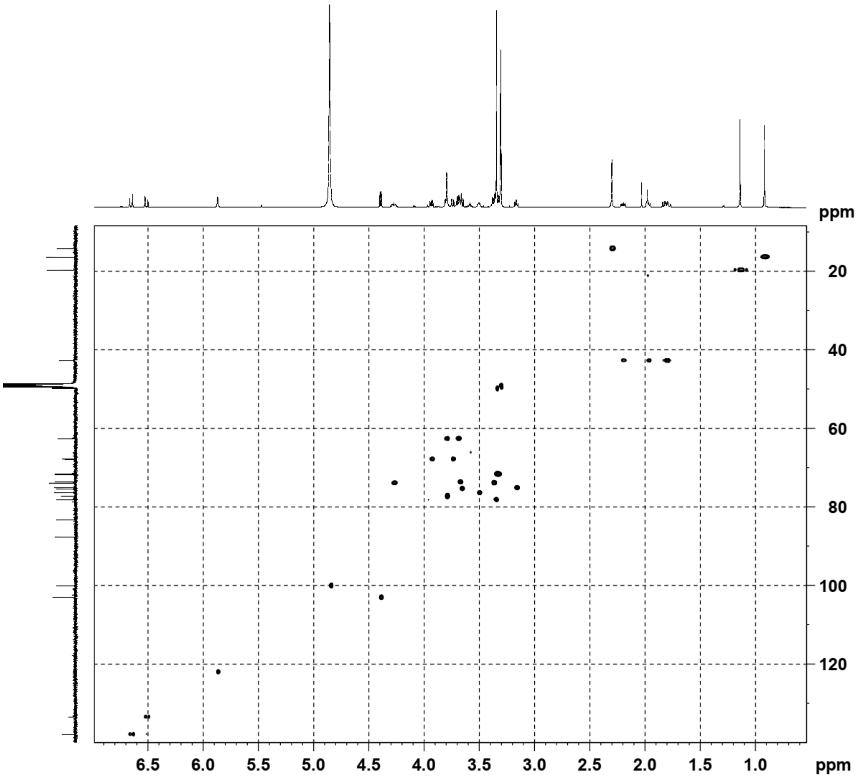

Figure S12 HSQC (CD_3OD) spectrum of **2**

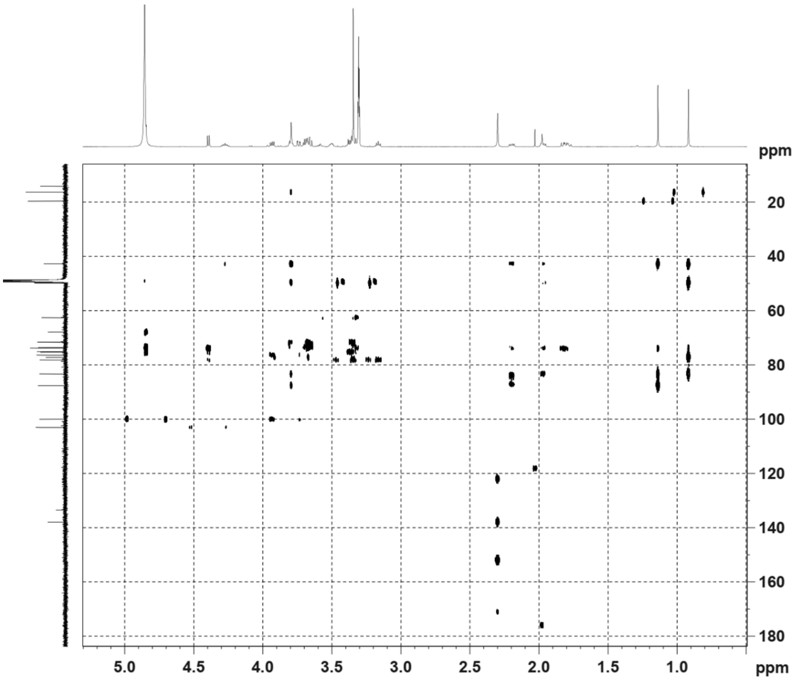

Figure S13 HMBC (CD_3OD) spectrum of **2**

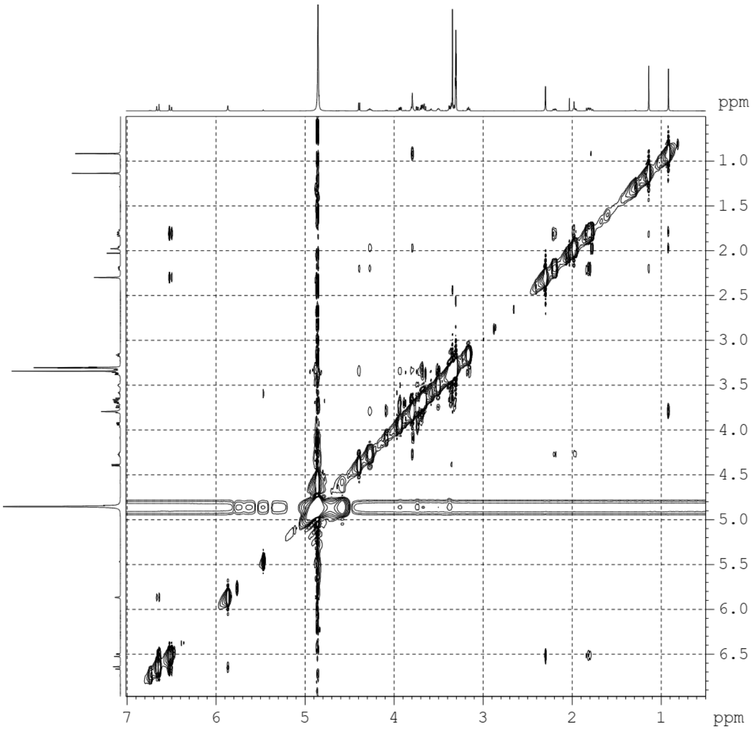

Figure S14 NOESY (CD_3OD) spectrum of **2**

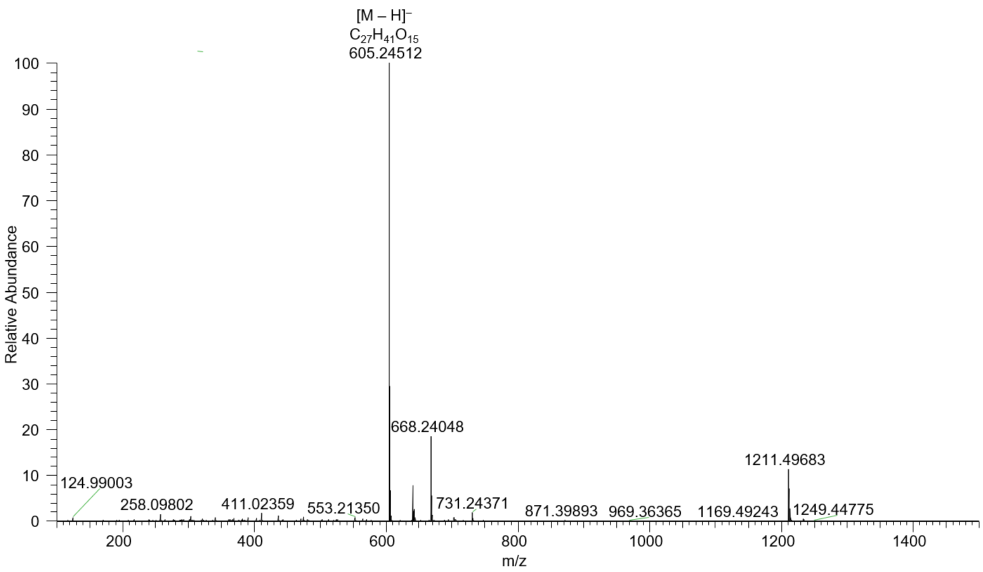

Figure S15 ESI-Q-Orbitrap-MS spectrum of **2**

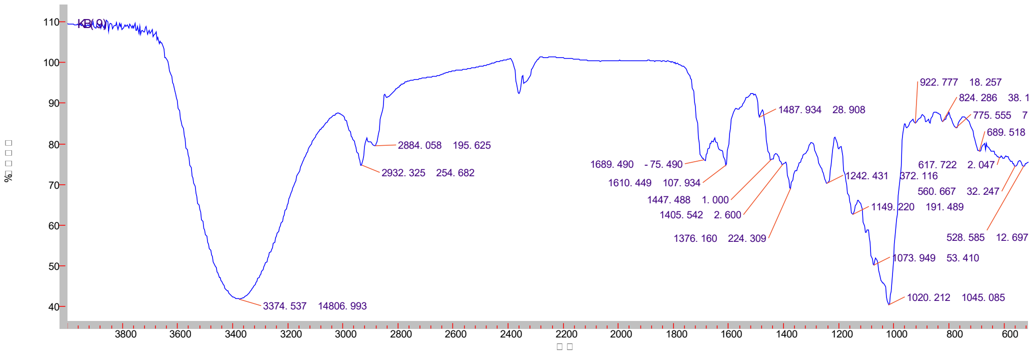

Figure S16 IR spectrum of **2**

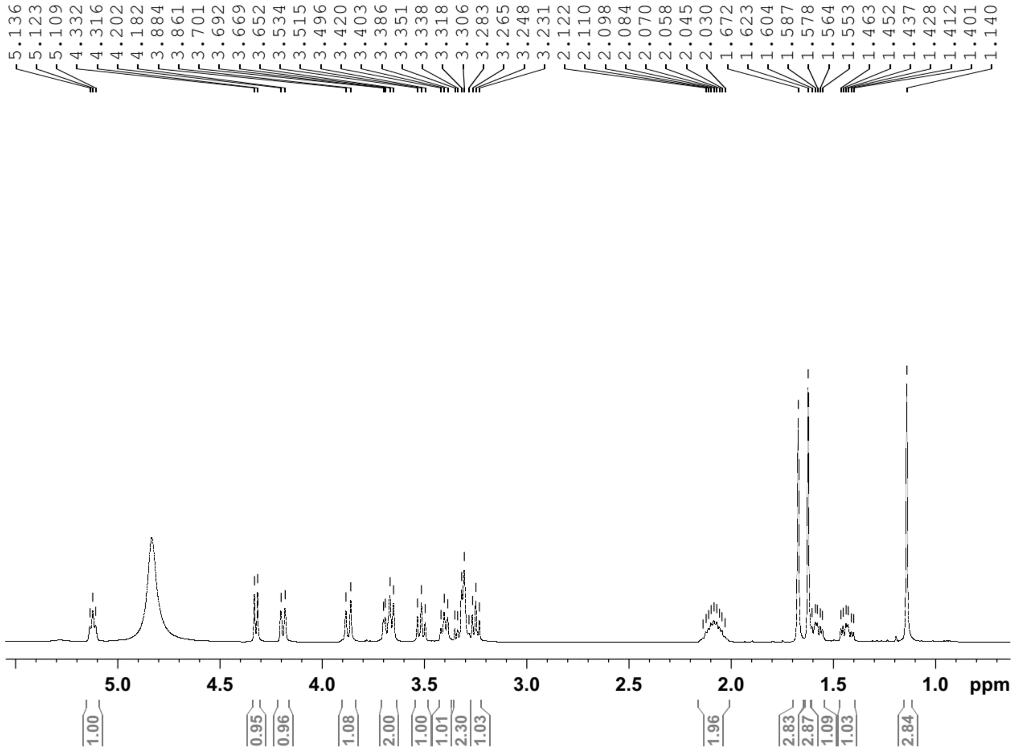

Figure S17 ^{1}H NMR (500 MHz, CD_3OD) spectrum of **3**

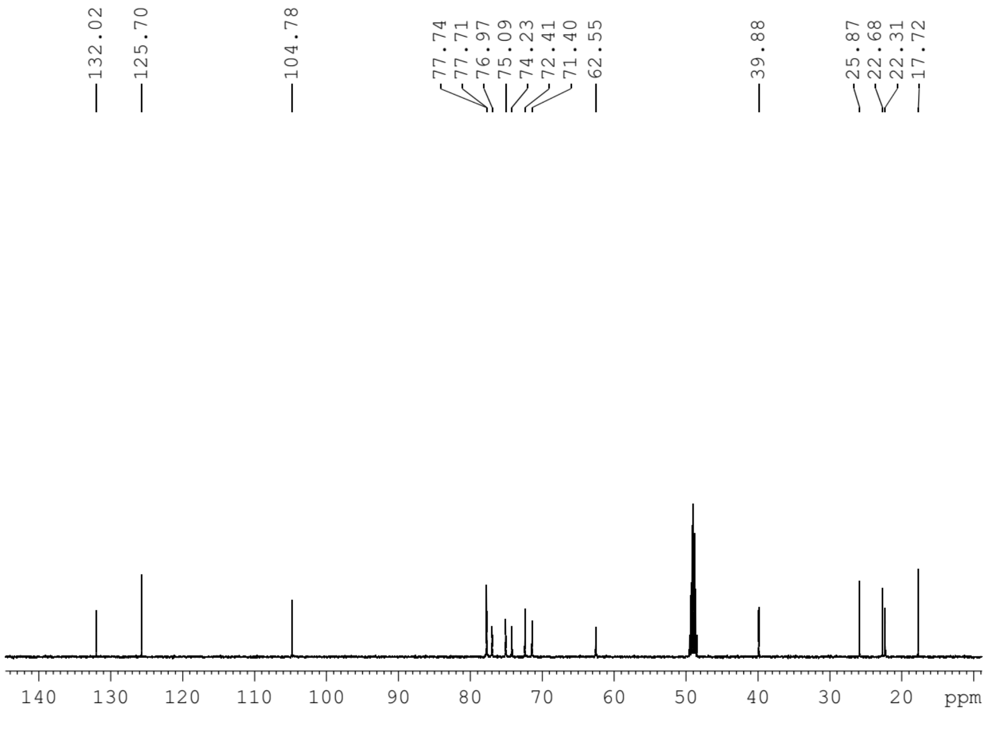

Figure S18 ^{13}C NMR (125 MHz, CD_3OD) spectrum of **3**

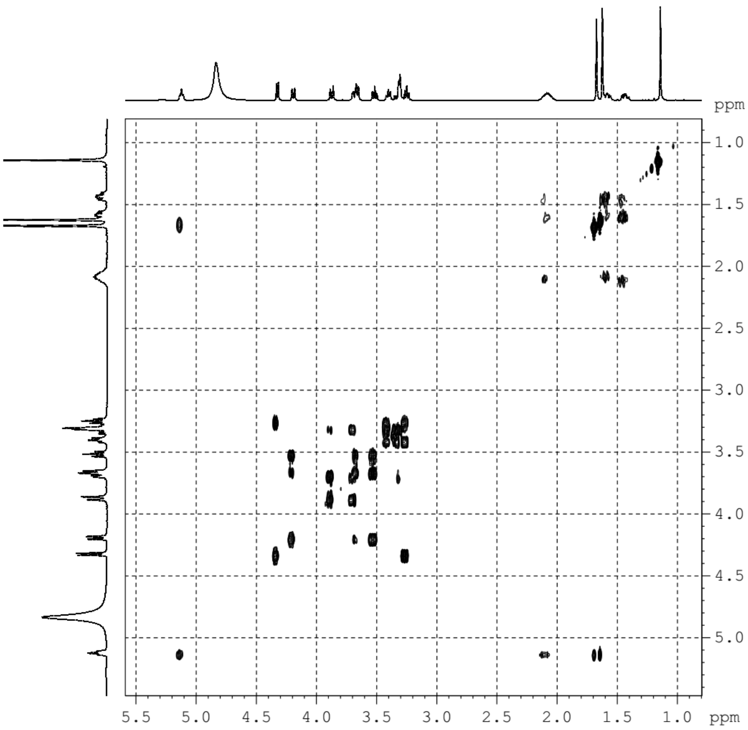

Figure S19 ^{1}H ^{1}H COSY (CD_3OD) spectrum of **3**

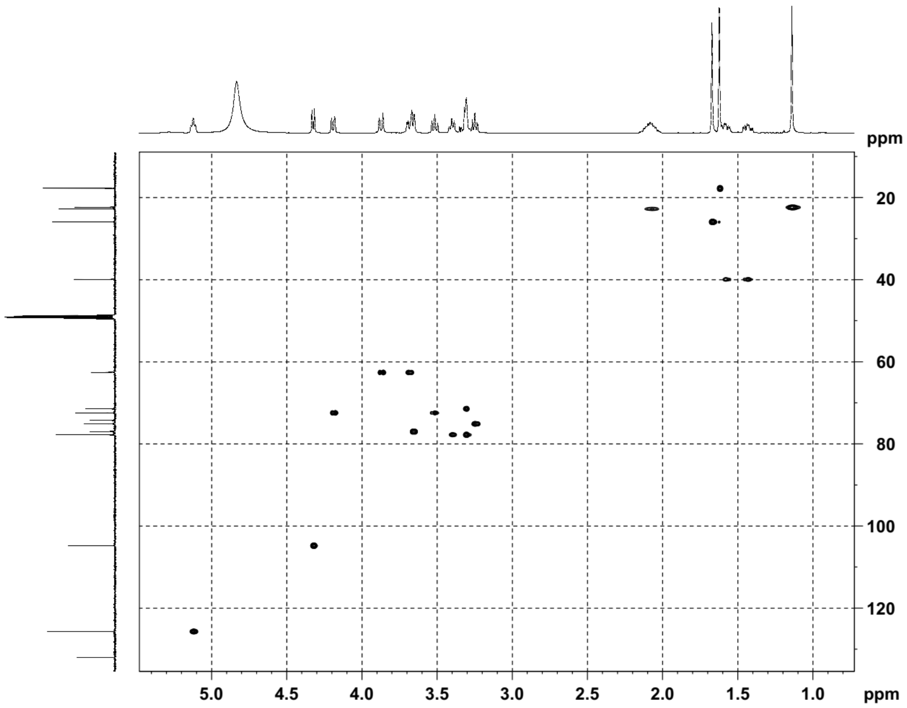

Figure S20 HSQC (CD_3OD) spectrum of **3**

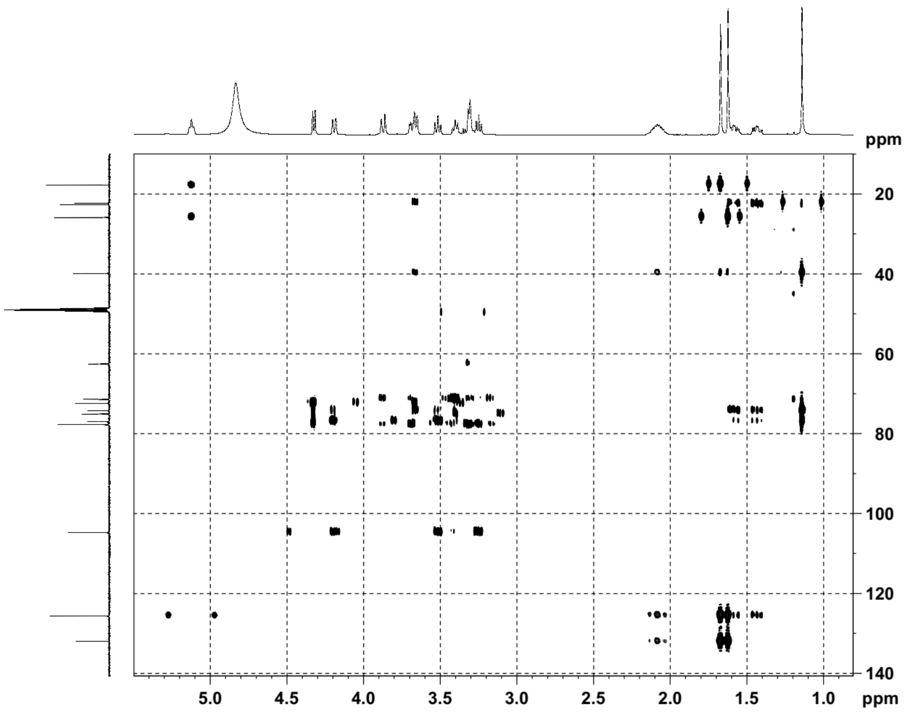

Figure S21 HMBC (CD_3OD) spectrum of **3**

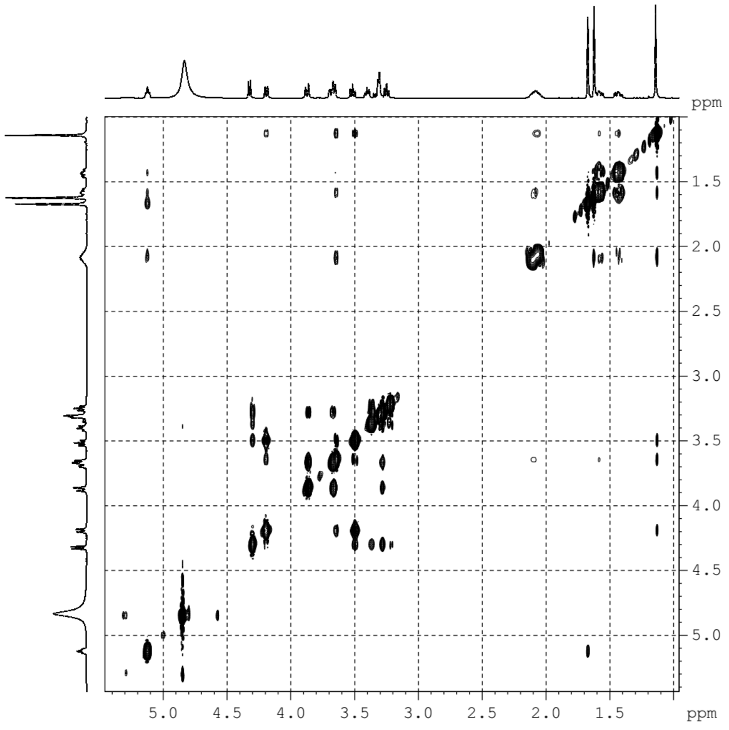

Figure S22 NOESY (CD_3OD) spectrum of **3**

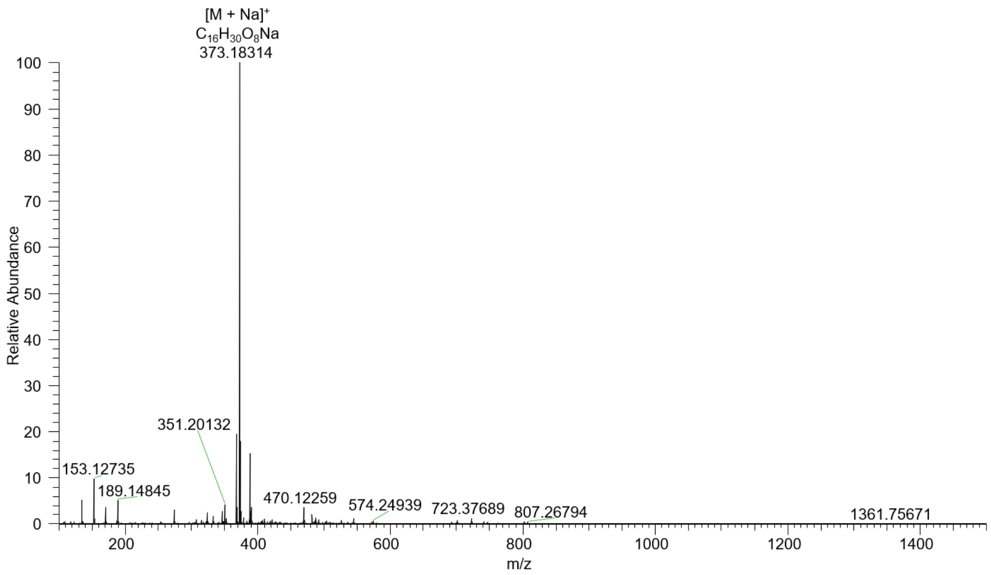

Figure S23 ESI-Q-Orbitrap-MS spectrum of **3**

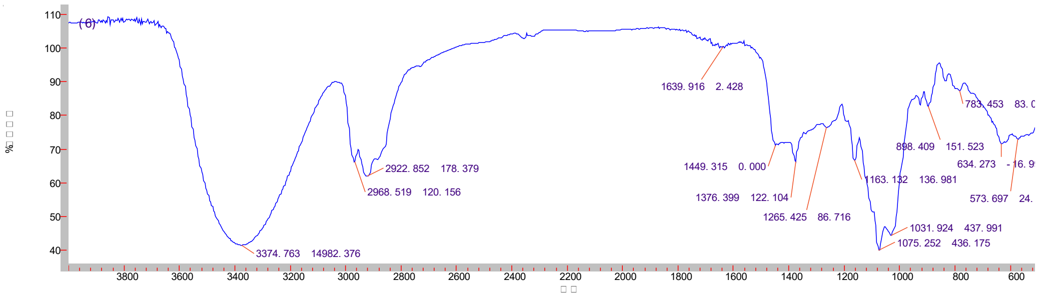

Figure S24 IR spectrum of **3**

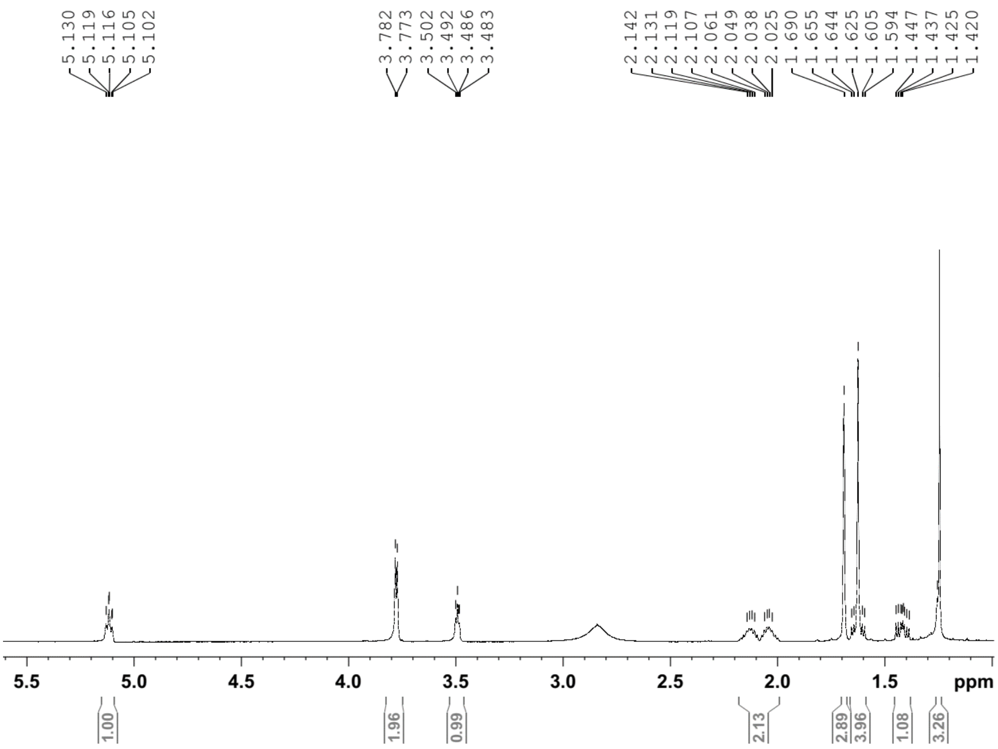

Figure S25 ^{1}H NMR (500 MHz, $CDCl_3$) spectrum of **3a**

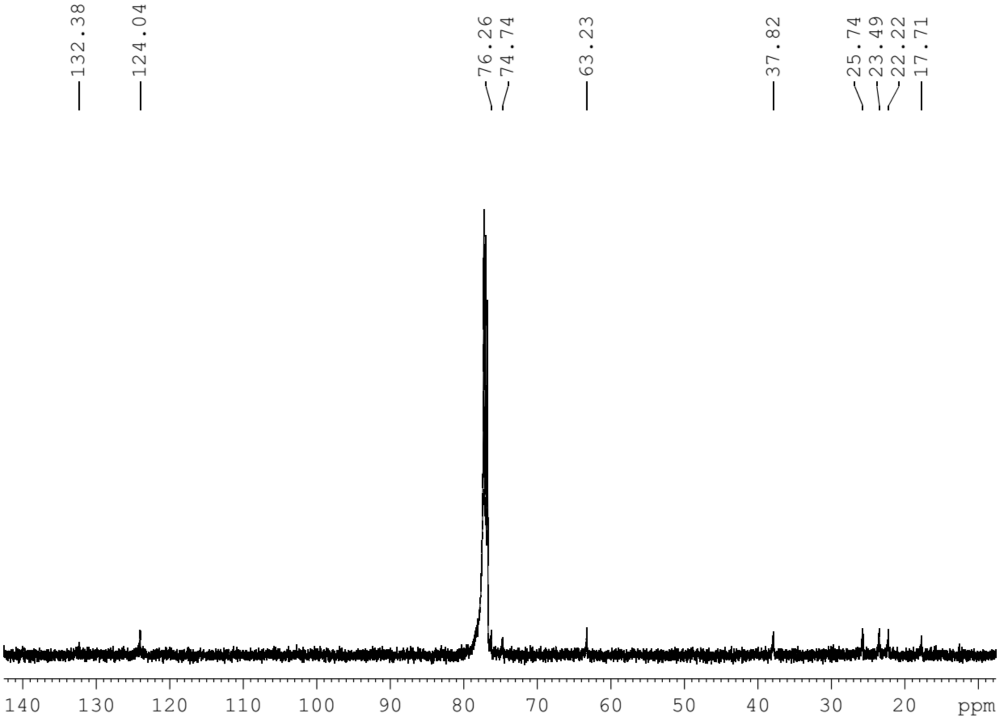

Figure S26 ^{13}C NMR (125 MHz, $CDCl_3$) spectrum of **3a**

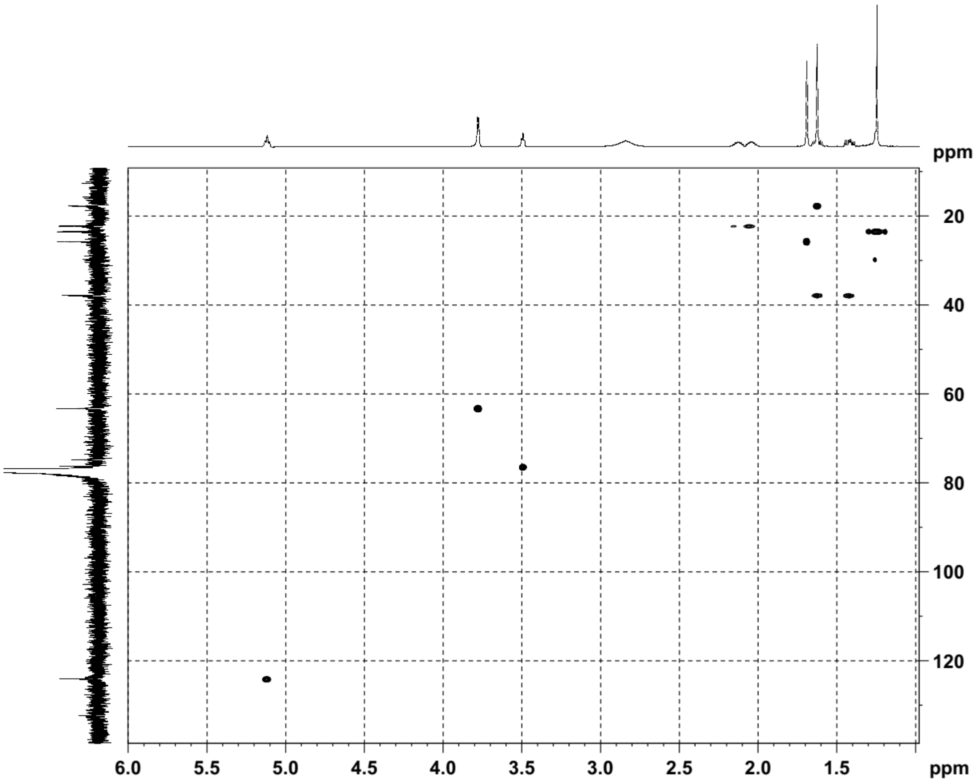

Figure S27 HSQC ($CDCl_3$) spectrum of **3a**

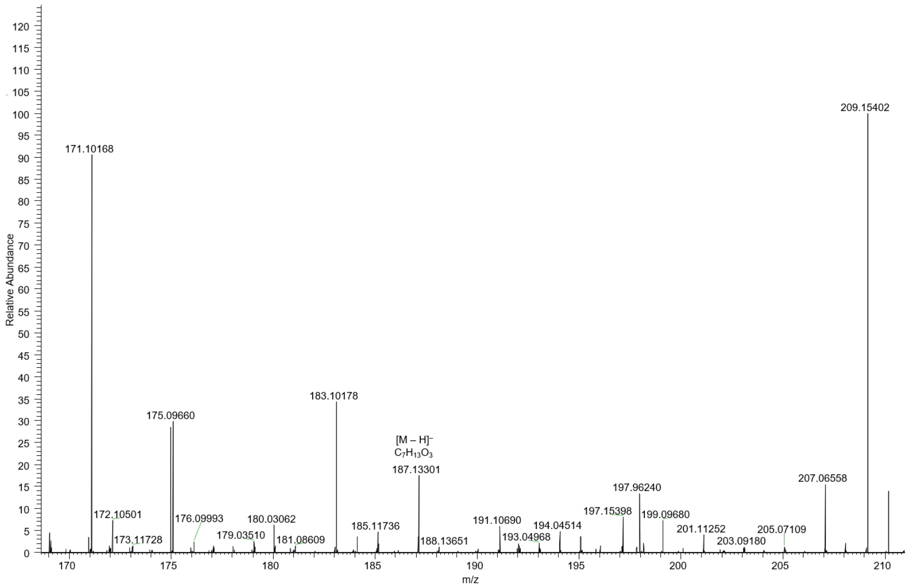

Figure S28 ESI-Q-Orbitrap-MS spectrum of **3a**

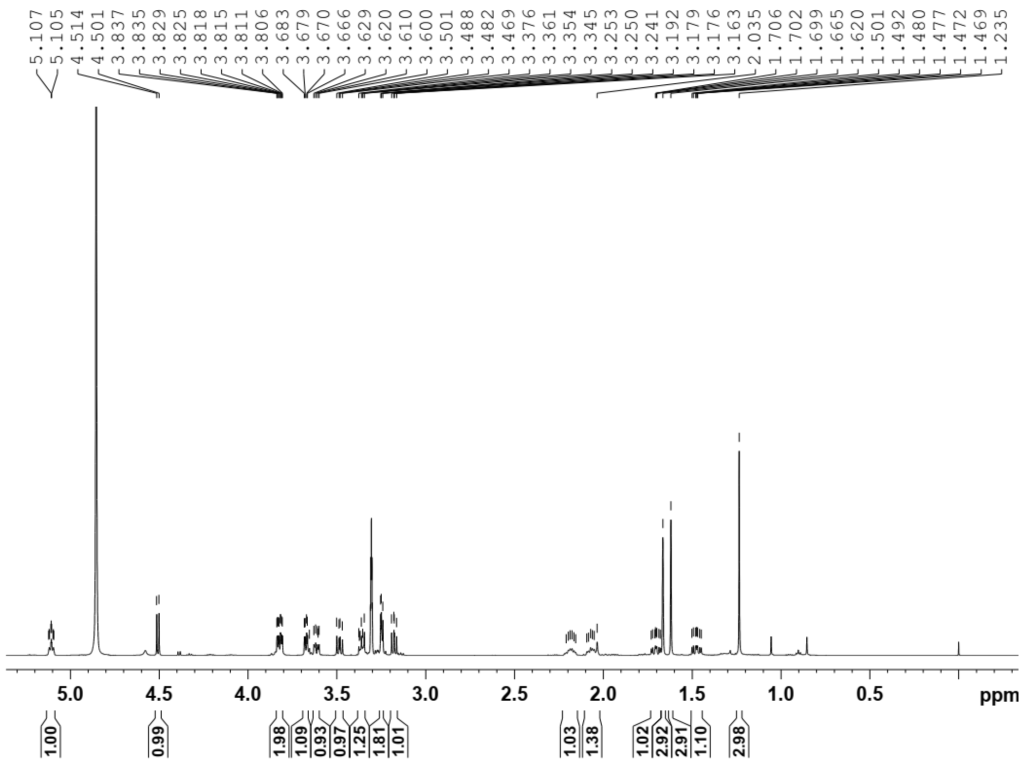

Figure S29 ^{1}H NMR (600 MHz, CD_3OD) spectrum of **4**

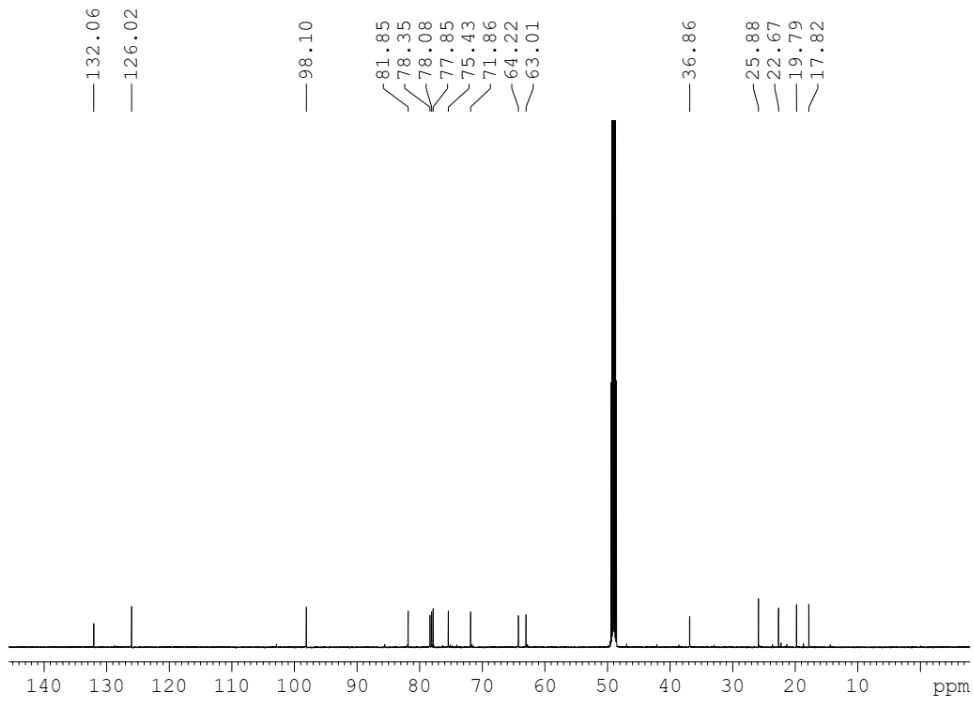

Figure S30 ^{13}C NMR (150 MHz, CD_3OD) spectrum of **4**

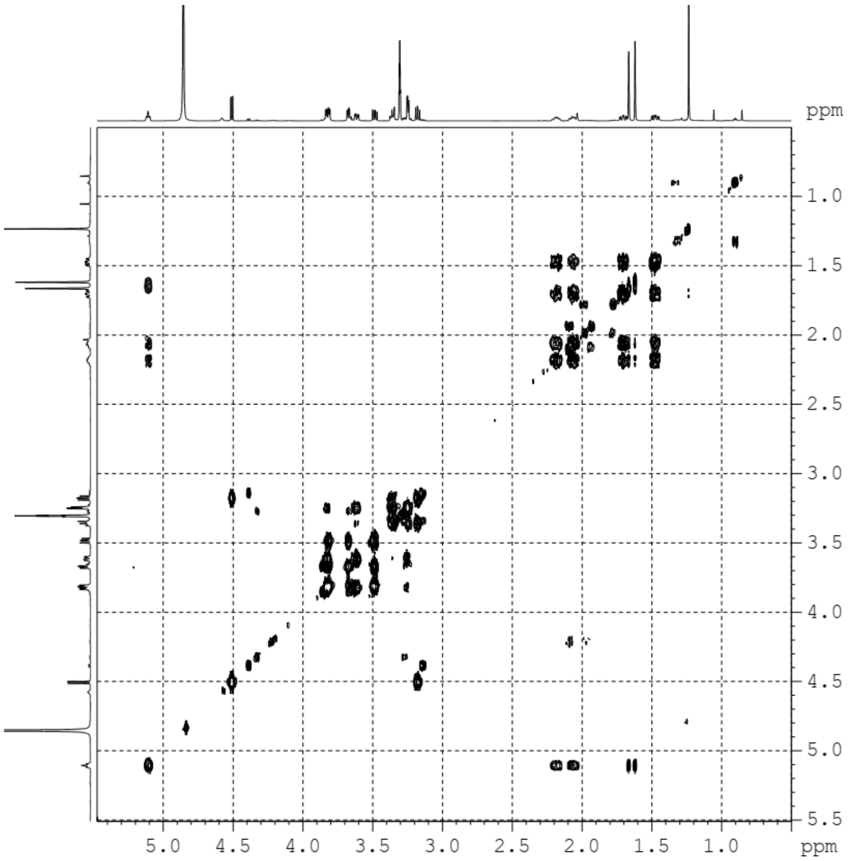

Figure S31 ^{1}H ^{1}H COSY (CD_3OD) spectrum of **4**

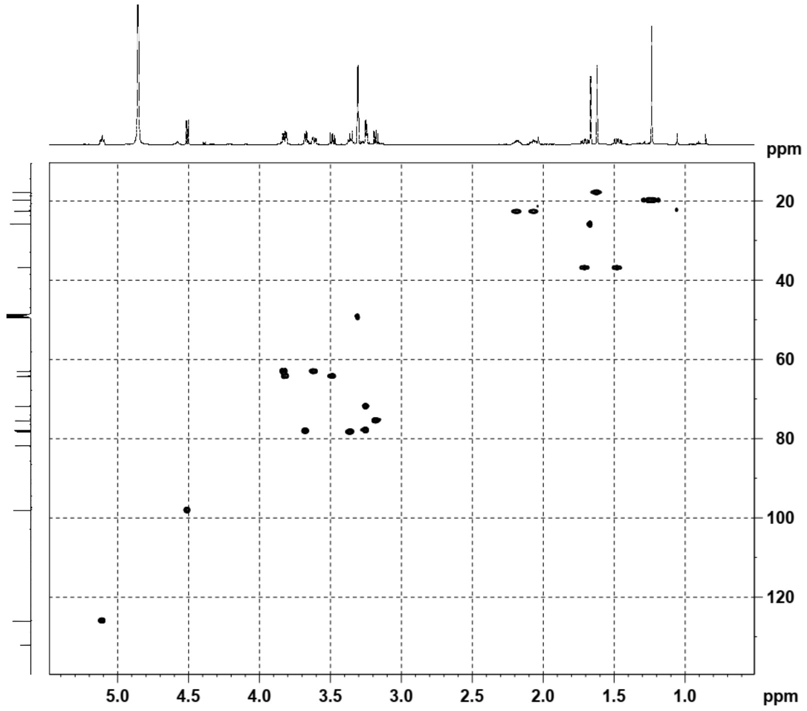

Figure S32 HSQC (CD_3OD) spectrum of **4**

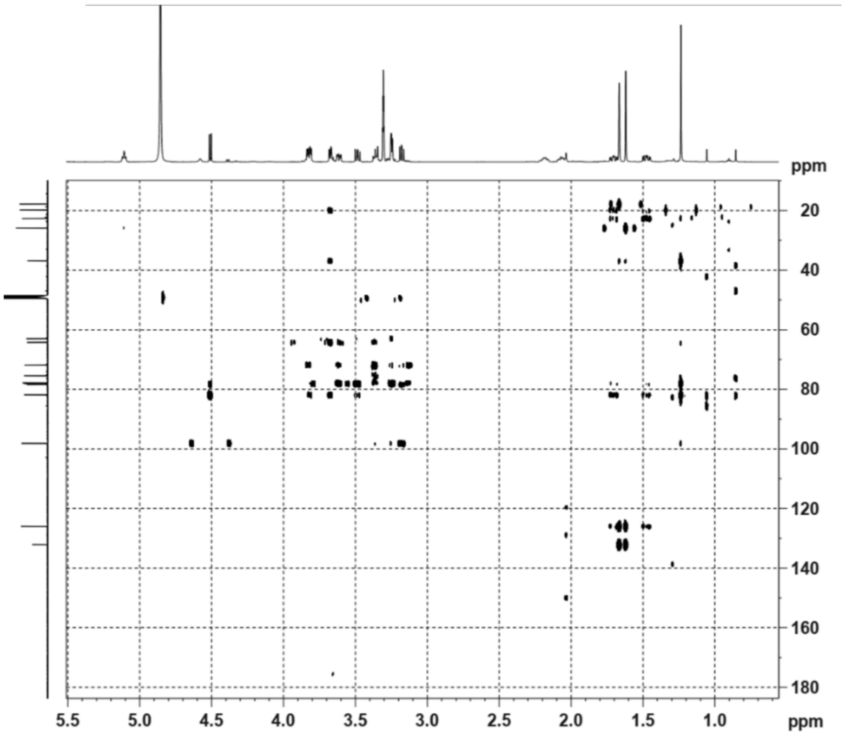

Figure S33 HMBC (CD_3OD) spectrum of **4**

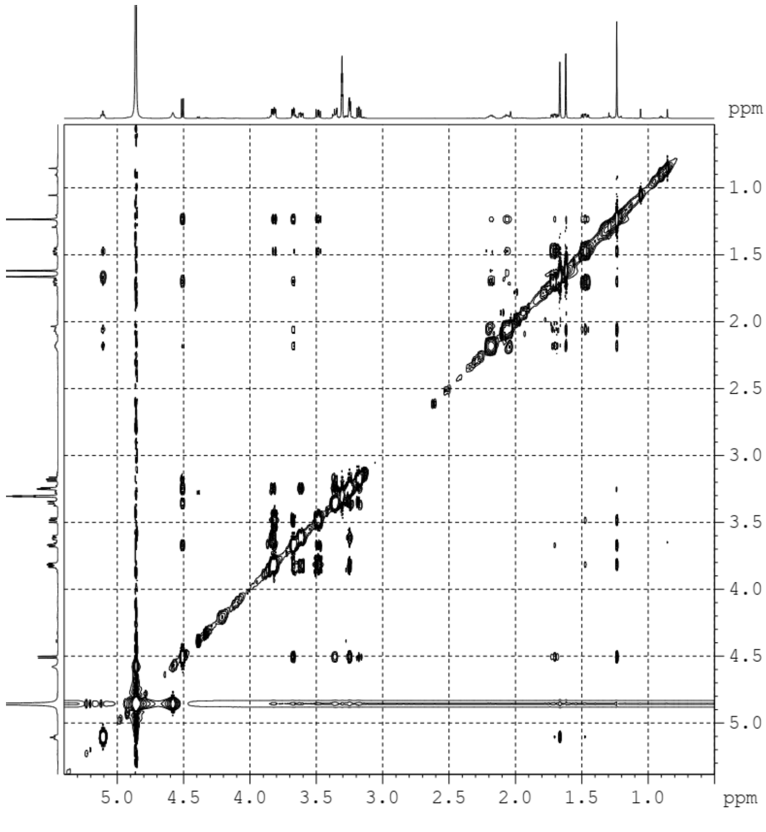

Figure S34 NOESY (CD_3OD) spectrum of **4**

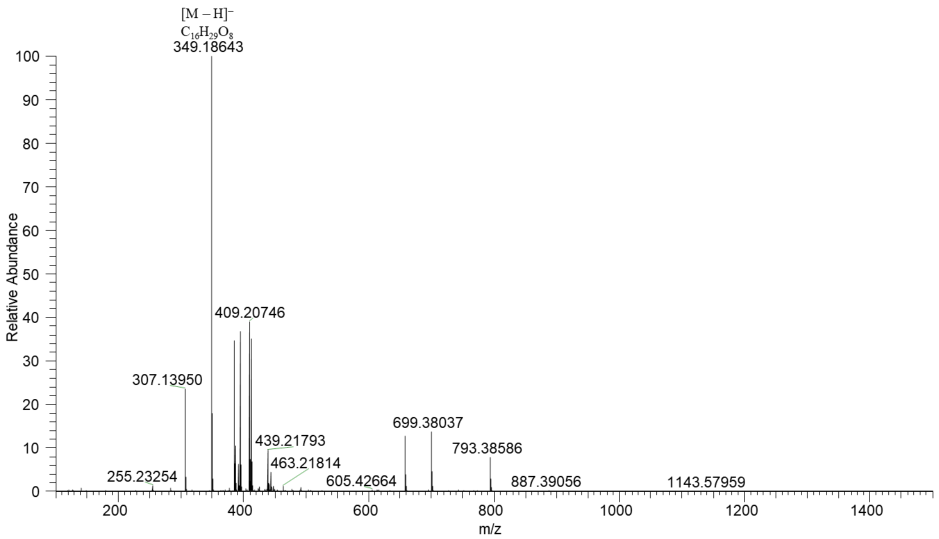

Figure S35 ESI-Q-Orbitrap-MS spectrum of **4**

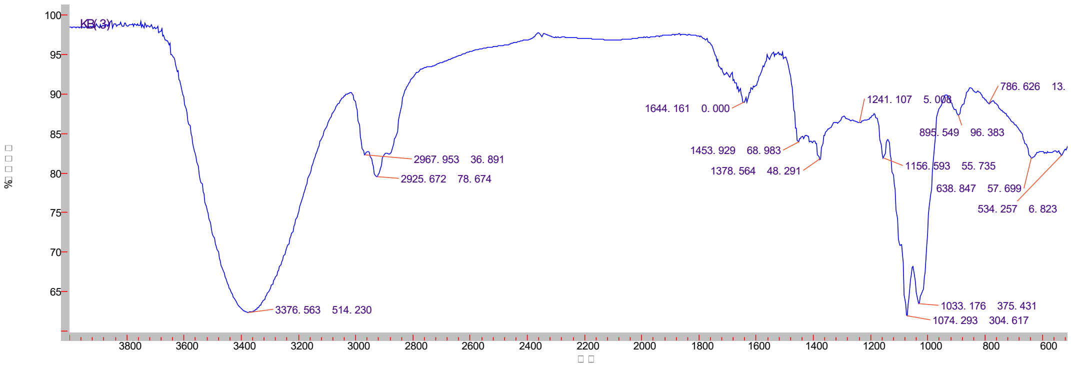

Figure S36 IR spectrum of **4**

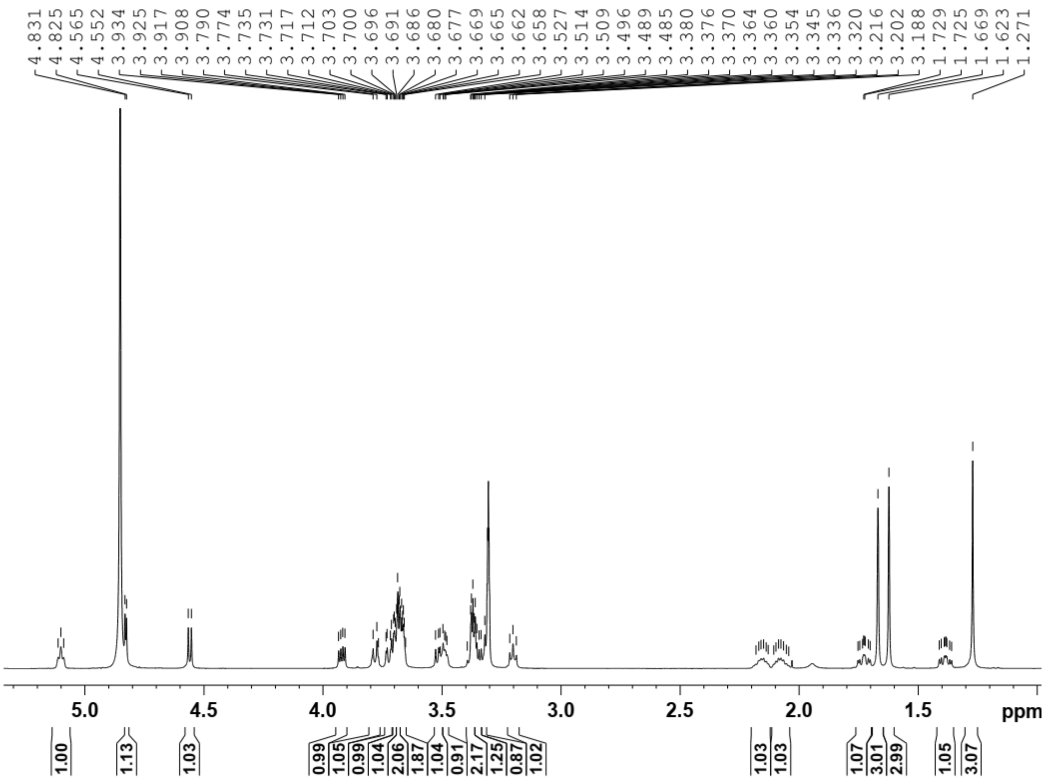

Figure S37 ^{1}H NMR (600 MHz, CD_3OD) spectrum of **5**

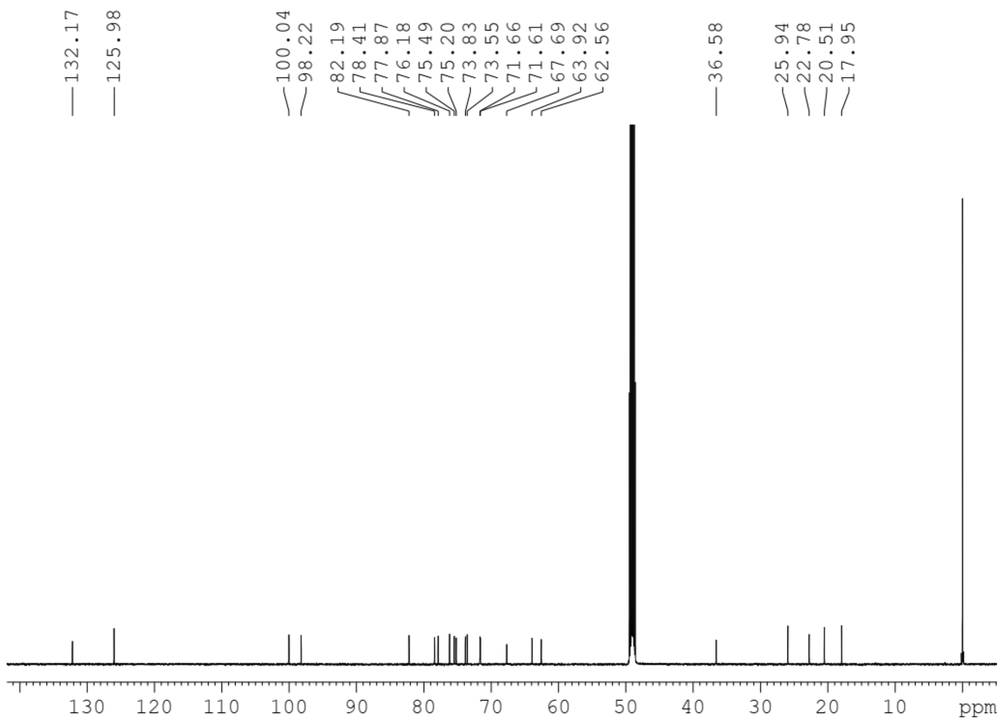

Figure S38 ^{13}C NMR (150 MHz, CD_3OD) spectrum of **5**

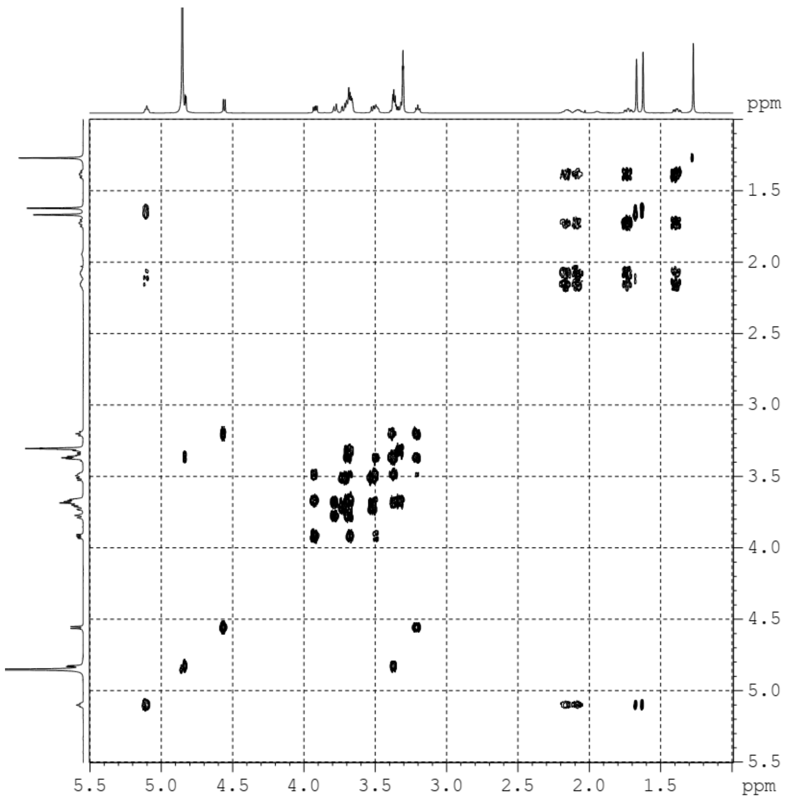

Figure S39 ^1H ^1H COSY (CD_3OD) spectrum of **5**

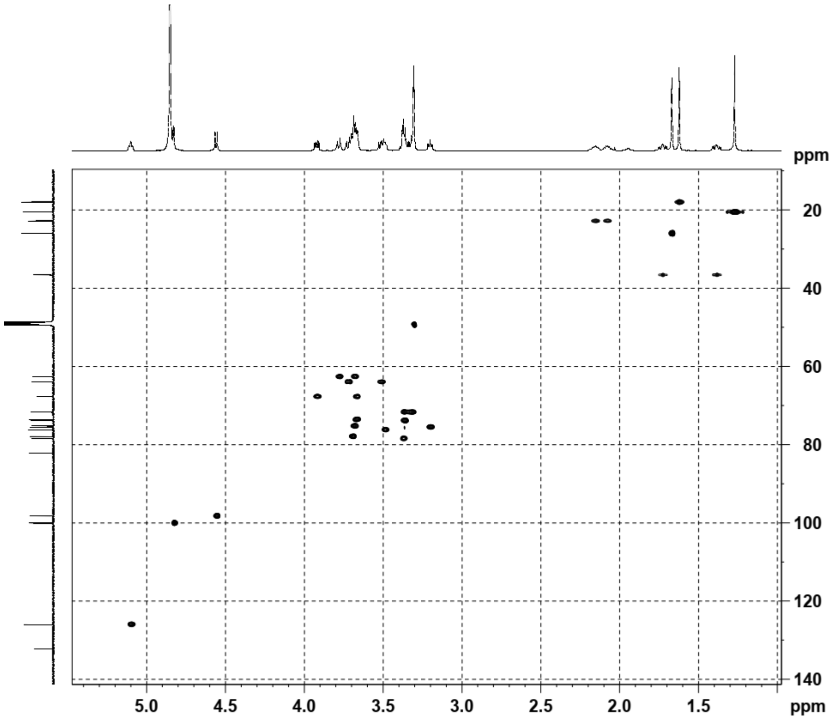

Figure S40 HSQC (CD_3OD) spectrum of **5**

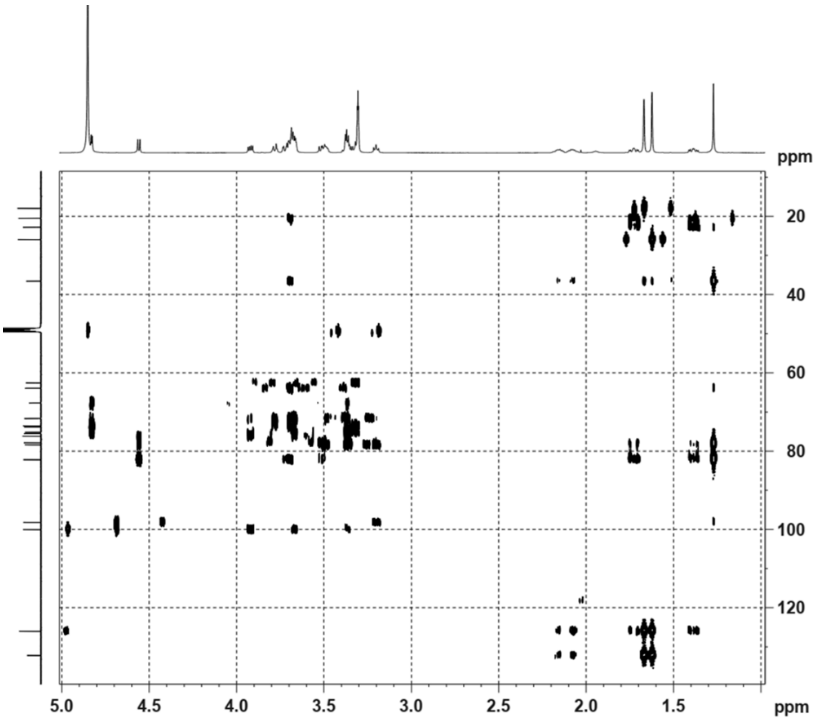

Figure S41 HMBC (CD_3OD) spectrum of **5**

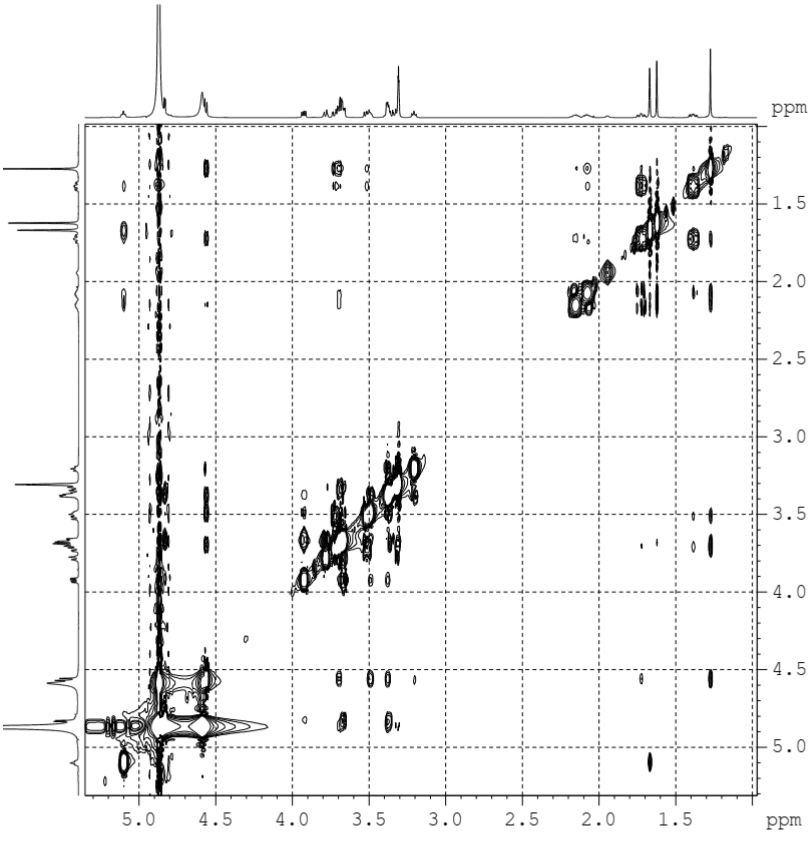

Figure S42 NOESY (CD_3OD) spectrum of **5**

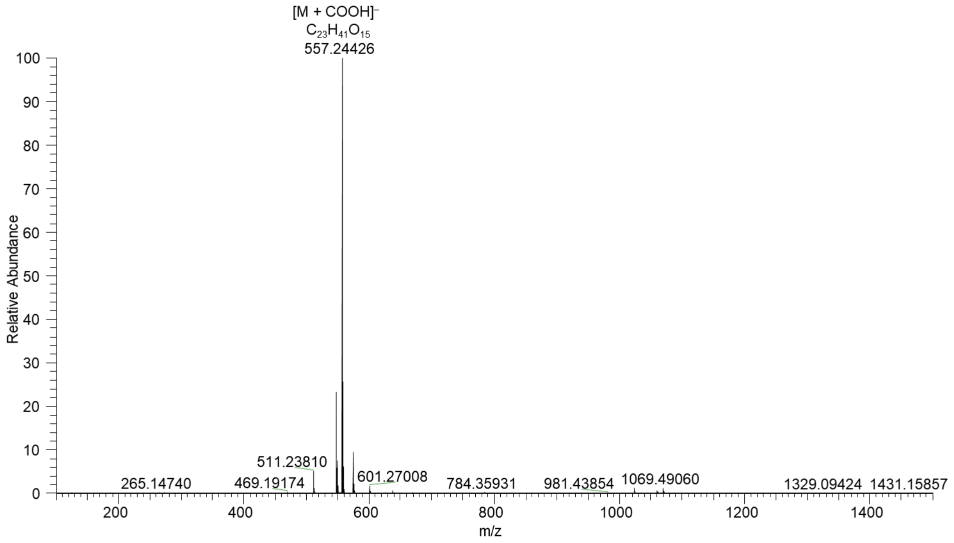

Figure S43 ESI-Q-Orbitrap-MS spectrum of **5**

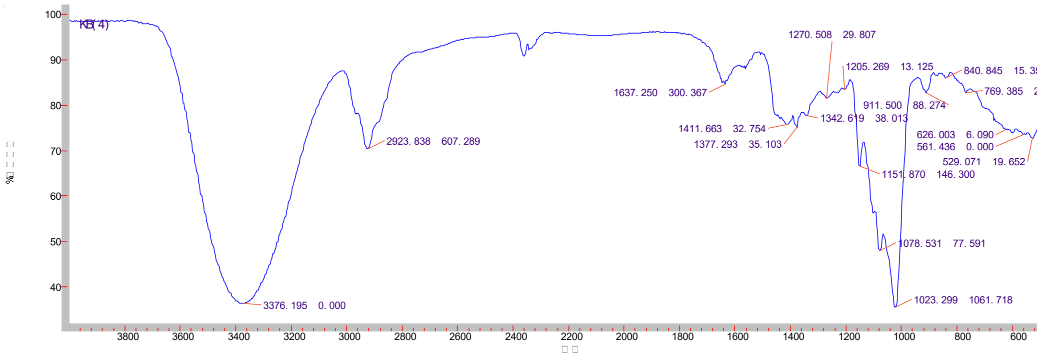

Figure S44 IR spectrum of **5**

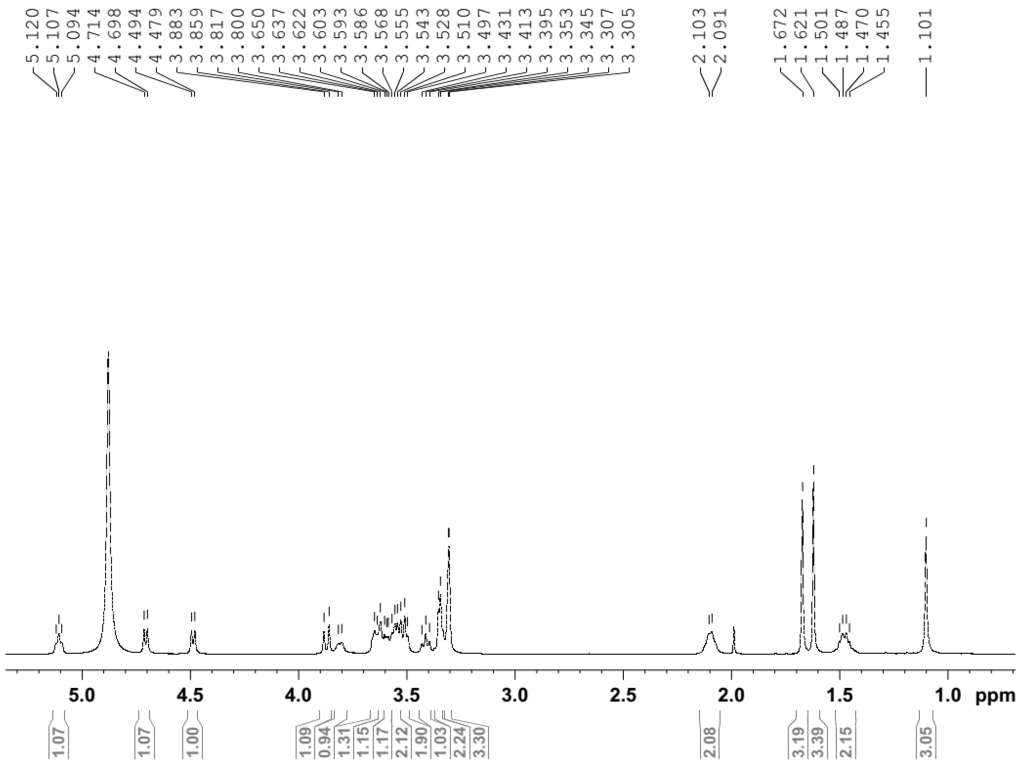

Figure S45 ^{1}H NMR (500 MHz, CD_3OD) spectrum of **6**

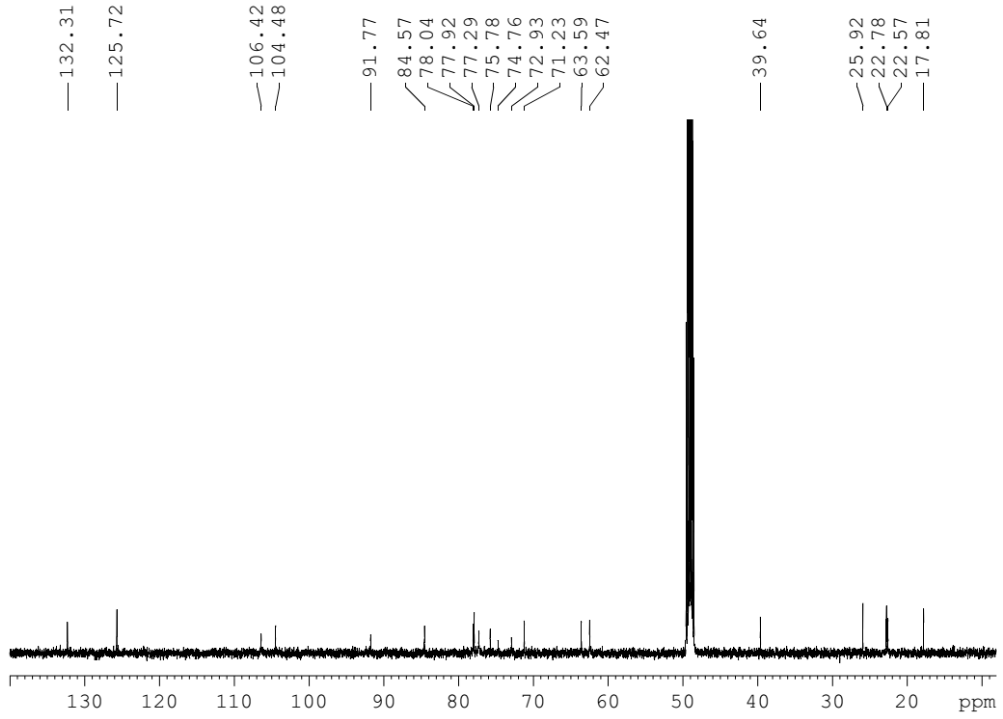

Figure S46 ^{13}C NMR (125 MHz, CD_3OD) spectrum of **6**

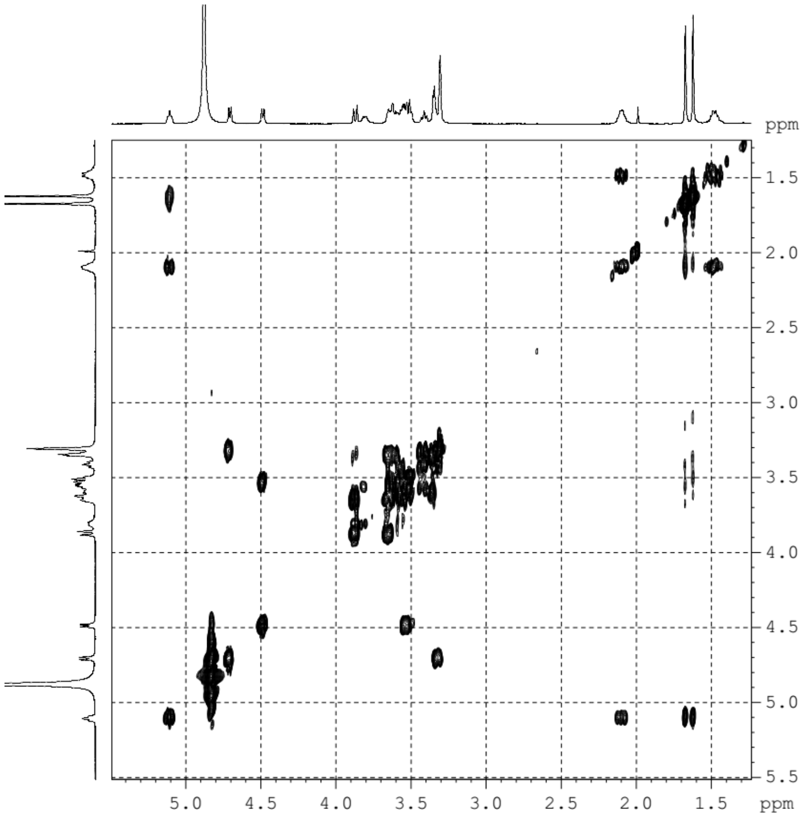

Figure S47 ^{1}H ^{1}H COSY (CD_3OD) spectrum of **6**

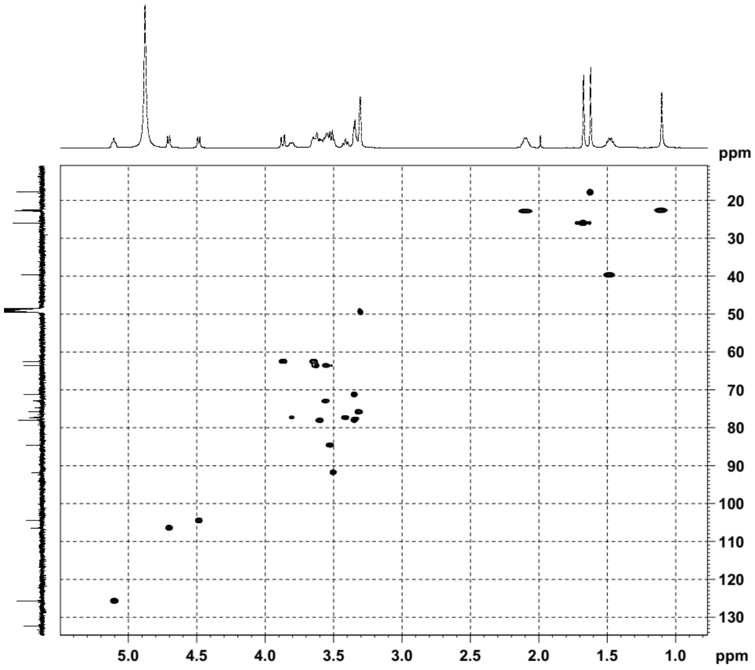

Figure S48 HSQC (CD_3OD) spectrum of **6**

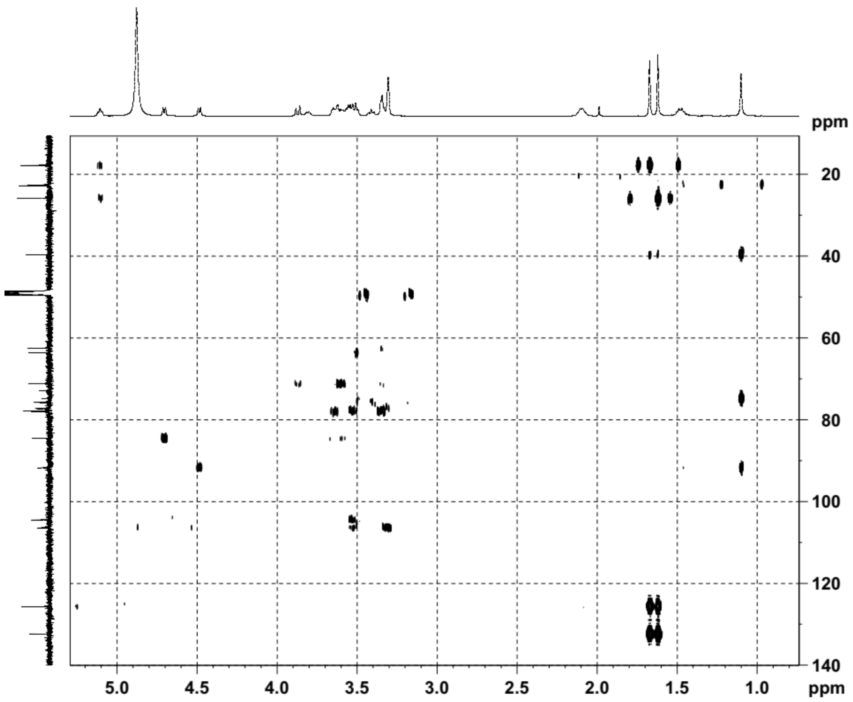

Figure S49 HMBC (CD_3OD) spectrum of **6**

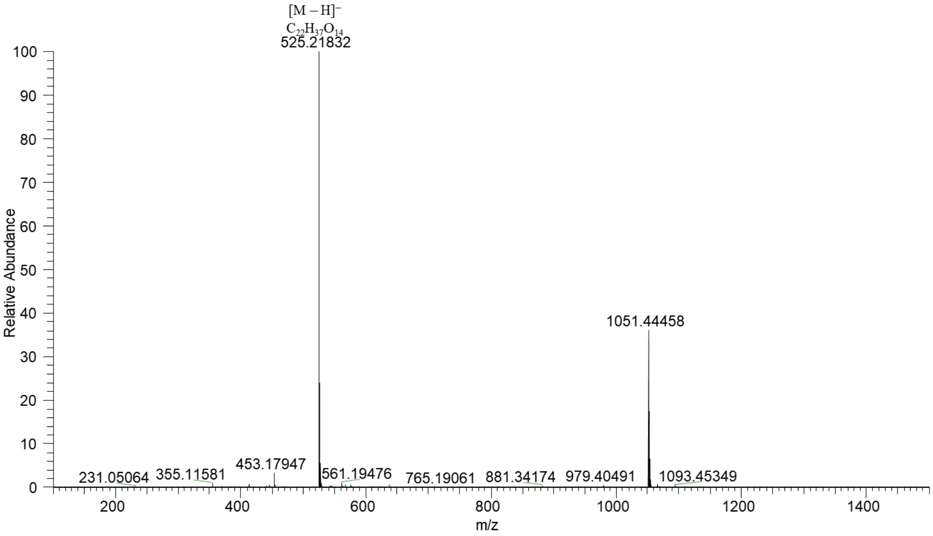

Figure S50 ESI-Q-Orbitrap-MS spectrum of **6**

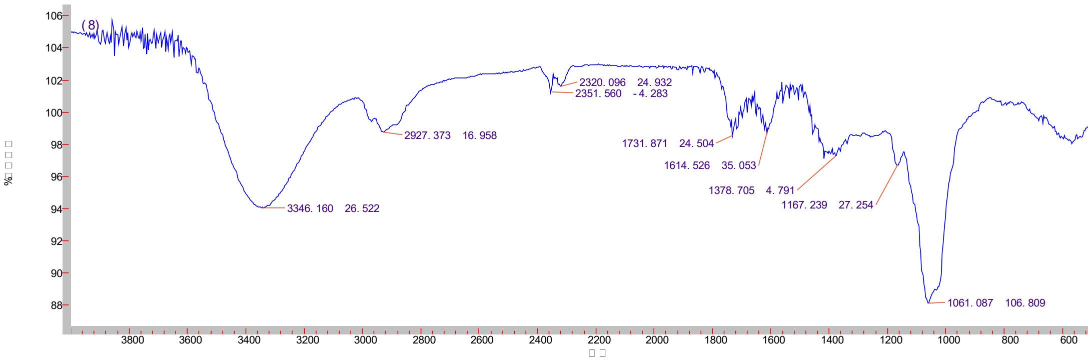

Figure S51 IR spectrum of **6**

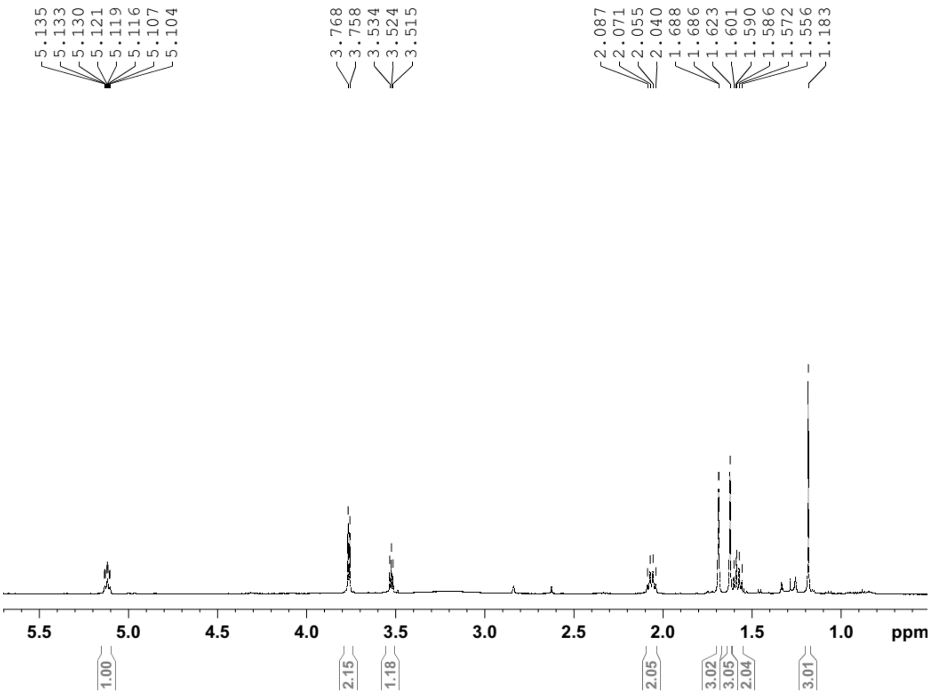

Figure S52 ^{1}H NMR (500 MHz, $CDCl_3$) spectrum of **6a**

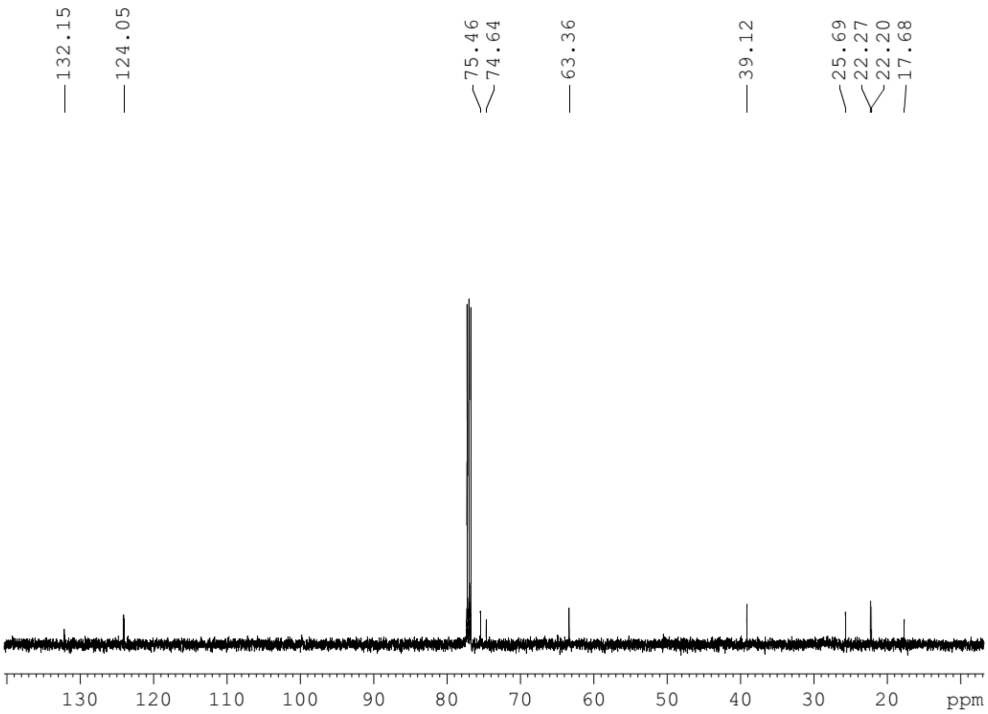

Figure S53 ^{13}C NMR (125 MHz, $CDCl_3$) spectrum of **6a**

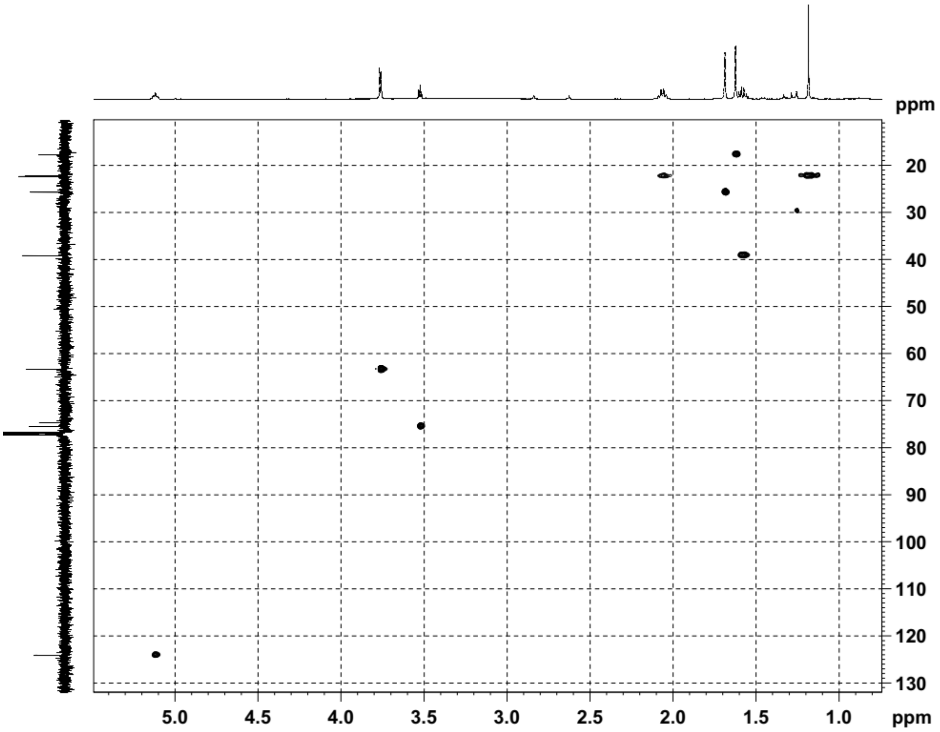

Figure S54 HSQC ($CDCl_3$) spectrum of **6a**

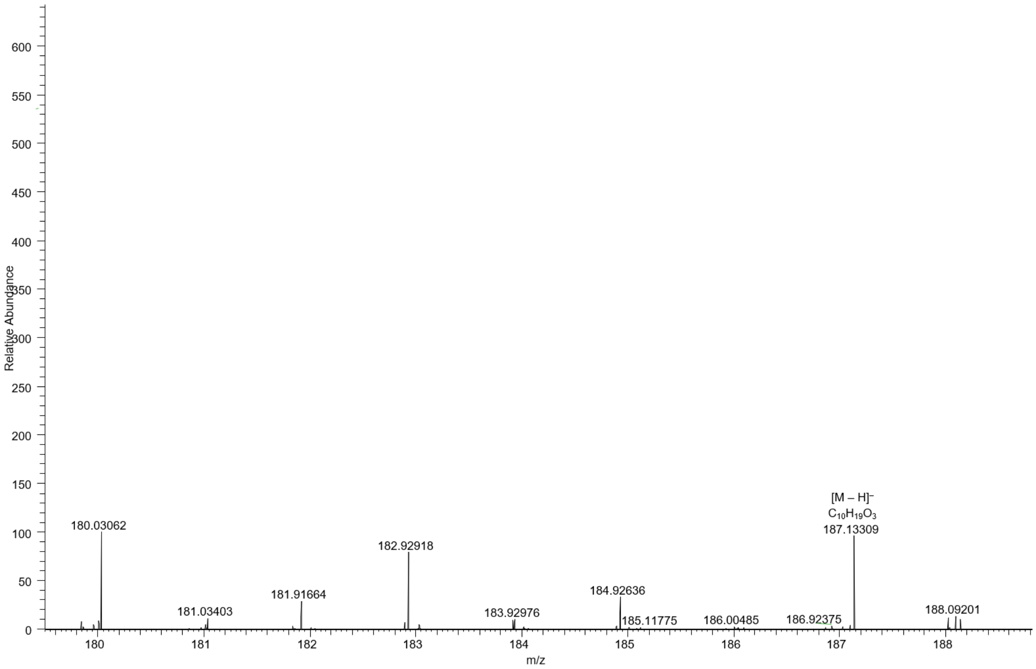

Figure S55 ESI-Q-Orbitrap-MS spectrum of **6a**

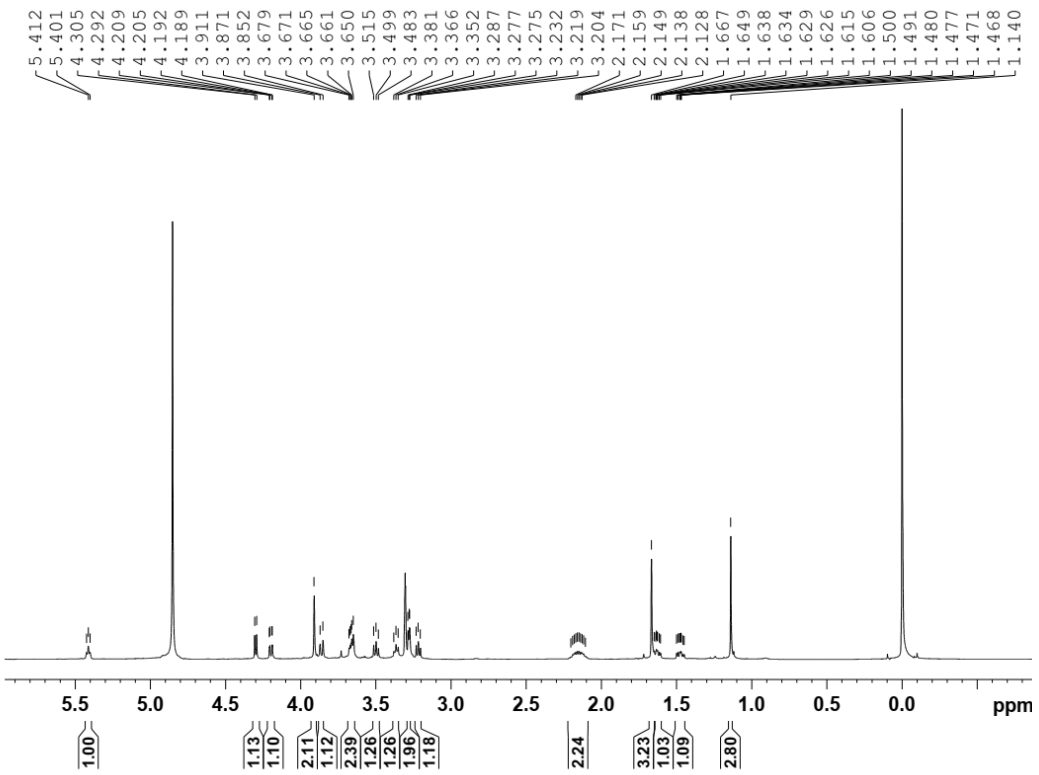

Figure S56 ^{1}H NMR (600 MHz, CD_3OD) spectrum of **7**

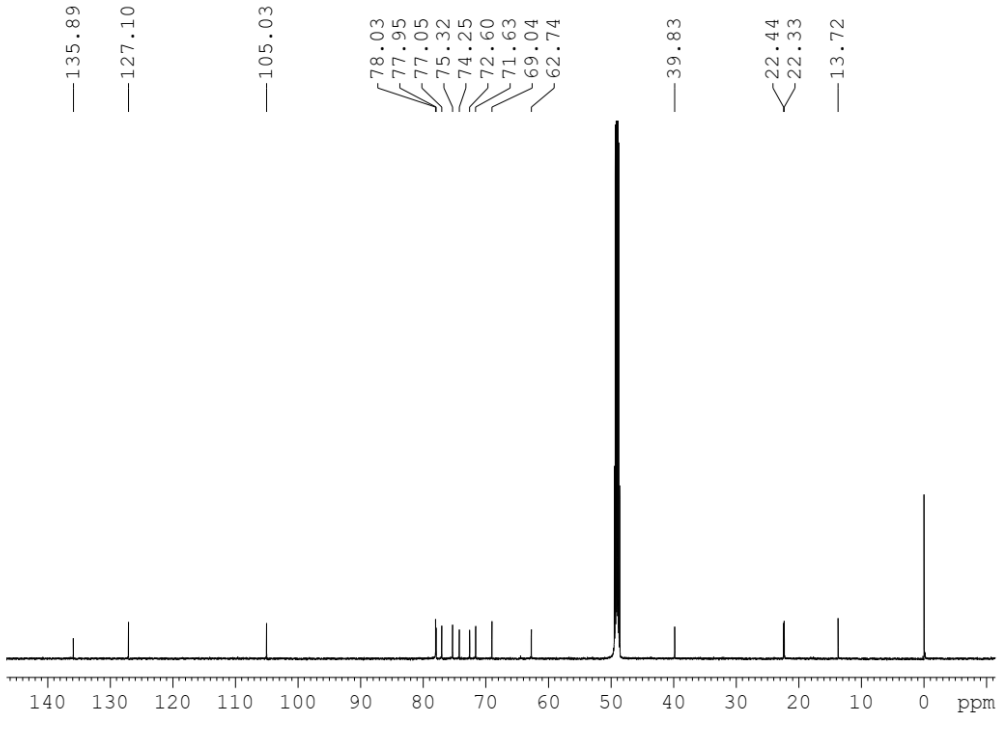

Figure S57 ^{13}C NMR (150 MHz, CD_3OD) spectrum of **7**

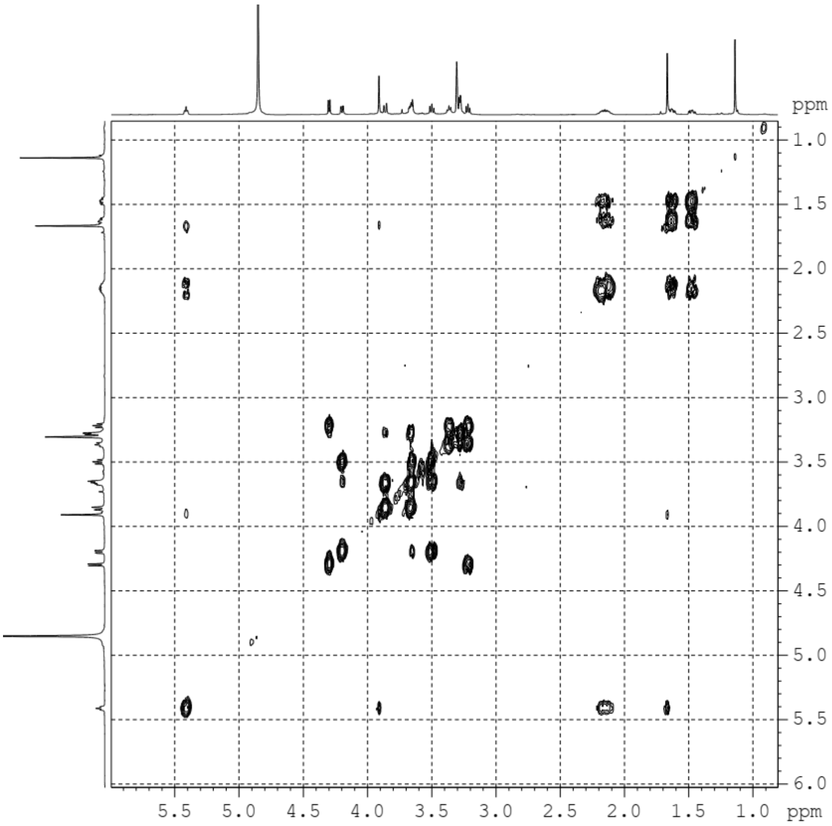

Figure S58 ^{1}H ^{1}H COSY (CD_3OD) spectrum of **7**

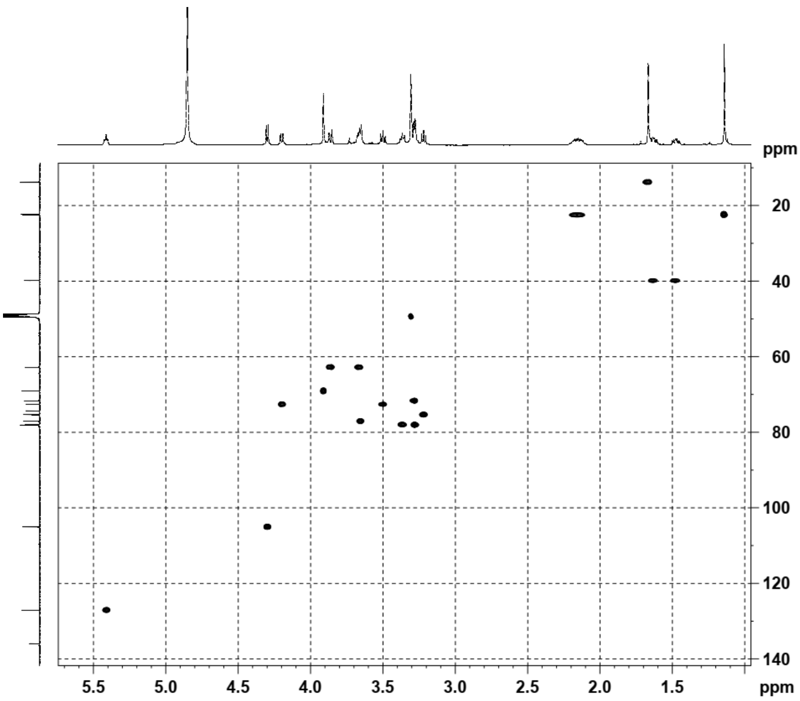

Figure S59 HSQC (CD_3OD) spectrum of **7**

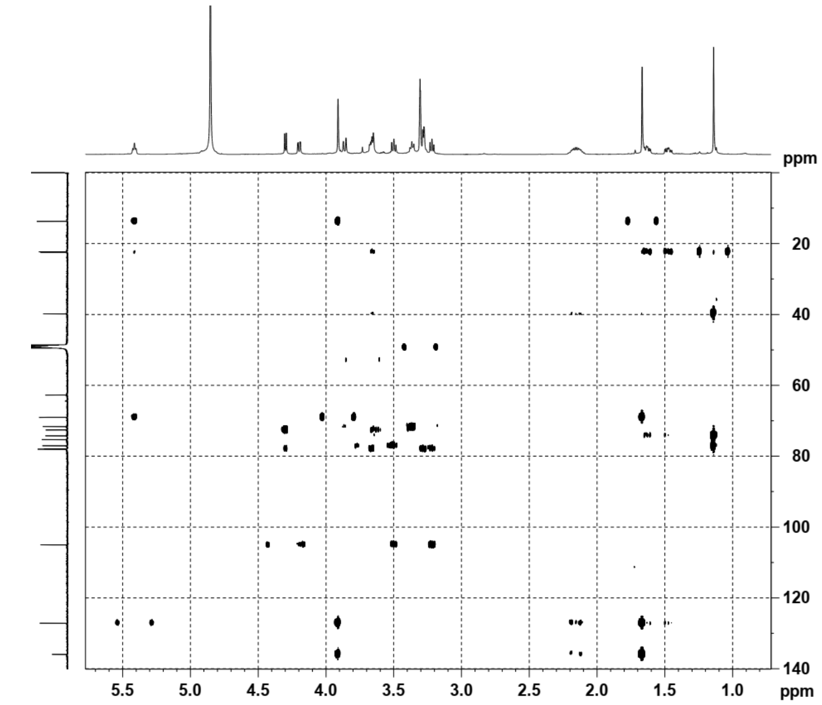

Figure S60 HMBC (CD_3OD) spectrum of **7**

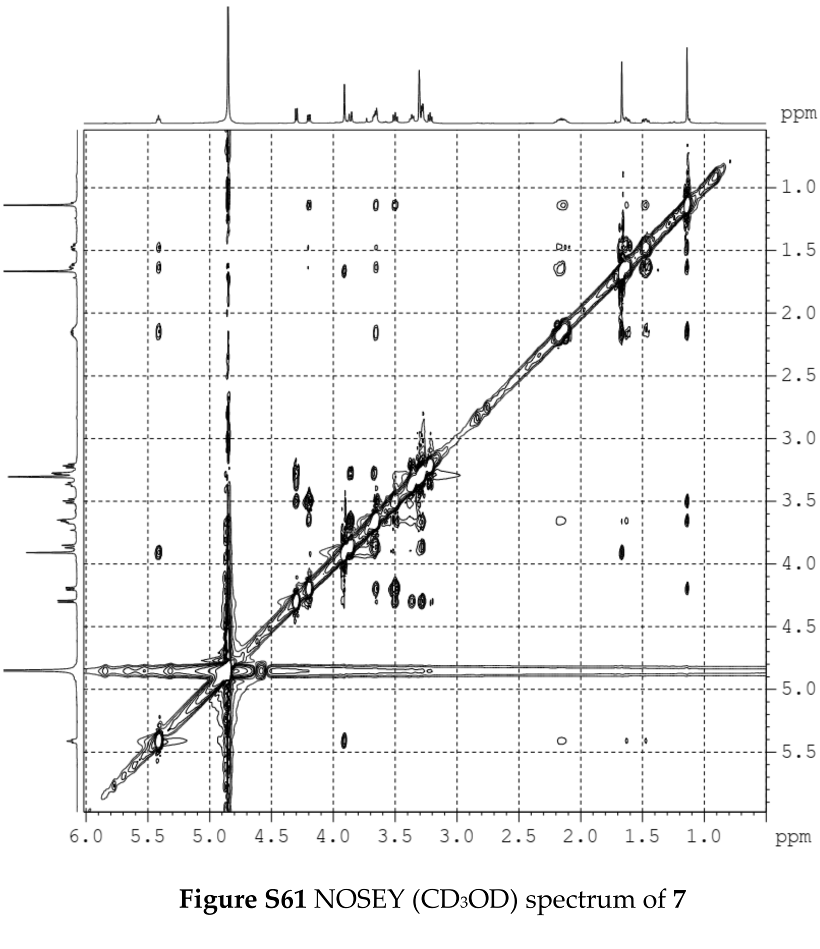

Figure S61 NOSEY (CD_3OD) spectrum of **7**

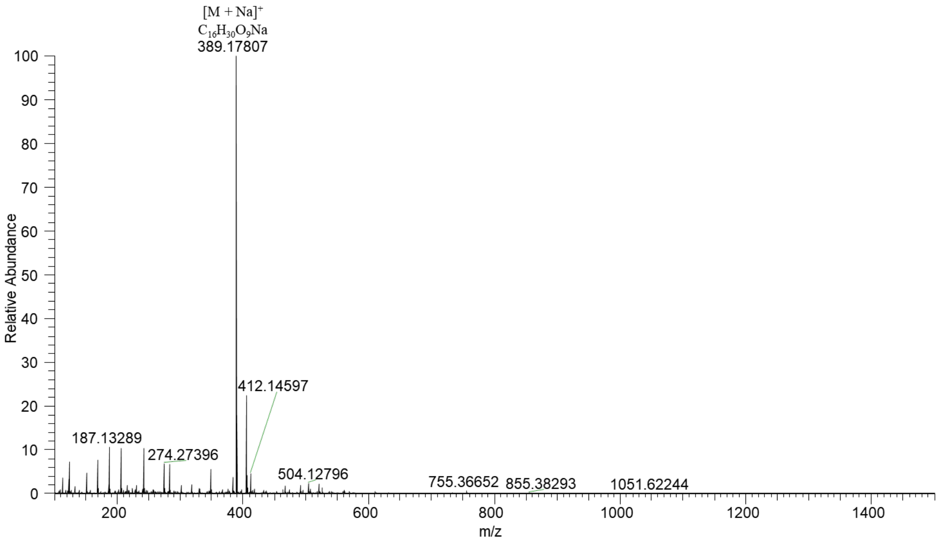

Figure S62 ESI-Q-Orbitrap-MS spectrum of **7**

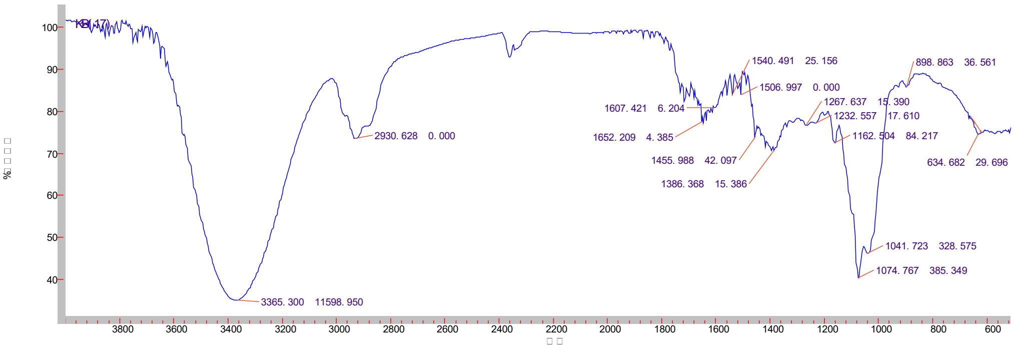

Figure S63 IR spectrum of **7**

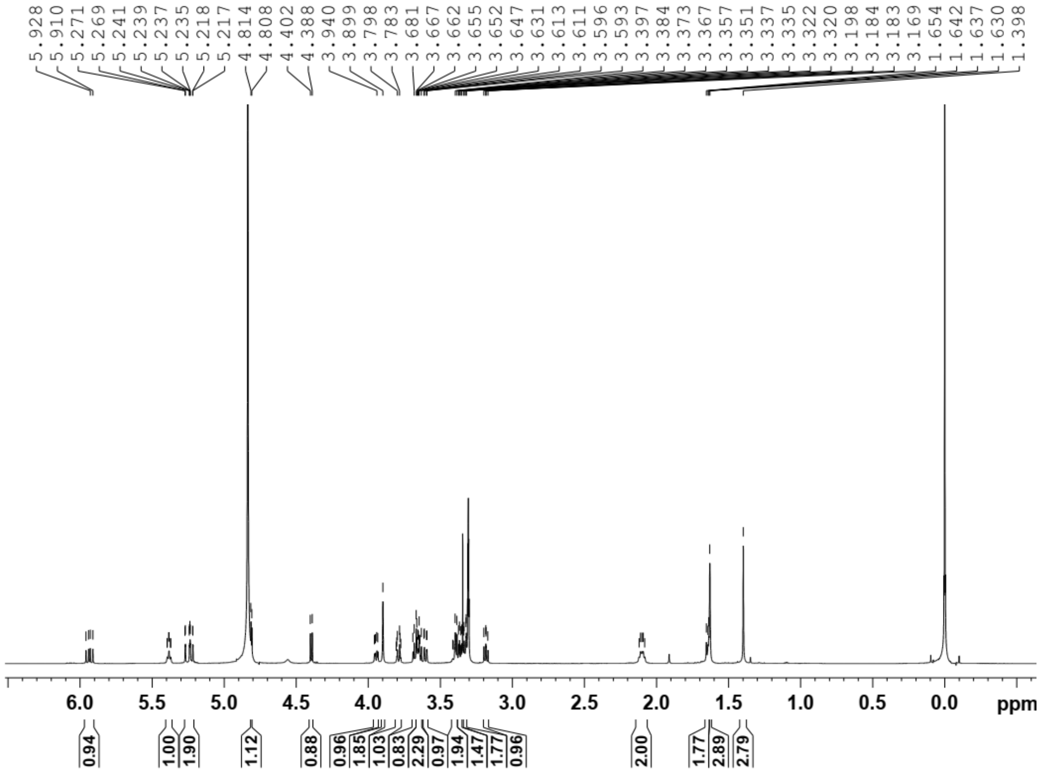

Figure S64 ^{1}H NMR (600 MHz, CD_3OD) spectrum of **8**

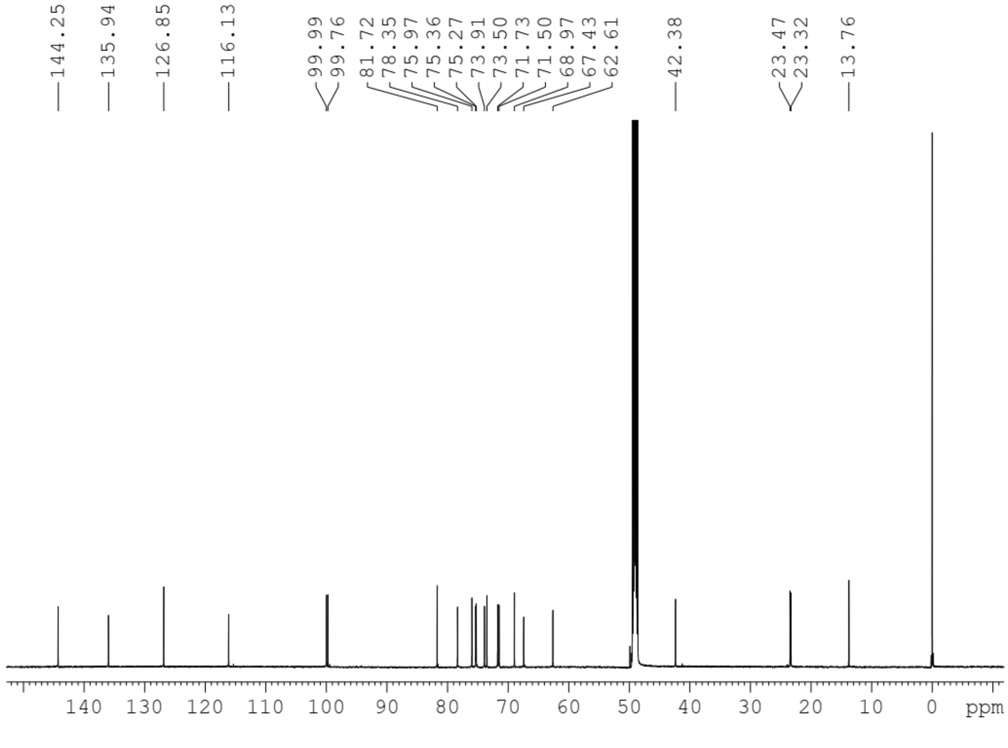

Figure S65 ^{13}C NMR (150 MHz, CD_3OD) spectrum of **8**

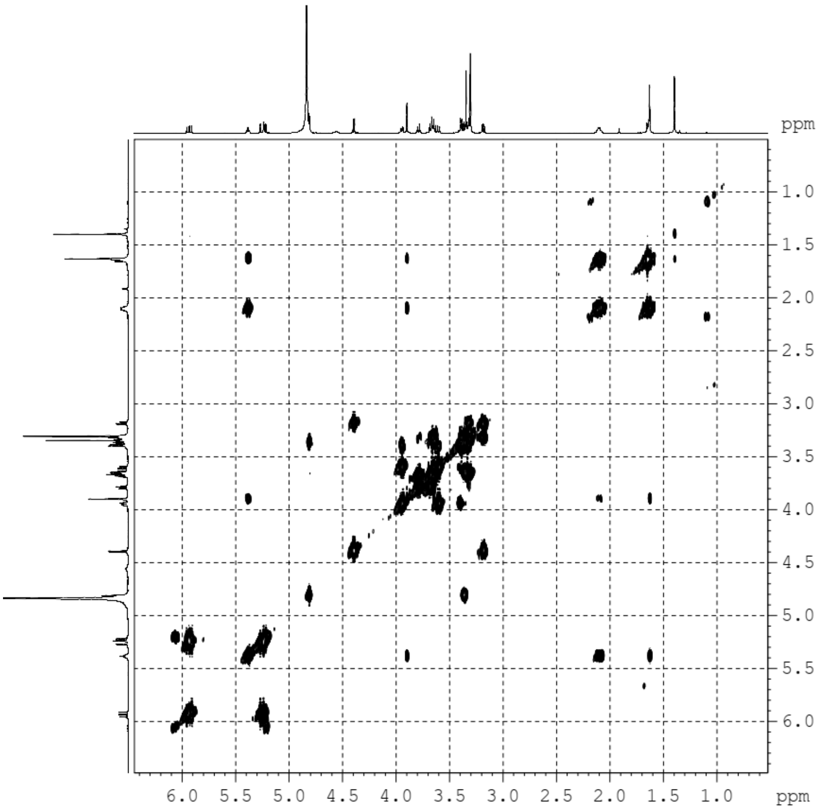

Figure S66 ^{1}H ^{1}H COSY (CD_3OD) spectrum of **8**

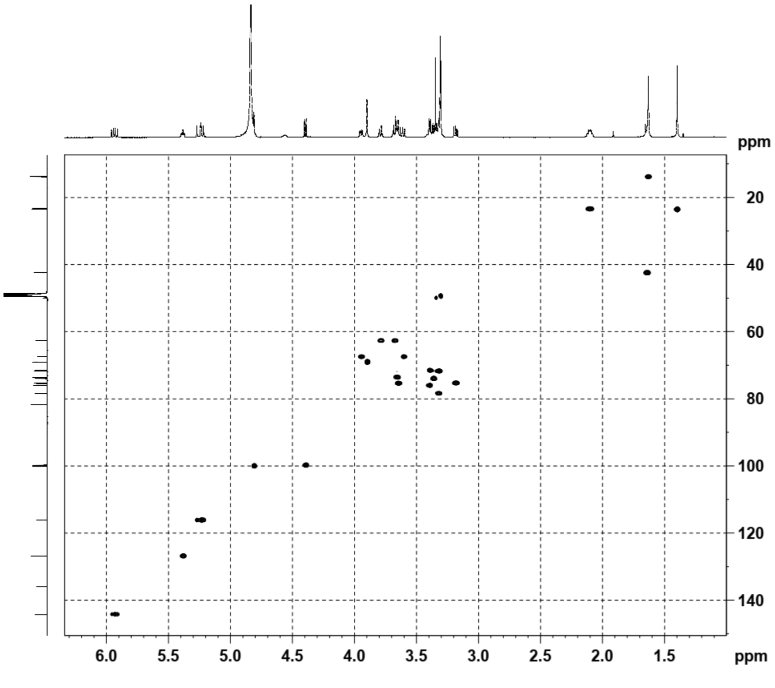

Figure S67 HSQC (CD_3OD) spectrum of **8**

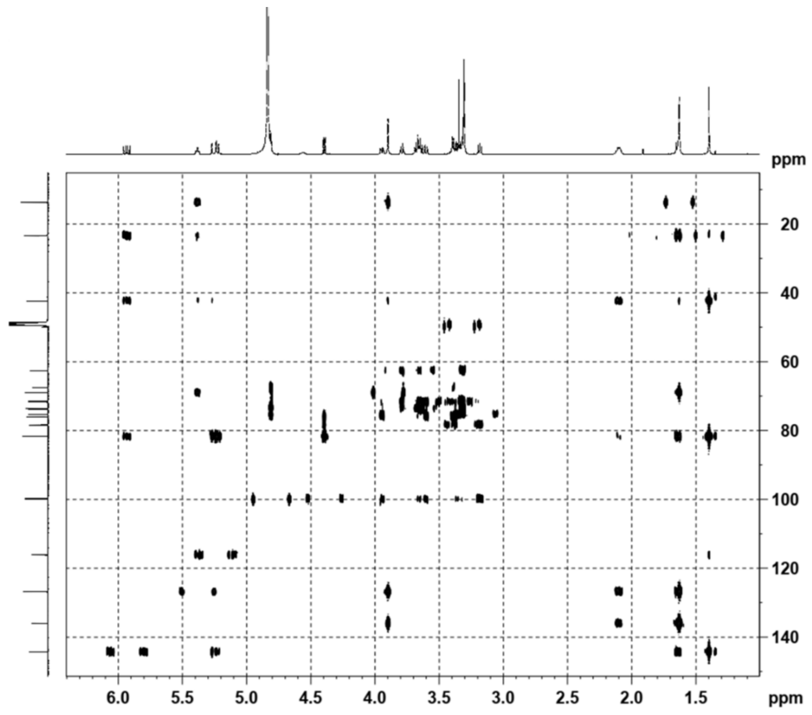

Figure S68 HMBC (CD_3OD) spectrum of **8**

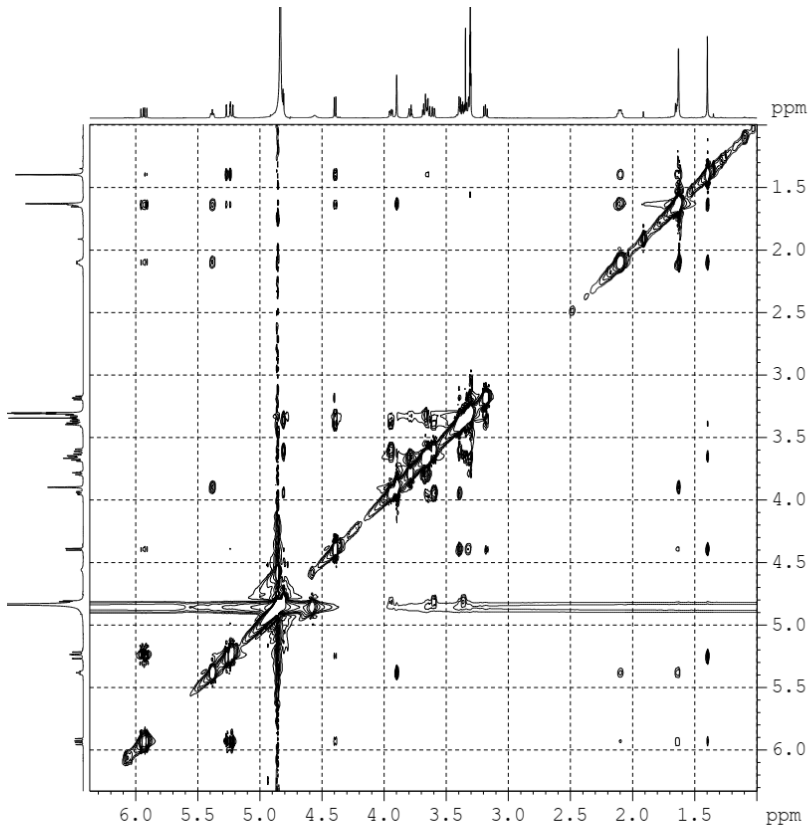

Figure S69 NOESY (CD_3OD) spectrum of **8**

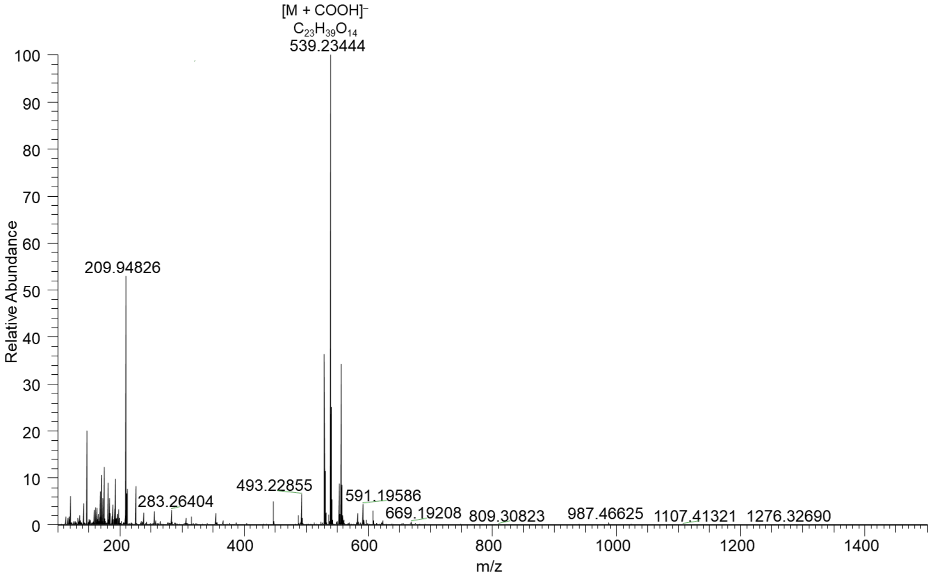

Figure S70 ESI-Q-Orbitrap-MS spectrum of **8**

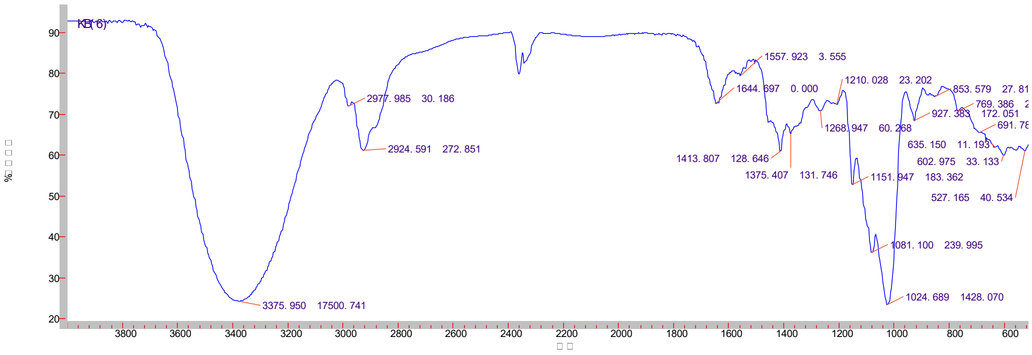

Figure S71 IR spectrum of **8**

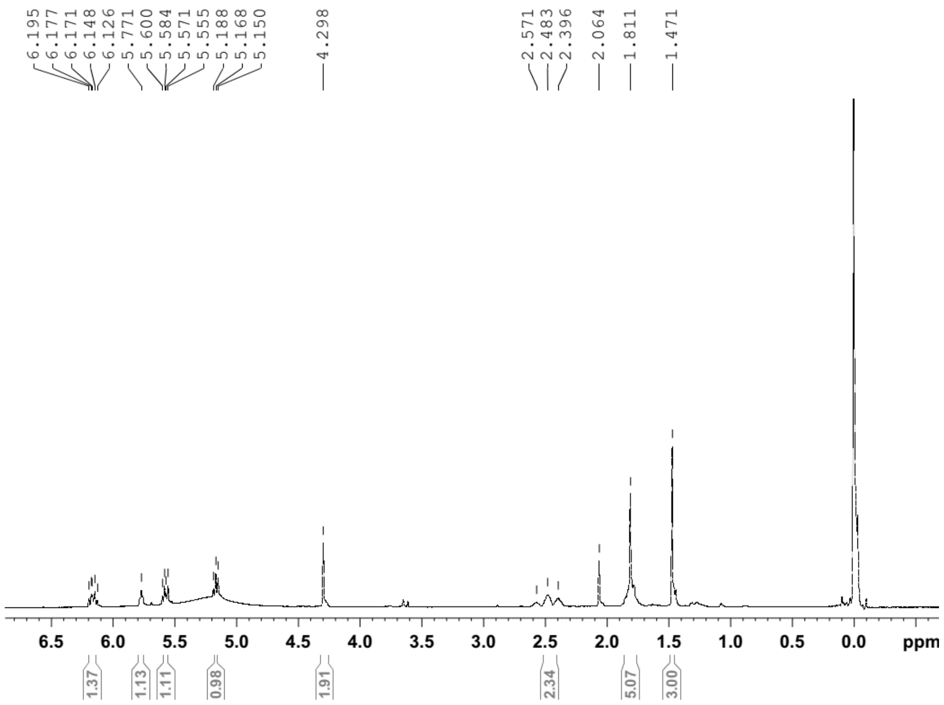

Figure S72 ^{1}H NMR (600 MHz, C_5D_5N) spectrum of **8a**

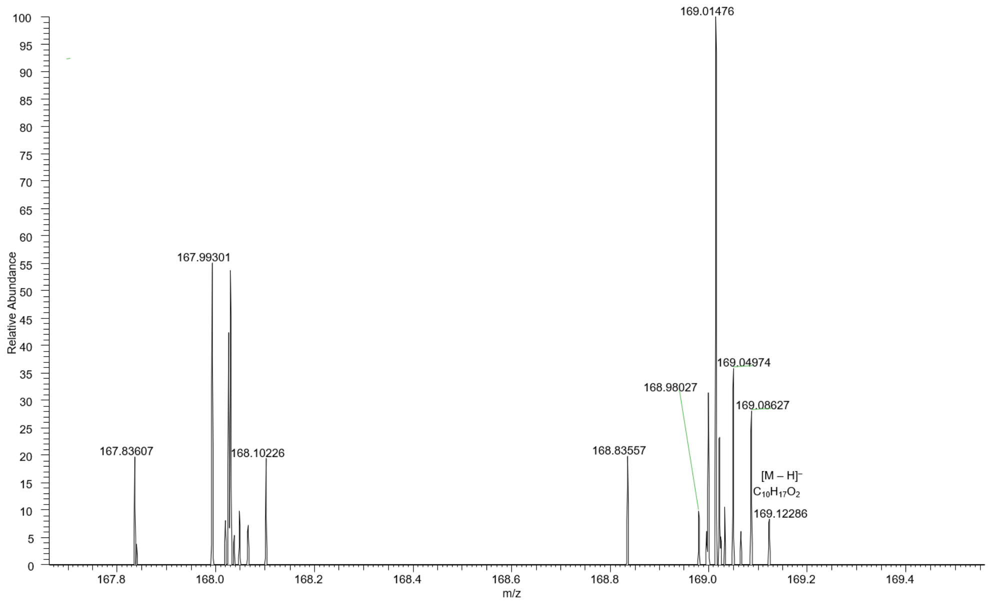

Figure S73 ESI-Q-Orbitrap-MS spectrum of **8a**

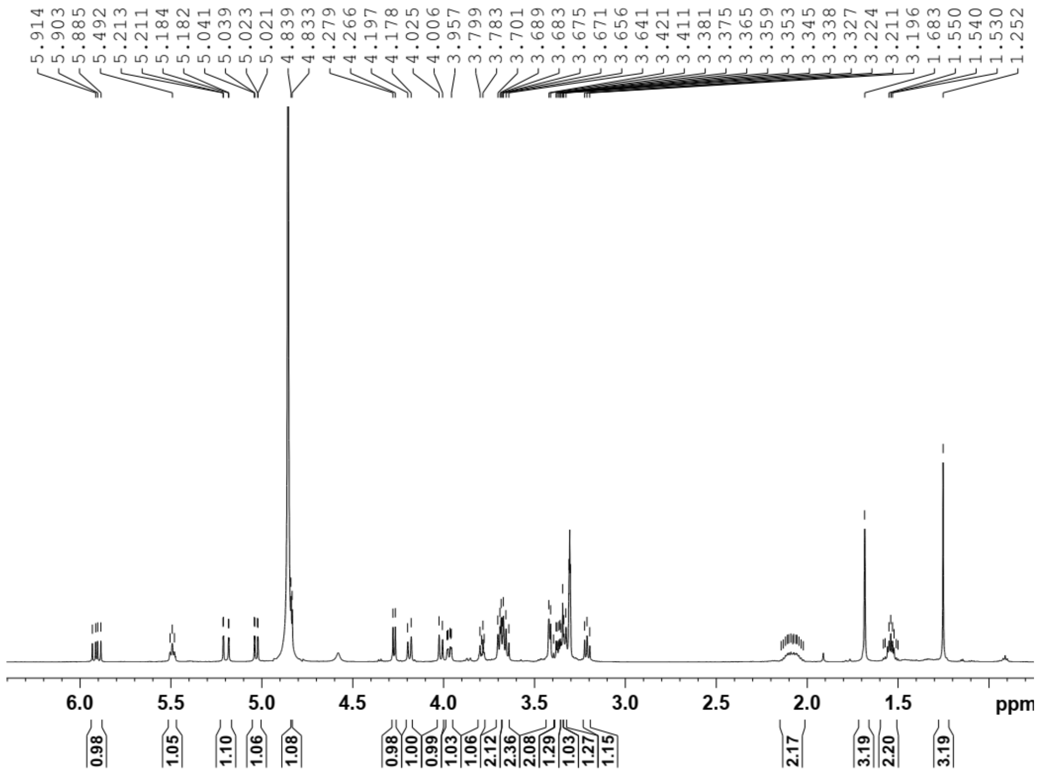

Figure S74 ^{1}H NMR (600 MHz, CD_3OD) spectrum of **9**

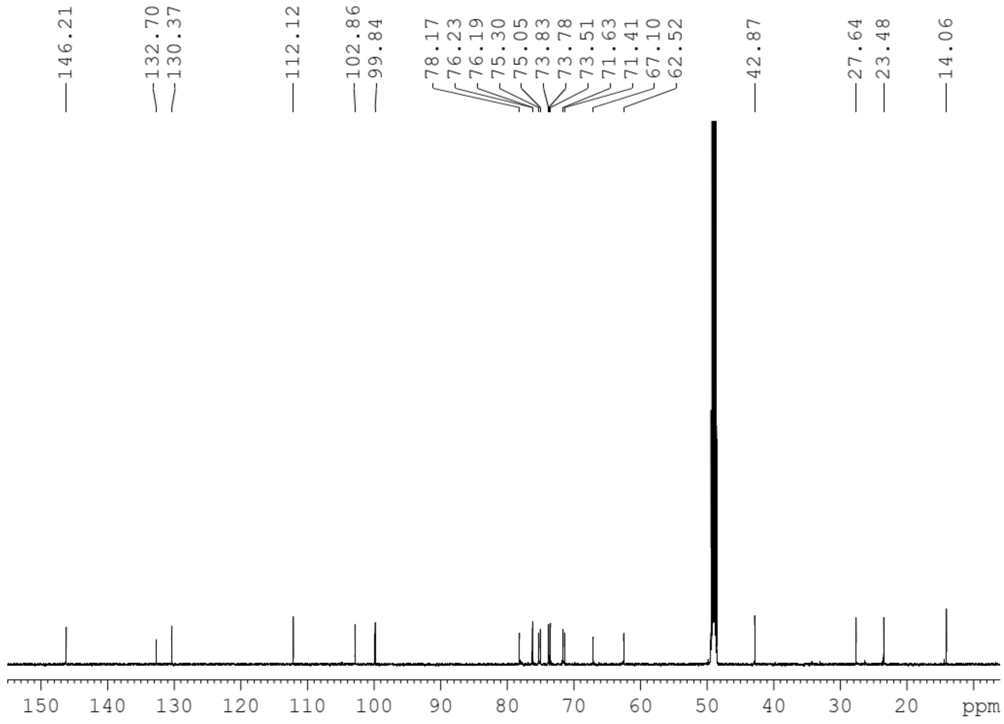

Figure S75 ^{13}C NMR (150 MHz, CD_3OD) spectrum of **9**

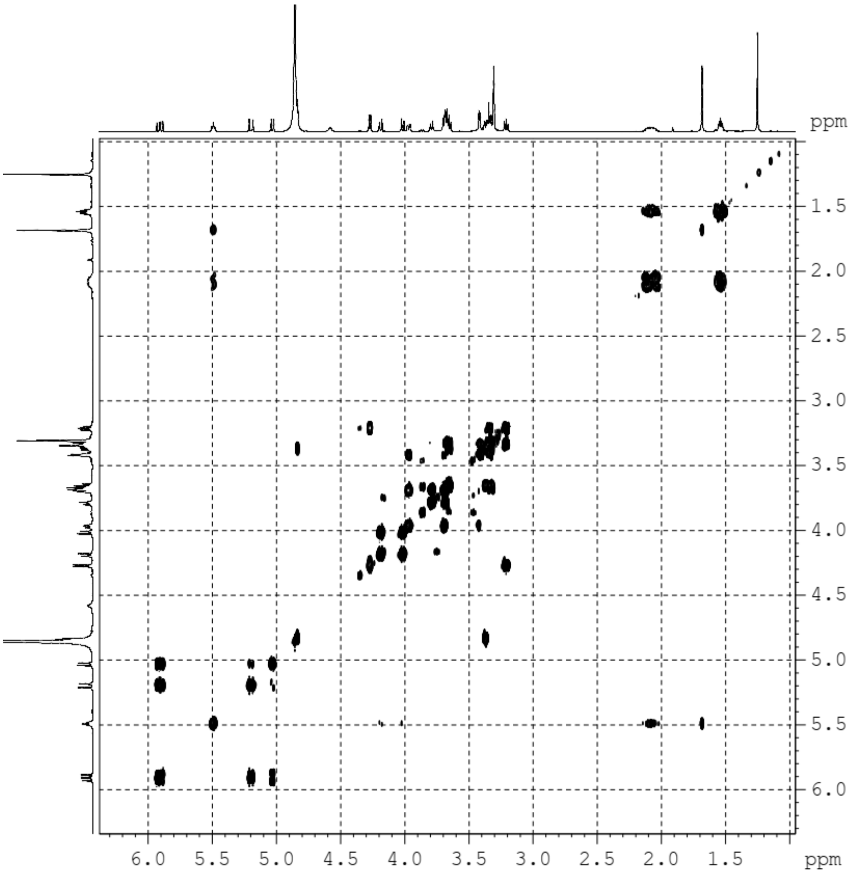

Figure S76 ^1H ^1H COSY (CD_3OD) spectrum of **9**

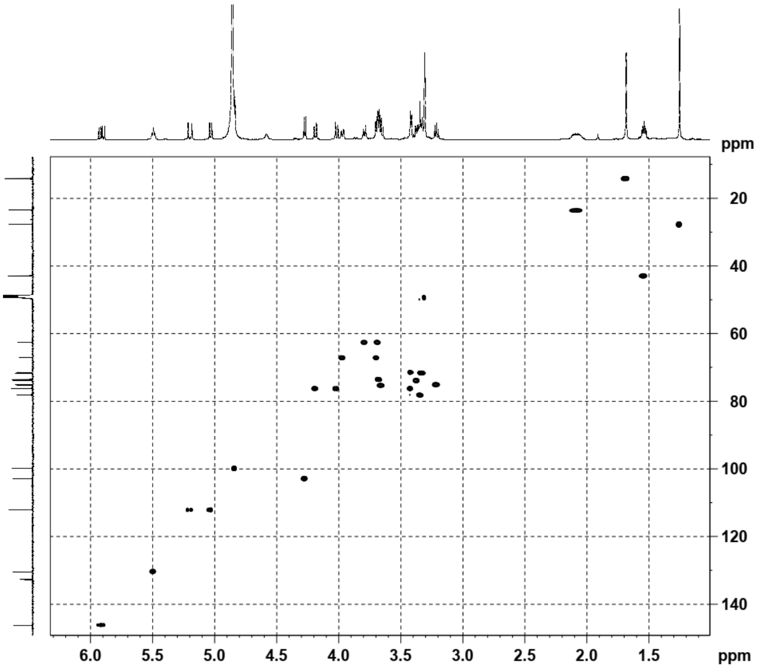

Figure S77 HSQC (CD_3OD) spectrum of **9**

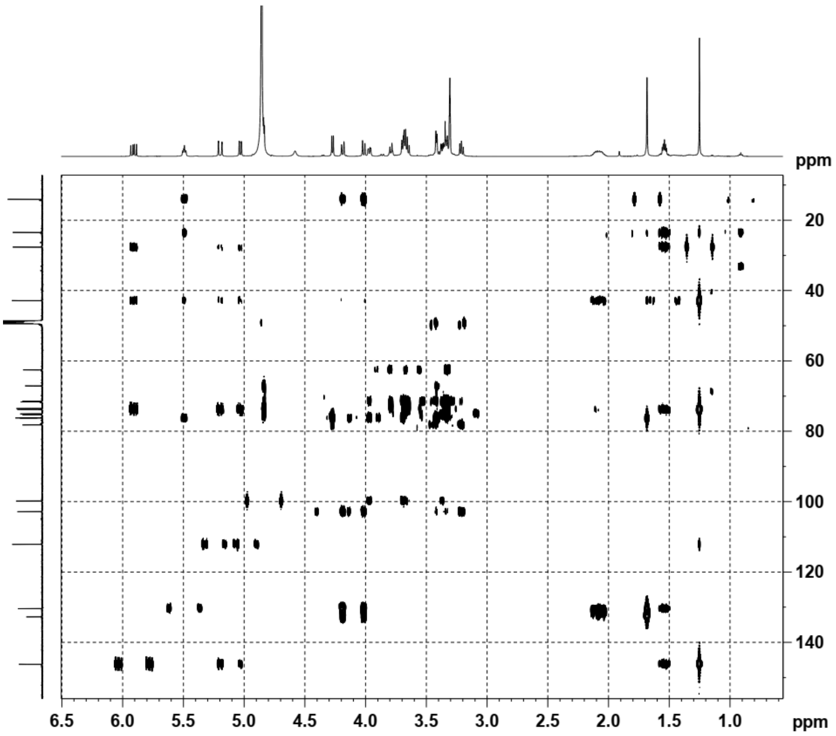

Figure S78 HMBC (CD_3OD) spectrum of **9**

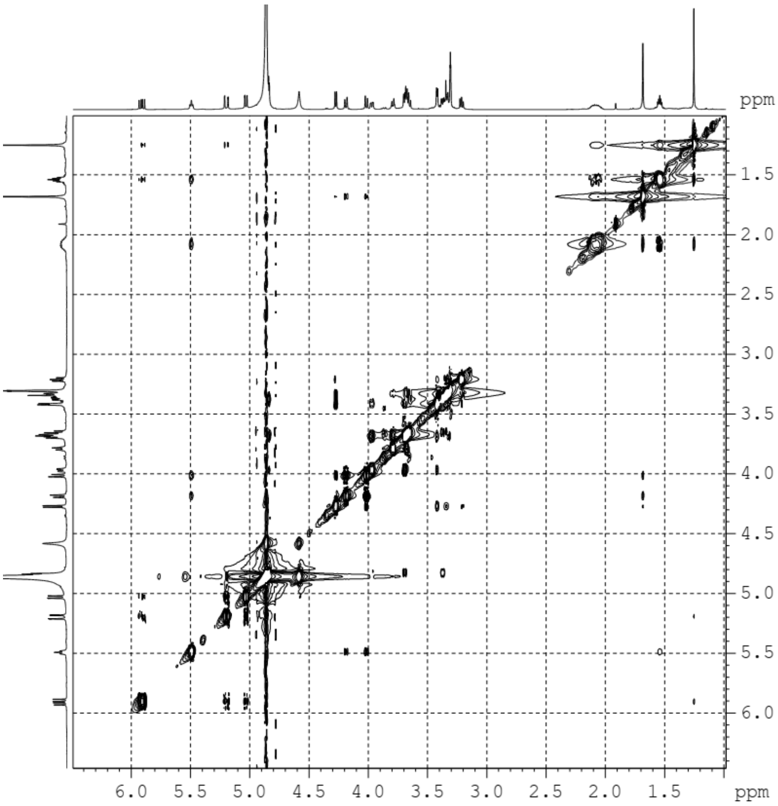

Figure S79 NOESY (CD_3OD) spectrum of **9**

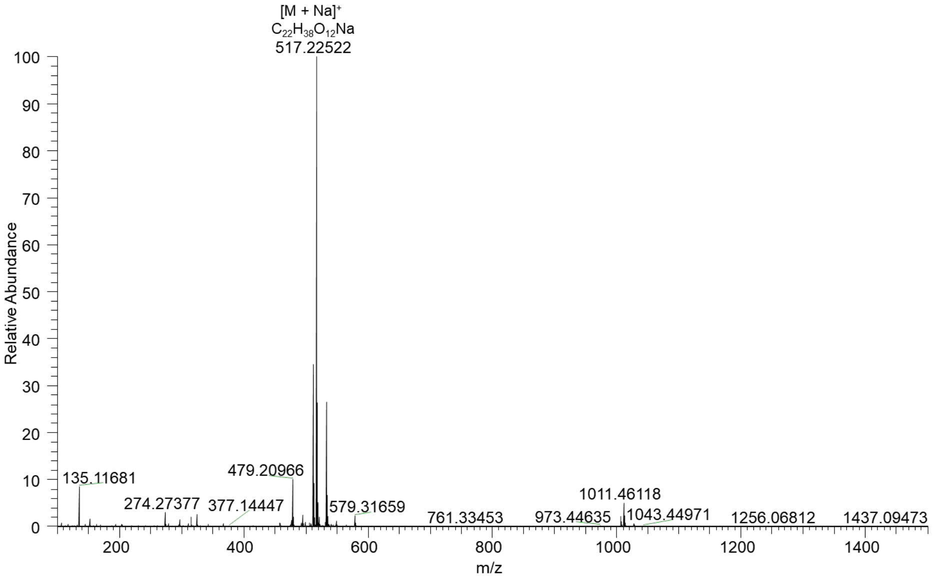

Figure S80 ESI-Q-Orbitrap-MS spectrum of **9**

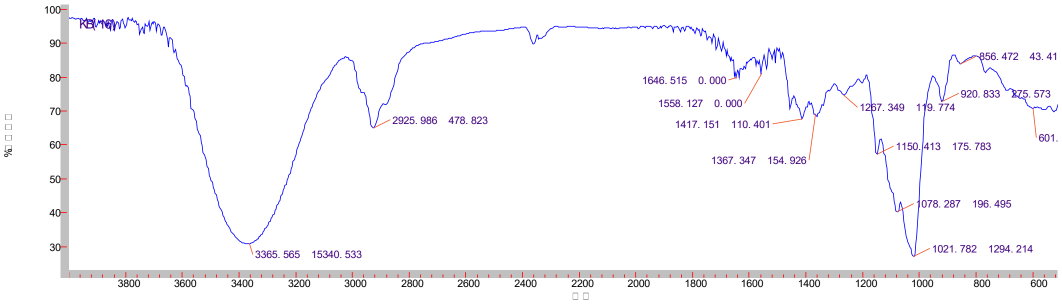

Figure S81 IR spectrum of **9**

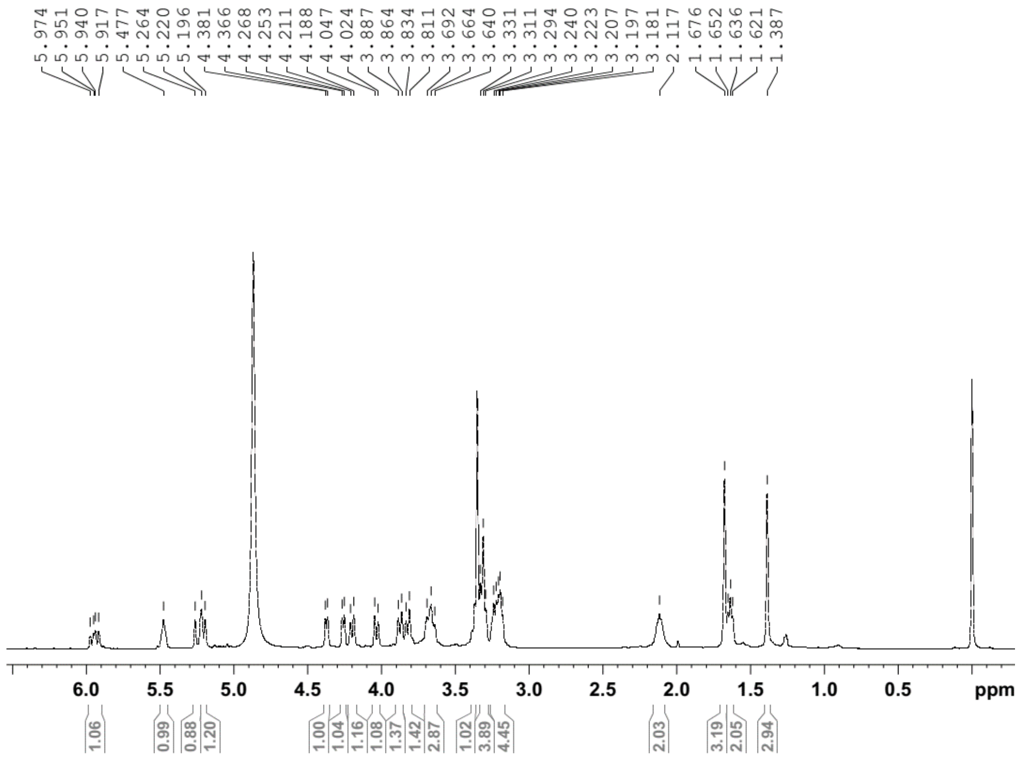

Figure S82 ^{1}H NMR (500 MHz, CD_3OD) spectrum of **10**

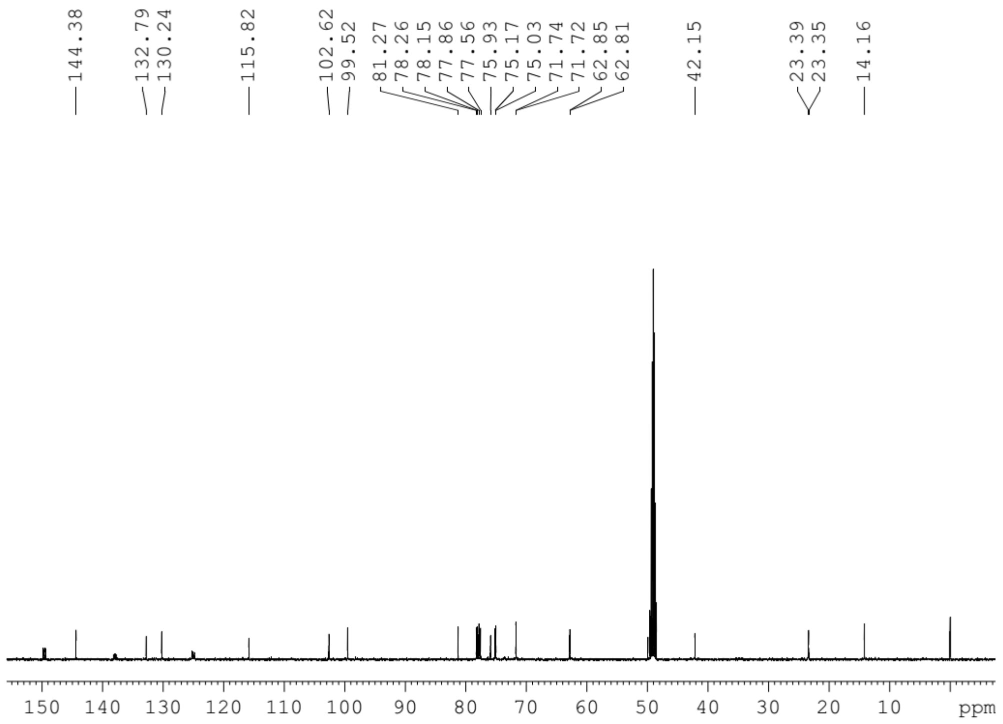

Figure S83 ^{13}C NMR (125 MHz, CD_3OD) spectrum of **10**

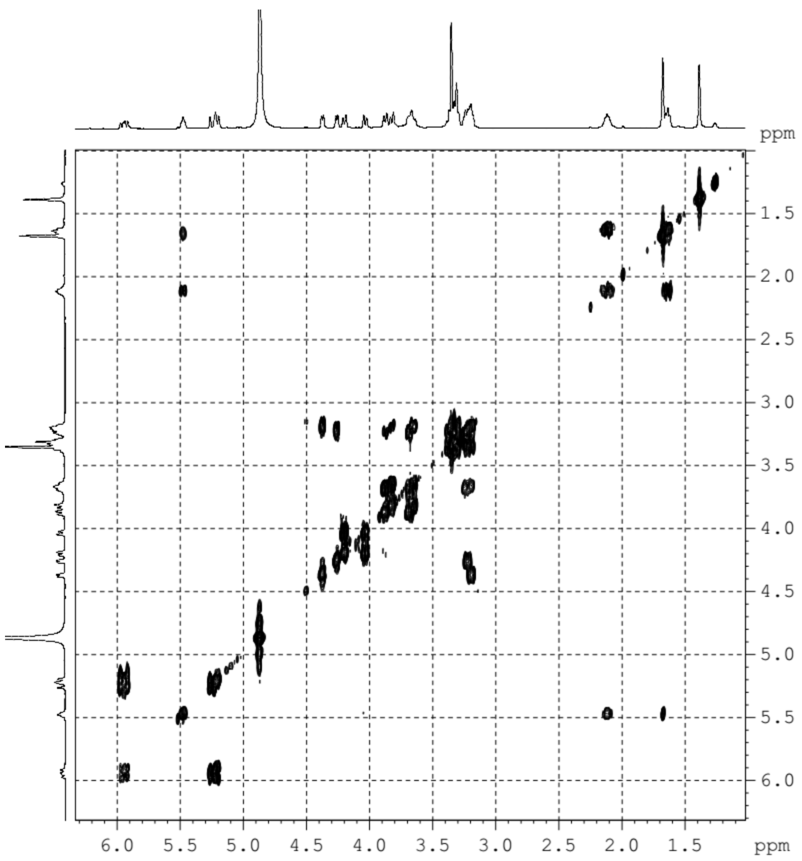

Figure S84 ^{1}H ^{1}H COSY (CD_3OD) spectrum of **10**

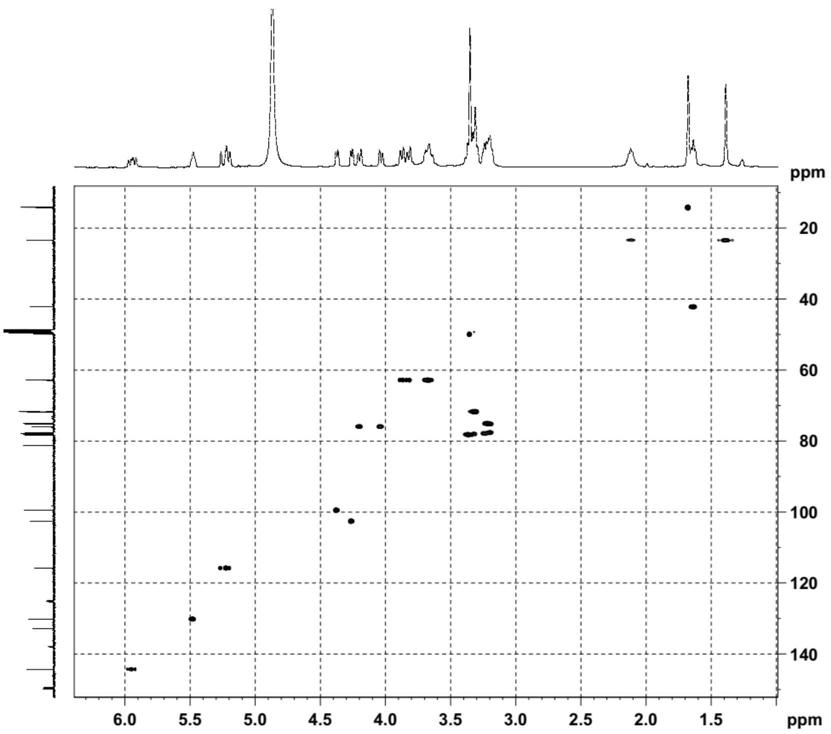

Figure S85 HSQC (CD_3OD) spectrum of **10**

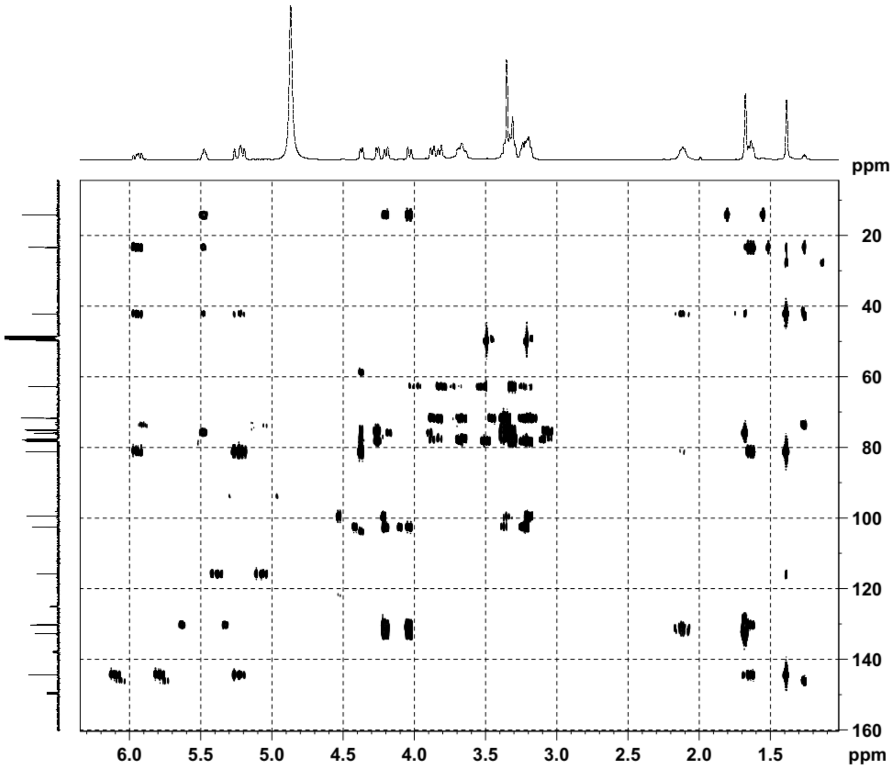

Figure S86 HMBC (CD_3OD) spectrum of **10**

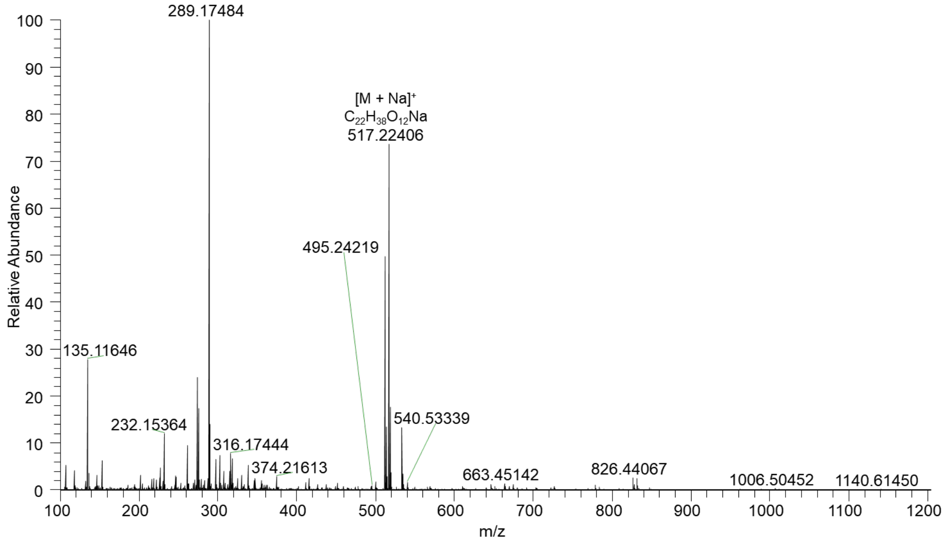

Figure S87 ESI-Q-Orbitrap-MS spectrum of **10**

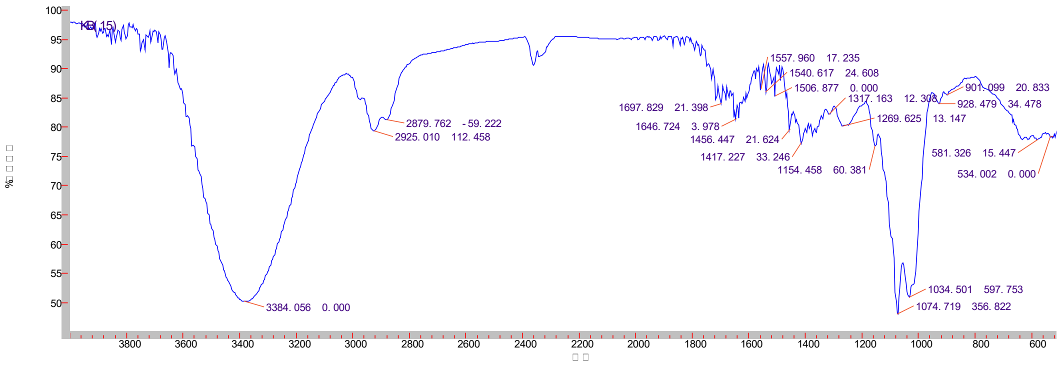

Figure S88 IR spectrum of **10**

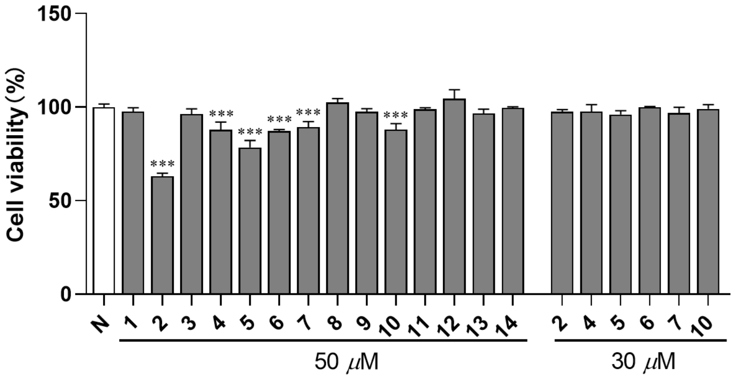

Figure S89 MTT assay of compounds **1–14**. Cell viability: percentage of normal group (set as 100%). Values represent the mean ± SD of six determinations. ***$P < 0.001$ (Difference between compound-treated group and normal group).

The physical data of compounds 1–14, 3a, 6a and 8a

*Dolilabterpenoside A (**1**)*

White powder; $[\alpha]^{25}_{D}$ +17.5 (*conc* 0.080, MeOH); CD (*conc* 0.001 M, MeOH) mdeg (nm): –0.55 (231); +1.15 (261); UV λ_{max} (MeOH) nm (log ε): 264 (4.62); IR n_{max} (KBr) cm^{-1}: 3381, 2932, 2886, 1693, 1634, 1602, 1454, 1379, 1075, 1021; ^{1}H NMR (CD_3OD, 500 MHz) and ^{13}C NMR (CD_3OD, 125 MHz) data: see Table 1; ESI-Q-Orbitrap MS: *m/z* 605.24481 [M – H]$^{-}$ (calcd for $C_{27}H_{41}O_{15}$, 605.24400).

*Dolilabterpenoside B (**2**)*

White powder; $[\alpha]_{D}^{25}$ –57.1 (conc 0.035, MeOH); UV λ_{max} (MeOH) nm (log ε): 231 (3.67), 282 (3.23); IR ν_{max} (KBr) cm^{-1}: 3368, 2927, 1703, 1606, 1560, 1518, 1455, 1277, 1161, 1126, 1067, 1046; ^{1}H NMR (CD_3OD, 500 MHz) and ^{13}C NMR (CD_3OD, 125 MHz) data: see Table 2; ESI-Q-Orbitrap MS: m/z 461.16696 [M – H]$^{-}$ (calcd for $C_{20}H_{29}O_{12}$, 461.16535).

*Dolilabterpenoside C$_1$ (**3**)*

White powder; $[\alpha]_{D}^{25}$ –50.0 (*conc* 0.032, MeOH); UV λ_{max} (MeOH) nm (log ε): 284 (2.96); IR ν_{max} (KBr) cm^{-1}: 3363, 2924, 1658, 1067, 1048; ^{1}H NMR (CD_3OD, 600 MHz) and ^{13}C NMR (CD_3OD, 150 MHz) data: see Table 3; ESI-Q-Orbitrap MS: *m/z* 439.18240 [M + COOH]$^{-}$ (calcd for $C_{18}H_{31}O_{12}$, 439.18100).

*Dolilabterpenoside C$_2$ (**4**)*

White powder; $[\alpha]_{D}^{25}$ –14.1 (*conc* 0.043, MeOH); IR ν_{max} (KBr) cm^{-1}: 3352, 2973, 2926, 2849, 1450, 1164, 1077, 1017, 923; ^{1}H NMR (CD_3OD, 500 MHz) and ^{13}C NMR (CD_3OD, 125 MHz) data: see Table 4; ESI-Q-Orbitrap MS: *m/z* 479.25037 [M + COOH]$^{-}$ (calcd for $C_{22}H_{39}O_{11}$, 479.24869).

*Dolilabterpenoside C_3 (**5**)*

White powder; ^{1}H NMR (CD_3OD, 600 MHz) δ_H: 7.08 (2H, dd, *J* = 1.2, 7.2 Hz, H-2,6), 7.29 (2H, dt, *J* = 1.2, 7.2 Hz, H-3,5), 7.00 (1H, tt, *J* = 1.2, 7.2 Hz, H-4), 4.85 (1H, d, *J* = 8.4 Hz, H-1'), 3.46 (1H, dd, *J* = 8.4, 9.0 Hz, H-2'), 3.44 (1H, dd, *J* = 9.0, 9.0 Hz, H-3'), 3.37 (1H, dd, *J* = 9.6, 9.6 Hz, H-4'), 3.55 (1H, m, H-5'), [3.60 (1H, dd, *J* = 6.6, 11.4 Hz), 4.02 (1H, dd, *J* = 1.8, 11.4 Hz), H_2-6'], 4.70 (1H, d, *J* = 1.8 Hz, H-1''), 3.83 (1H, dd, *J* = 1.8, 3.0 Hz, H-2''), 3.70 (1H, dd, *J* = 3.0, 9.6 Hz, H-3''), 3.36 (1H, dd, *J* = 9.6, 9.6 Hz, H-4''), 3.65 (1H, m, H-5''), 1.20 (3H, d, *J* = 6.0 Hz, H_3-6''); ^{13}C NMR (CD_3OD, 150 MHz) δ_C: 159.1 (C-1), 117.9 (C-2,6), 130.5 (C-3,5), 123.5 (C-4), 102.4 (C-1'), 75.0 (C-2'), 78.1 (C-3'), 71.6 (C-4'), 77.0 (C-5'), 67.9 (C-6'), 102.2 (C-1''), 72.2 (C-2''), 72.5 (C-3''), 74.1 (C-4''), 69.9 (C-5''), 18.0 (C-6''); ESI-Q-Orbitrap MS: *m*/*z* 447.15134 $[M + COOH]^-$ (calcd for $C_{19}H_{27}O_{12}$, 447.14970).

*Dolilabterpenoside D (**6**)*

White powder; ^{1}H NMR (CD_3OD, 600 MHz) δ_H: 7.41 (2H, br. d, *ca.* *J* = 7 Hz, H-2,6), 7.33 (2H, t like, *ca.* *J* = 7 Hz, H-3,5), 7.27 (1H, t like, *J* = 7 Hz, H-4), [4.64 (1H, d, *J* = 11.4 Hz), 4.88 (1H, d, *J* = 11.4 Hz), H_2-7], 4.32 (1H, d, *J* = 7.8 Hz, H-1'), 3.24 (1H, dd, *J* = 7.8, 9.0 Hz, H-2'), 3.34 (1H, dd, *J* = 9.0, 9.0 Hz, H-3'), 3.29 (1H, dd, *J* = 9.0, 9.0 Hz, H-4'), 3.38 (1H, m, H-5'), [3.64 (1H, dd, *J* = 6.0, 11.4 Hz), 4.00 (1H, dd, *J* = 1.8, 10.8 Hz), H_2-6'], 4.78 (1H, d, *J* = 1.2 Hz, H-1''), 3.87 (1H, dd, *J* = 1.2, 3.6 Hz, H-2''), 3.70 (1H, dd, *J* = 3.6, 9.6 Hz, H-3''), 3.38 (1H, *J* = 9.6, 9.6 Hz, H-4''), 3.69 (1H, m, H-5''), 1.27 (3H, d, *J* = 6.0 Hz, H_3-6''); ^{13}C NMR (CD_3OD, 150 MHz) δ_C: 138.9 (C-1), 129.4 (C-2,6, 3,5), 128.8 (C-4), 71.8 (C-7), 103.2 (C-1'), 75.1 (C-2'), 78.1 (C-3'), 71.8 (C-4'), 77.0 (C-5'), 68.2 (C-6'), 102.3 (C-1''), 72.3 (C-2''), 72.4 (C-3''), 74.1 (C-4''), 69.9 (C-5''), 18.1 (C-6''); ESI-Q-Orbitrap MS: *m*/*z* 461.16733 $[M + COOH]^-$ (calcd for $C_{20}H_{29}O_{12}$, 461.16535).

*Dolilabterpenoside E (**7**)*

White powder; ^{1}H NMR (DMSO-d_6, 500 MHz) δ_H: 7.27 (2H, m, H-2,6), 7.28 (2H, m, H-3,5),

7.27 (1H, t like, *ca.* J = 8 Hz, H-4), 2.86 (2H, t, J = 7.0 Hz, H-7), [3.68 (1H, q like, *ca.* J = 8 Hz), 3.89 (1H, q like, *ca.* J = 8 Hz), H_2-8], 4.19 (1H, d, J = 7.5 Hz, H-1'), 2.96 (1H, dd, J = 7.5, 8.5 Hz, H-2'), 3.14 (1H, dd, J = 8.5, 9.0 Hz, H-3'), 3.00 (1H, dd, J = 9.0, 9.0 Hz, H-4'), 3.27 (1H, m, H-5'), [3.43 (1H, dd, J = 6.5, 11.0 Hz), 3.82 (1H, br. d, *ca.* J = 11 Hz), H_2-6'], 4.60 (1H, br. s, H-1''), 3.61 (1H, br. d, *ca.* J = 4 Hz, H-2''), 3.42 (1H, dd, J = 3.5, 9.5 Hz, H-3''), 3.17 (1H, dd, J = 9.5, 9.5 Hz, H-4''), 3.45 (1H, m, H-5''), 1.13 (3H, d, J = 6.5 Hz, H_3-6''); ^{13}C NMR (DMSO-d_6, 125 MHz) δ_C: 138.6 (C-1), 128.8 (C-2,6), 128.1 (C-3,5), 125.9 (C-4), 35.6 (C-7), 69.4 (C-8), 102.8 (C-1'), 73.2 (C-2'), 76.5 (C-3'), 70.1 (C-4'), 75.3 (C-5'), 66.9 (C-6'), 100.7 (C-1''), 70.4 (C-2''), 70.6 (C-3''), 71.8 (C-4''), 68.2 (C-5''), 17.8 (C-6''); ESI-Q-Orbitrap MS: *m/z* 429.17685 [M – H]$^-$ ((calcd for $C_{20}H_{29}O_{10}$, 429.17552).

*Dolilabterpenoside F$_1$ (**8**)*

White powder; ^{1}H NMR (CD_3OD, 600 MHz) δ_H: 7.06 (2H, d, J = 8.0 Hz, H-2,6), 6.69 (2H, d, J = 8.0 Hz, H-3,5), 2.83 (2H, m, H_2-7), [3.68 (1H, m), 3.96 (1H, m), H_2-8], 4.28 (1H, d, J = 7.8 Hz, H-1'), 3.17 (1H, dd, J = 7.8, 9.0 Hz, H-2'), 3.33 (1H, dd, J = 9.0, 9.0 Hz, H-3'), 3.27 (1H, dd, J = 9.0, 9.6 Hz, H-4'), 3.37 (1H, m, H-5'), [3.61 (1H, dd, J = 6.0, 11.4 Hz), 3.97 (1H, m), H_2-6'], 4.74 (1H, d, J = 1.8 Hz, H-1''), 3.83 (1H, dd, J = 1.8, 3.6 Hz, H-2''), 3.67 (1H, dd, J = 3.6, 9.0 Hz, H-3''), 3.36 (1H, dd, J = 9.0, 9.0 Hz, H-4''), 3.66 (1H, m, H-5''), 1.25 (3H, d, J = 6.0 Hz, H_3-6''); ^{13}C NMR (CD_3OD, 150 MHz) δ_C: 130.7 (C-1), 131.0 (C-2,6), 116.2 (C-3,5), 156.8 (C-4), 36.5 (C-7), 72.3 (C-8), 104.5 (C-1'), 75.1 (C-2'), 78.1 (C-3'), 71.7 (C-4'), 76.9 (C-5'), 68.1 (C-6'), 102.3 (C-1''), 72.2 (C-2''), 72.4 (C-3''), 74.1 (C-4''), 69.8 (C-5''), 18.1 (C-6''); ESI-Q-Orbitrap MS: *m/z* 445.17215 [M – H]$^-$ (calcd for $C_{20}H_{29}O_{11}$, 445.17044).

*Dolilabterpenoside F$_2$ (**9**)*

White powder; ^{1}H NMR (CD_3OD, 600 MHz) δ_H: 5.95 (1H, d, J = 2.4 Hz, H-4), 6.18 (1H, d, J = 2.4 Hz, H-6), 3.99 (1H, sept, J = 6.6 Hz, H-2'), 1.15 (3H, d, J = 6.6 Hz, H_3-3'), 1.13 (3H, d, J = 6.6 Hz, H_3-4'), 5.04 (1H, d, J = 7.8 Hz, H-1''), 3.50 (1H, dd, J = 7.8, 9.0 Hz, H-2''), 3.47 (1H, dd, J = 9.0,

9.0 Hz, H-3''), 3.39 (1H, dd, *J* = 9.0, 9.6 Hz, H-4''), 3.46 (1H, m, H-5''), [3.72 (1H, dd, *J* = 6.0, 12.0 Hz), 3.91 (1H, dd, *J* = 1.8, 12.0 Hz), H_2-6'']; ^{13}C NMR (CD_3OD, 150 MHz) δ_C: 161.7 (C-1), 106.3 (C-2), 167.5 (C-3), 98.3 (C-4), 165.6 (C-5), 95.4 (C-6), 212.0 (C-1'), 40.5 (C-2'), 20.3 (C-3'), 19.5 (C-4'), 101.6 (C-1''), 74.9 (C-2''), 78.7 (C-3''), 71.2 (C-4''), 78.4 (C-5''), 62.5 (C-6''); ESI-Q-Orbitrap MS: *m*/*z* 357.11917 $[M-H]^-$ (calcd for $C_{16}H_{21}O_9$ 357.11801).

Dolilabterpenoside F_3 (***10***)

White powder; CD (*conc* 0.0027 M, MeOH) mdeg (λnm): +0.98 (271), –4.20 (304); ^{1}H NMR (CD_3OD, 600 MHz) δ_H: 5.96 (1H, d, *J* = 2.4 Hz, H-4), 6.19 (1H, d, *J* = 2.4 Hz, H-6), 3.90 (1H, sex, *J* = 6.6 Hz, H-2'), 1.38, 1.80 (1H each, both m, H_2-3'), 0.89 (3H, t, *J* = 7.8 Hz, H_3-4'), 1.13 (3H, d, *J* = 6.6 Hz, H_3-5'), 5.03 (1H, d, *J* = 7.2 Hz, H-1''), 3.52 (1H, dd, *J* = 7.2, 9.0 Hz, H-2''), 3.46 (1H, dd, *J* = 8.4, 9.0 Hz, H-3''), 3.39 (1H, dd, *J* = 8.4, 9.0 Hz, H-4''), 3.46 (1H, m, H-5''), [3.71 (1H, dd, *J* = 5.4, 12.0 Hz), 3.92 (1H, dd, *J* = 1.8, 12.0 Hz), H_2-6'']; ^{13}C NMR (CD_3OD, 150 MHz) δ_C: 161.8 (C-1), 106.9 (C-2), 167.5 (C-3), 98.4 (C-4), 165.6 (C-5), 95.4 (C-6), 211.8 (C-1'), 47.0 (C-2'), 28.3 (C-3'), 12.1 (C-4'), 16.9 (C-5'), 101.7 (C-1''), 74.8 (C-2''), 78.8 (C-3''), 71.3 (C-4''), 78.5 (C-5''), 62.5 (C-6''); ESI-Q-Orbitrap MS: *m*/*z* 371.13489 $[M-H]^-$ (calcd for $C_{17}H_{23}O_9$, 371.13365).

GA_8-2-O-β-D-glucopyranoside (***11***)

White powder, HRESIMS: *m*/*z* 525.19664 $[M-H]^-$ (calcd for $C_{25}H_{33}O_{12}$, M_r = 525.19665). ^{1}H NMR (CD_3OD, 500 MHz) δ_H: [1.75 (1H, m, 2.46 (1H, dd, *J* = 6.5, 13.5 Hz, H-1)], 3.88 (1H, ddd, *J* = 3.5, 6.5, 10.0 Hz, H-2), 3.83 (1H, d, *J* = 3.5, H-3), 3.28 (1H, d, *J* = 10.0 Hz, H-5], 2.53 (1H, d, *J* = 10.0 Hz, H-6), 1.85 (1H, m, H-9), 1.67, 1.87 (1H each, both m, H_2-11), [1.74 (1H, m, overlapped), 1.96 (1H, m), H_2-12], [1.77, 1.91 (1H each, both d, *J* = 10.5 Hz), H_2-14], [2.23, 2.37 (1H each, both br.d, *ca.* *J* = 16 Hz), H_2-15], 4.93, 5.19 (1H each, both br. s, H_2-17), 1.19 (1H, s, H-18), 4.38 (1H, d, *J* = 8.0 Hz, H-1'), 3.18 (1H, dd, *J* = 8.0, 8.5 Hz, H-2'), 3.35 (1H, dd, *J* = 8.5, 9.0 Hz, H-3'), 3.27 (1H, m, overlapped, H-4'), 3.27 (1H, m, overlapped, H-5'), [3.64 (1H, dd, *J* = 3.5, 12.0 Hz), 3.85 (1H, br. d, *ca.* *J* = 12 Hz), H_2-6']. ^{13}C NMR (CD_3OD, 125 MHz) δ_C: 35.5 (C-1), 76.2 (C-2), 70.8 (C-3), 54.6

(C-4), 52.9 (C-5), 52.4 (C-6), 176.0 (C-7), 51.1 (C-8), 53.2 (C-9), 95.3 (C-10), 18.3 (C-11), 39.7 (C-12), 78.8 (C-13), 46.2 (C-14), 44.2 (C-15), 158.2 (C-16), 107.4 (C-17), 15.2 (C-18), 103.1 (C-1'), 75.1 (C-2'), 77.8 (C-3'), 71.5 (C-4'), 78.1 (C-5'), 62.6 (C-6').

*(1′R,3′S,5′R,8′S,2Z,4E)-Dihydrophaseic acid (**12**)*

White powder, HRESIMS: *m/z* 443.19208 [M – H]⁻（calcd for $C_{21}H_{31}O_{10}$，M_r = 443.19117). ^{1}H NMR (CD_3OD, 500 MHz) δ_H: 5.76 (1H, s, H-2), 7.98 (1H, d, *J* = 16.0 Hz, H-4), 6.52 (1H, d, *J* = 16.0 Hz, H-5), 2.08 (3H, s, H_3-6), [1.79 (1H, dd, *J* = 10.5, 13.0 Hz), 1.98 (1H, dd, *J* = 7.0, 13.0 Hz), H_2-2'], 4.26 (1H, dddd, *J* = 7.5, 10.5, 13.0, 13.5 Hz, H-3'), [1.81 (1H, dd, *J* = 10.5, 13.0 Hz), 2.19 (1H, dd, *J* = 10.5, 13.0 Hz), H_2-4'], 3.76, 3.80 (1H each, both d, *J* = 7.5 Hz, H_2-7'), 1.17 (3H, s, H_3-9'), 0.94 (3H, s, H_3-10'), 4.36 (1H, d, *J* = 7.5 Hz, H-1''), 3.14 (1H, dd, *J* = 7.5, 8.5 Hz, H-2''), 3.36 (1H, dd, *J* = 8.0, 8.5 Hz, H-3''), 3.27 (1H, m, overlapped, H-4''), 3.27 (1H, m, overlapped, H-5''), [3.67 (1H, dd, *J* = 4.0, 11.5 Hz), 3.87 (1H, br. d, *ca.* *J* = 12 Hz), H_2-6'']. ^{13}C NMR (CD_3OD, 125 MHz) δ_C: 169.8 (C-1), 119.4 (C-2), 51.3 (C-3), 131.9 (C-4), 135.0 (C-5), 21.3 (C-6), 49.9 (C-1'), 42.8 (C-2'), 73.9 (C-3'), 42.8 (C-4'), 87.6 (C-5'), 77.1 (C-7'), 83.2 (C-8'), 19.7 (C-9'), 16.4 (C-10'), 103.0 (C-1''), 75.1 (C-2''), 78.0 (C-3''), 71.6 (C-4''), 77.9 (C-5''), 62.7 (C-6'').

*3,7-Dimethyl-oct-1-en-3,6,7-triol-6-O-β-D-glucopyranoside (**13**)*

White powder. ^{1}H NMR (CD_3OD, 500 MHz) δ_H: [5.01 (1H, br. d, *ca.* *J* = 11 Hz), 5.20 (1H, br. d, *ca.* *J* = 17 Hz), H_2-1], 5.91 (1H, dd, *J* = 11.0, 17.0 Hz, H-2), 1.54, 2.05 (1H each, both m, H_2-4), 1.54, 1.63 (1H each, both m, H_2-5), 3.45 (1H, br. d, *ca.* *J* = 7 Hz, H-6), 1.13 (3H, s, H_3-8), 1.16 (3H, s, H_3-9), 1.24 (3H, s, H_3-10), 4.34 (1H, d, *J* = 8.0 Hz, H-1'), 3.25 (1H, dd, *J* = 8.0, 8.5 Hz, H-2'), 3.37 (1H, dd, *J* = 8.0, 8.5 Hz, H-3'), 3.30 (1H, m, overlapped, H-4'), 3.30 (1H, m, overlapped, H-5'), [3.65 (1H, dd, *J* = 4.0, 12.0 Hz), 3.86 (1H, br. d, *ca.* *J* = 12 Hz), H_2-6']; ^{13}C NMR (CD_3OD, 125 MHz) δ_C: 112.0 (C-1), 146.6 (C-2), 73.9 (C-3), 39.5 (C-4), 27.1 (C-5), 90.5 (C-6), 73.7 (C-7), 26.6 (C-8), 24.5 (C-9), 27.9 (C-10), 105.2 (C-1'), 75.5 (C-2'), 78.0 (C-3'), 71.6 (C-4'), 78.1 (C-5'), 62.6 (C-6'). HRESIMS: *m/z* 395.19199 [M + COOH]⁻ (calcd for $C_{17}H_{31}O_{10}$, M_r = 395.19117).

*(3S)-6,7-Dihydroxy-dihydrolinalool-7-O-β-glucopyranoside (**14**)*

White powder. ^{1}H NMR (C_5D_5N, 600 MHz) δ_H: [5.14 (1H, dd, *J* = 2.0, 10.5 Hz), 5.59 (1H, dd,

J = 1.5, 17.5 Hz), H_2-1], 6.20 (1H, dd, *J* = 10.5, 17.5 Hz, H-2), 1.88, 2.14 (1H each, both m, H_2-4), 1.93, 1.95 (1H each, both m, H_2-5), 4.02 (1H, m, H-6), 1.49 (3H, s, H_3-8), 1.50 (3H, s, H_3-9), 1.48 (3H, s, H_3-10), 5.19 (1H, d, *J* = 7.8 Hz, H-1'), 4.04 (1H, dd, *J* = 7.8, 8.4 Hz, H-2'), 4.26 (1H, dd, *J* = 8.4, 9.6 Hz, H-3'), 4.24 (1H, dd, *J* = 9.6, 9.6 Hz, H-4'), 3.96 (1H, m, H-5'), [4.37 (1H, dd, *J* = 5.4, 12.0 Hz), 4.51 (1H, br. d, *ca.* *J* = 12 Hz), H_2-6']; ^{13}C NMR (C_5D_5N, 150 MHz) δ_C: 111.1 (C-1), 147.6 (C-2), 72.4 (C-3), 41.2 (C-4), 26.7 (C-5), 76.1 (C-6), 80.6 (C-7), 22.9 (C-8), 22.5 (C-9), 28.6 (C-10), 97.5 (C-1'), 75.6 (C-2'), 78.9 (C-3'), 71.7 (C-4'), 78.5 (C-5'), 62.7 (C-6'). HRESIMS: *m*/*z* 349.18640 [M – H]$^-$ (calcd for $C_{16}H_{29}O_8$, M_r = 349.18569).

Compound ***3a***

White powder; $[\alpha]_D^{25}$ +6.3 (*conc* 0.16, $CHCl_3$); ^{1}H NMR ($CDCl_3$, 500 MHz) δ_H: 3.78 (2H, d, *J* = 4.5 Hz, H_2-1), 3.49 (1H, t, *J* = 4.5 Hz, H-2), 1.43, 1.64 (1H each, both m, H_2-4), 2.05, 2.48 (1H each, both m, H_2-5), 5.12 (1H, t like, *ca.* *J* = 7 Hz, H-6), 1.69 (3H, s, H_3-8), 1.62 (3H, s, H_3-9), 1.25 (3H, s, H_3-10); ^{13}C NMR ($CDCl_3$, 125 MHz) δ_C: Table 3; ESI-Q-Orbitrap MS: *m*/*z* 187.13301 [M – H]$^-$ (calcd for $C_{10}H_{19}O_3$, 187.13287).

Compound ***6a***

White powder; $[\alpha]_D^{25}$ –6.6 (*conc* 0.060, $CHCl_3$); ^{1}H NMR ($CDCl_3$, 500 MHz) δ_H: 3.77 (2H, d, *J* = 5.0 Hz, H_2-1), 3.52 (1H, t, *J* = 5.0 Hz, H-2), 1.59 (2H, m, H_2-4), 2.06 (2H, m, H_2-5), 5.12 (1H, m, H-6), 1.69 (3H, s, H_3-8), 1.62 (3H, s, H_3-9), 1.18 (3H, s, H_3-10); ^{13}C NMR ($CDCl_3$, 125 MHz) δ_C: Table 3; ESI-Q-Orbitrap MS: *m*/*z* 187.13309 [M – H]$^-$ (calcd for $C_{10}H_{19}O_3$, 187.13287).

Compound ***8a***

White powder; $[\alpha]_D^{25}$ +8.1 (*conc* 0.070, $CHCl_3$); ^{1}H NMR (C_5D_5N, 600 MHz) δ_H: [5.17 (1H, dd, *J* = 2.4, 10.8 Hz), 5.58 (1H, dd, *J* = 2.4, 17.4 Hz), H_2-1], 6.17 (1H, dd, *J* = 10.8, 17.4 Hz, H-2), 1.81 (2H, m, H_2-4), 2.48 (2H, m, H_2-5), 5.77 (1H, t like, *ca.* *J* = 7 Hz, H-6), 4.30 (2H, s, H_2-8), 1.81 (3H, s, H_3-9), 1.47 (3H, s, H_3-10); ESI-Q-Orbitrap MS: *m*/*z* 169.12287 [M – H]$^-$ (calcd for $C_{10}H_{17}O_2$, 169.12231).
